# Preclinical efficacy of azacitidine and venetoclax for infant *KMT2A*-rearranged acute lymphoblastic leukemia reveals a new therapeutic strategy

**DOI:** 10.1038/s41375-022-01746-3

**Published:** 2022-11-15

**Authors:** Laurence C. Cheung, Carlos Aya-Bonilla, Mark N. Cruickshank, Sung K. Chiu, Vincent Kuek, Denise Anderson, Grace-Alyssa Chua, Sajla Singh, Joyce Oommen, Emanuela Ferrari, Anastasia M. Hughes, Jette Ford, Elena Kunold, Maria C. Hesselman, Frederik Post, Kelly E. Faulk, Erin H. Breese, Erin M. Guest, Patrick A. Brown, Mignon L. Loh, Richard B. Lock, Ursula R. Kees, Rozbeh Jafari, Sébastien Malinge, Rishi S. Kotecha

**Affiliations:** 1 Leukaemia Translational Research Laboratory, Telethon Kids Cancer Centre, Telethon Kids Institute, Perth, WA Australia; 2 Curtin Medical School, Curtin University, Perth, WA Australia; 3 Curtin Health Innovation Research Institute, Curtin University, Perth, WA Australia; 4 The University of Western Australia, Perth, WA Australia; 5 Department of Oncology-Pathology, Clinical Proteomics Mass Spectrometry, Karolinska Institutet, Science for Life Laboratory, Solna, Sweden; 6 University of Colorado Anschutz Medical Campus, Children’s Hospital Colorado, Aurora, CO USA; 7 Cancer and Blood Diseases Institute, Division of Oncology, Cincinnati Children’s Hospital Medical Center, Cincinnati, OH USA; 8 Department of Pediatrics, University of Cincinnati College of Medicine, Cincinnati, OH USA; 9 Division of Hematology, Oncology, Blood and Marrow Transplantation, Children’s Mercy Kansas City, Kansas City, MO USA; 10 Division of Pediatric Oncology, Sidney Kimmel Comprehensive Cancer Center, John Hopkins University, Baltimore, MD USA; 11 Division of Pediatric Hematology, Oncology, Bone Marrow Transplant and Cellular Therapy, Seattle Children’s Hospital, Seattle, WA USA; 12 Children’s Cancer Institute, Lowy Cancer Research Centre/School of Women’s and Children’s Health/UNSW Centre for Childhood Cancer Research, UNSW Sydney, Kensington, NSW Australia; 13 Department of Clinical Haematology, Oncology, Blood and Marrow Transplantation, Perth Children’s Hospital, Perth, WA Australia

**Keywords:** Acute lymphocytic leukaemia, Preclinical research

## Abstract

Infants with *KMT2A*-rearranged B-cell acute lymphoblastic leukemia (ALL) have a dismal prognosis. Survival outcomes have remained static in recent decades despite treatment intensification and novel therapies are urgently required. *KMT2A*-rearranged infant ALL cells are characterized by an abundance of promoter hypermethylation and exhibit high BCL-2 expression, highlighting potential for therapeutic targeting. Here, we show that hypomethylating agents exhibit in vitro additivity when combined with most conventional chemotherapeutic agents. However, in a subset of samples an antagonistic effect was seen between several agents. This was most evident when hypomethylating agents were combined with methotrexate, with upregulation of ATP-binding cassette transporters identified as a potential mechanism. Single agent treatment with azacitidine and decitabine significantly prolonged in vivo survival in *KMT2A*-rearranged infant ALL xenografts. Treatment of *KMT2A*-rearranged infant ALL cell lines with azacitidine and decitabine led to differential genome-wide DNA methylation, changes in gene expression and thermal proteome profiling revealed the target protein-binding landscape of these agents. The selective BCL-2 inhibitor, venetoclax, exhibited in vitro additivity in combination with hypomethylating or conventional chemotherapeutic agents. The addition of venetoclax to azacitidine resulted in a significant in vivo survival advantage indicating the therapeutic potential of this combination to improve outcome for infants with *KMT2A*-rearranged ALL.

## Introduction

Treatment for childhood B-cell acute lymphoblastic leukemia (ALL) has evolved over the last 70 years, leading to 5-year overall survival rates of over 90% [1]. However, infants diagnosed at less than one year of age constitute a subgroup with significantly inferior outcomes [2, 3]. The *KMT2A*-rearrangement is an aggressive driver present in ALL cells of up to 80% of infants and is associated with chemo-resistance and high rates of relapse. Coupled with the increased vulnerability of infants to treatment-related toxicity, 5-year event-free survival (EFS) remains less than 40% [4]. Novel therapeutic strategies are urgently required to improve outcomes.

*KMT2A*-rearranged infant ALL cells are characterized by an abundance of promoter hypermethylation which, in the classical model of methylation, can lead to silencing of genes, including tumor suppressor genes [5–7]. High BCL-2 expression has also been identified in *KMT2A*-rearranged ALL [8–10], and the *KMT2A-AFF1* fusion has been shown to upregulate *BCL-2* expression by promoting DOT1L-mediated H3K79 methylation at the *BCL-2* locus [10]. BCL-2 family proteins regulate the intrinsic apoptotic pathway by integrating diverse pro-survival or pro-apoptotic intracellular signals, with BCL-2 a key anti-apoptotic regulator within this pathway. Hypomethylating agents have been successfully used in combination with the BCL-2 inhibitor, venetoclax, for the treatment of patients with acute myeloid leukemia (AML) [11, 12]. In the current study we sought to investigate the efficacy of hypomethylating agents and of venetoclax for infant ALL. We discovered that azacitidine and decitabine were highly effective in our infant *KMT2A*-rearranged preclinical models, supporting the rationale for investigation of azacitidine in the Children’s Oncology Group (COG) AALL15P1 pilot study for infants with *KMT2A*-rearranged ALL (NCT02828358). Venetoclax was shown to enhance the efficacy of azacitidine in *KMT2A*-rearranged infant ALL xenografts, highlighting the therapeutic potential of this combination for infants with *KMT2A*-rearranged ALL.

## Materials and methods

### Assessment of in vitro sensitivity and synergy

All in vitro sensitivity and synergy experiments were performed on an extensively characterized panel of eight patient-derived *KMT2A*-rearranged infant ALL cell lines (PER cell lines) [13, 14], two commercially available *KMT2A*-rearranged infant ALL cell lines (ALL-PO and KOPN-8), and cells from four *KMT2A*-rearranged infant ALL patient-derived xenografts (MLL-5, MLL-7, MLL-14, and LR-iALL2) [15, 16]. Assessment of in vitro sensitivity to azacitidine, decitabine, zebularine (Selleck Chemicals) and venetoclax (Active Biochem) and their synergy with each of the nine conventional chemotherapy agents used to treat infants with ALL, and synergy of each of the hypomethylating agents with venetoclax was performed as previously described [13]. In vitro drug combination studies were analyzed using SynergyFinder 2.0 [17]. Western blots were performed to detect DNMT1 levels following incubation with azacitidine and decitabine. Apoptosis and necrosis assays were performed using the RealTime-Glo™ Annexin V Apoptosis and Necrosis kit (Promega) according to the manufacturer’s instructions (Supplementary Methods).

### Assessment of in vivo efficacy

In vivo experiments were performed using five xenograft models (PER-785, MLL-5, MLL-7, MLL-14 and LR-iALL2) [15, 16, 18], to determine the maximum tolerated dose (MTD) of azacitidine and decitabine, response to single agent drug treatment by EFS, and efficacy of azacitidine in combination with venetoclax (Supplementary Methods).

### Differential DNA methylation and transcriptomic profiling

Six cell lines were used for comparison of genome-wide DNA methylation and gene expression profiling following treatment with each hypomethylating agent in comparison to untreated control (Supplementary Methods). DNA methylation assays were performed using the Infinium MethylationEPIC BeadChip (Illumina) as per manufacturer’s instructions by the Australian Genome Research Facility. Following quality control checks, 773,929 probes were taken forward for downstream analyses (Supplementary Methods). Stranded poly A RNA sequencing was performed using a NovaSeq 6000 (Illumina) as per manufacturer’s instructions by the Australian Genome Research Facility. Raw sequencing reads were subjected to demultiplexing, quality control, trimming, alignment (hg38 human genome assembly), and transcriptome assembly. Differential expression analysis was performed using the edgeR package and raw counts were normalized using the trimmed mean of M-values method (Supplementary Methods).

### Gene expression analysis

Transcript abundance of candidate genes following treatment with azacitidine and decitabine was validated in triplicate by real-time quantitative polymerase chain reaction (PCR) in comparison to untreated control (Supplementary Methods). Relative gene expression quantification was performed using the 2^−ΔΔ*CT*^ method and fold change ratios were determined by log_2_ transformation of such values [19].

### Thermal proteome profiling

Thermal proteome profiling (TPP) over a temperature range was performed with minor modifications to previously described, using the ALL-PO cell line [20]. Online liquid chromatography tandem mass spectrometry was performed using a Dionex UltiMate™ 3000 RSLCnano System coupled to a Q-Exactive-HF mass spectrometer (Thermo Fisher Scientific) [20]. Analyses of the acquired mass spectrometry TPP data for the identification of the drug targets was performed as previously described (Supplementary Methods) [21, 22]. Cellular thermal shift assay (CETSA) temperature range and dose response time course experiments using western blotting readout were used to validate target engagement (Supplementary Methods).

## Results

### Effects of hypomethylating agents in vitro and in combination with conventional chemotherapeutic agents

**Fig. 1:**
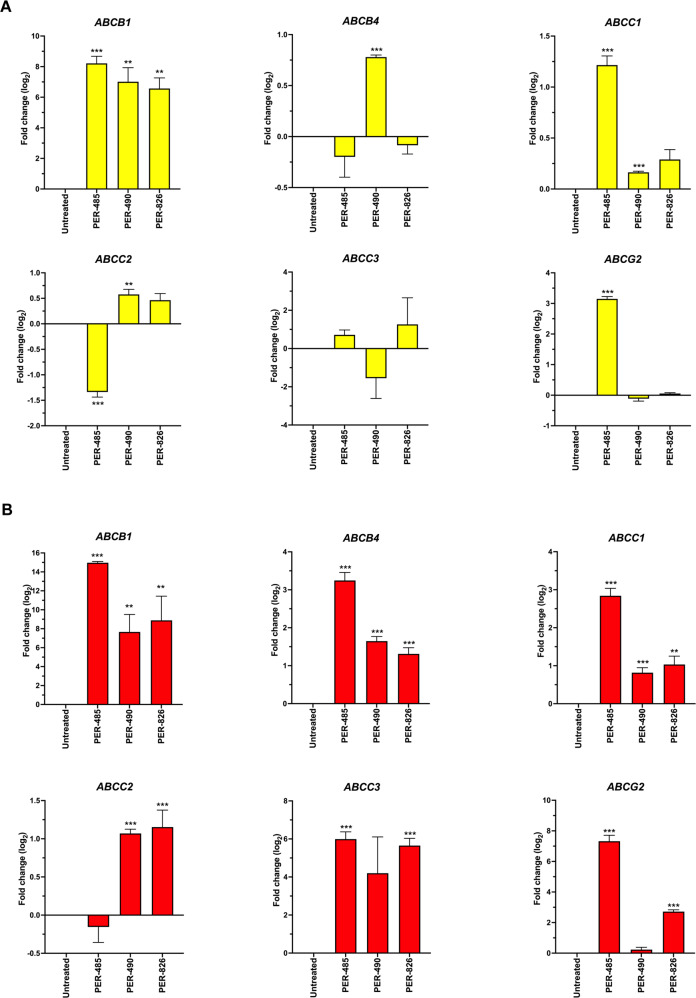
Treatment with hypomethylating agents induces upregulation of ATP-binding cassette transporters. Expression levels of ATP-binding cassette transporter genes of three *KMT2A*-rearranged infant ALL cell lines (PER-485, PER-490 and PER-826), incubated with IC_10-40_ concentrations of (**A**) azacitidine or (**B**) decitabine for 72 h were measured by real-time quantitative polymerase chain reaction. The bar plots depict mean log_2_ fold change of three biological replicates compared to untreated cells with standard error of the mean displayed for each sample. **p < 0.01, ***p < 0.001.

To identify the in vitro effect of hypomethylating agents for *KMT2A*-rearranged infant ALL, we individually tested azacitidine, decitabine and zebularine against a representative panel of 14 cellular models. Half maximum inhibitory concentration (IC_50_) ranged between 6.1–74.0 µM for azacitidine and 3.8–49.3 µM for decitabine-sensitive models, with resistance (>100 µM) demonstrated in the majority of models for zebularine (Supplementary Table 1). Azacitidine and decitabine induced apoptosis followed by necrosis at the IC_50_ for each cellular model (Supplementary Figs. 1–2). Expression of DNMT1 was significantly reduced after treatment with low dose (1.5 µM) azacitidine and decitabine (Supplementary Fig. 3). These findings are consistent with the knowledge that hypomethylating agents cause cell death at high concentrations whereas DNA hypomethylation is more effective with low concentrations [23]. Combination drug testing for each hypomethylating agent generally indicated an additive effect with most of the conventionally used chemotherapeutic agents. However, in a subset of samples an antagonistic effect was seen between several agents. This was most evident when hypomethylating agents were combined with methotrexate (Table 1, Supplementary Table 2, Supplementary Figs. 4–5). Further investigation of this finding identified that treatment with hypomethylating agents led to an increased expression of several ATP-binding cassette transporters (Fig. 1).

**Table 1:**
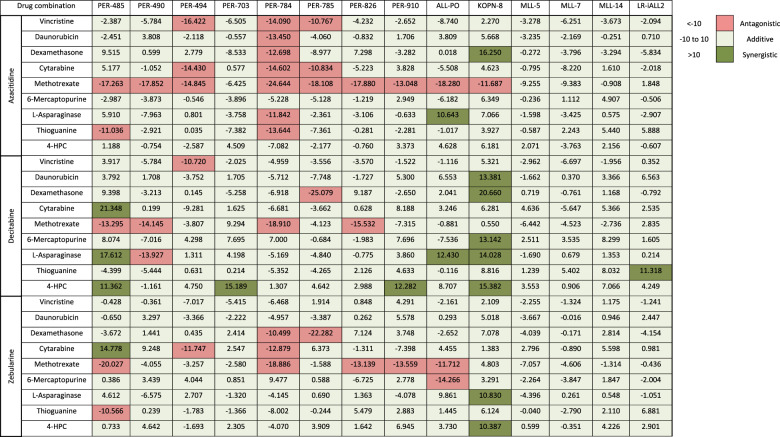
Total in vitro synergy scores between hypomethylating drugs in combination with conventional chemotherapeutic agents.

### Azacitidine and decitabine exhibit marked in vivo efficacy in *KMT2A*-rearranged infant ALL xenografts

**Fig. 2:**
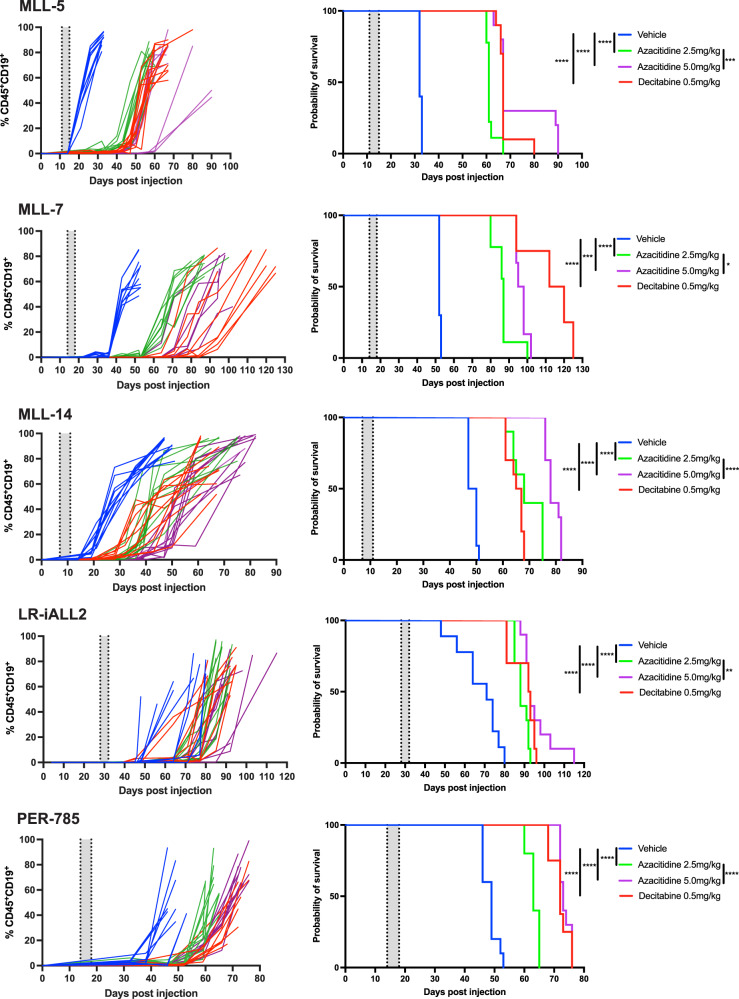
Azacitidine and decitabine prolong survival in *KMT2A*-rearranged infant acute lymphoblastic leukemia xenografts. Mice injected with leukemia cells were treated with vehicle control, 2.5 mg/kg azacitidine, 5 mg/kg azacitidine or 0.5 mg/kg decitabine once daily for five days when the percentage of human CD19^+^ CD45^+^ cells reached 1% in the bone marrow. Five xenograft models were used: MLL-5, MLL-7, MLL-14, LR-iALL2 and PER-785. Left panels: Percentage of human CD19^+^ CD45^+^ cells in peripheral blood over time. Right panels: Kaplan–Meier survival curves of the treated and control mice (*n* = 8–10 mice/group). The gray shaded areas indicate the treatment periods. *p < 0.05, **p < 0.01, ***p < 0.001, ****p < 0.0001.

We next determined the efficacy of azacitidine and decitabine in vivo. The MTD of azacitidine was established as 5.0 mg/kg and the MTD of decitabine as 0.5 mg/kg in the PER-785 xenograft. Leukemia-bearing mice receiving 8.0 mg/kg of azacitidine were euthanized early, on day 5 of therapy, due to excessive morbidity, including anorexia, weight loss and lack of vigor. Decitabine was well tolerated at all dose levels. A significant survival benefit was seen in all five xenograft models treated at low disease burden with azacitidine at both 2.5 mg/kg and 5.0 mg/kg and with decitabine at 0.5 mg/kg (Fig. 2). A dose-dependent extension of survival was seen for azacitidine treated mice, with the 5.0 mg/kg treated groups exhibiting significantly longer survival compared to the 2.5 mg/kg groups for each xenograft model (Fig. 2).

### Treatment with azacitidine and decitabine results in differential methylation

**Fig. 3:**
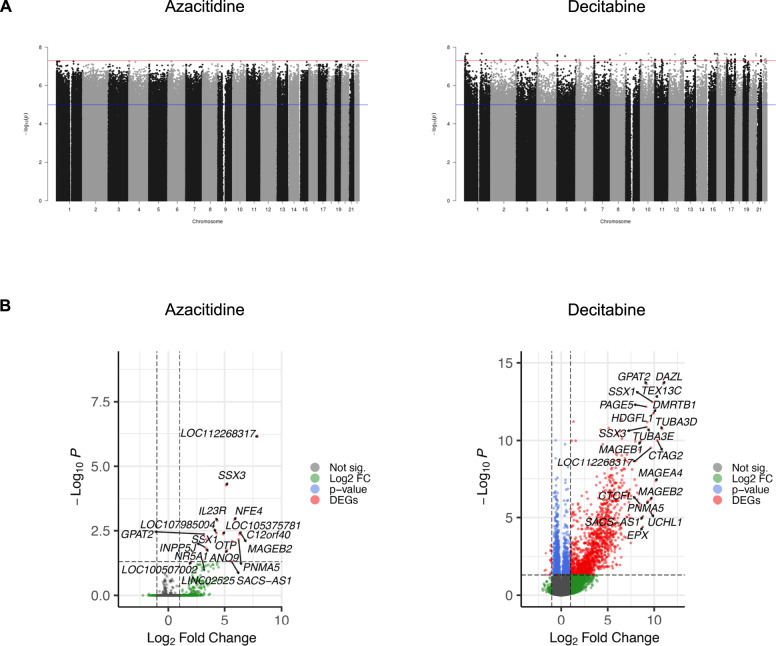
Treatment with azacitidine and decitabine results in differential methylation and transcriptional changes. **A** Manhattan plots showing differential methylation profiles across genome-wide CpG sites of six *KMT2A*-rearranged infant acute lymphoblastic leukemia cell lines treated at low dose (1.5 µM) with the hypomethylating agents azacitidine and decitabine in comparison to untreated controls. Differentially methylated CpG sites were mapped to their corresponding loci across the genome (*x* axis) and plotted against their significance levels (-log_10_ transformed *p*-values) (*y* axis), from comparisons between treated and untreated cell lines. The red line indicates genome-wide significance threshold of 5 × 10^−8^, and the blue line indicates suggestive significance, valued at 1 × 10^−5^. **B** Volcano plots highlighting the top differentially expressed genes from the merged transcriptome of all six cell lines following treatment with azacitidine and decitabine. Horizontal and vertical dashed lines indicate fold change and significance thresholds.

To determine the effect on CpG methylation, DNA methylation profiling was performed following treatment with low dose azacitidine (1.5 µM), decitabine (1.5 µM) or zebularine (6.0 µM) for 72 h in six *KMT2A*-rearranged infant ALL cell lines in comparison to untreated controls. Treatment resulted in differential methylation of many CpG sites following treatment with low dose azacitidine and decitabine (Fig. 3A), however there were no significant differentially methylated sites across the genome following treatment with low dose zebularine (Supplementary Fig. 6A). CpG sites were grouped into differentially hypomethylated regions, based on a threshold of over 5 differentially demethylated CpG sites spanning each region. A total of 617 and 471 differentially methylated regions were significantly hypomethylated in azacitidine- and decitabine-treated cells, respectively, compared to untreated controls (Supplementary Tables 3–4). These hypomethylated regions were common to all cell lines following treatment. There were 381 genes within these differentially hypomethylated regions following treatment with azacitidine and 275 following treatment with decitabine, of which 195 genes were common (Supplementary Table 5).

### Transcriptomic profiling reveals a higher effect of hypomethylation on gene expression by decitabine

To assess transcriptional changes induced by azacitidine and decitabine, we performed RNA sequencing of the six *KMT2A*-rearranged infant ALL cell lines following treatment with low dose azacitidine (1.5 µM) or decitabine (1.5 µM) in comparison to untreated controls. Treatment with low dose decitabine had a more marked effect on gene expression than azacitidine; this was evident both when merging the transcriptome of all six cell lines and also according to each individual cell line (Fig. 3B; Supplementary Fig. 6B, C; Supplementary Tables 6–7). Gene set enrichment analysis of significant differentially expressed genes revealed a much higher effect of decitabine on activating/inactivating annotated gene sets or cellular networks than azacitidine (Supplementary Table 8, Supplementary Fig. 6D).

Of genes located within hypomethylated regions following treatment, transcriptional profiling identified 11 genes to be significantly upregulated following treatment with decitabine, whereas there were no correlations following treatment with azacitidine (Supplementary Fig. 6E). For validation, we performed real-time quantitative PCR in our extended cohort of 13 *KMT2A*-rearranged infant ALL cellular models on three genes, *MMP15, BAIAP3* and *CD82*, confirming significant upregulation of these genes via hypomethylation following treatment with decitabine (Supplementary Fig. 6F).

### TPP of azacitidine and decitabine identifies novel binding targets

**Fig. 4:**
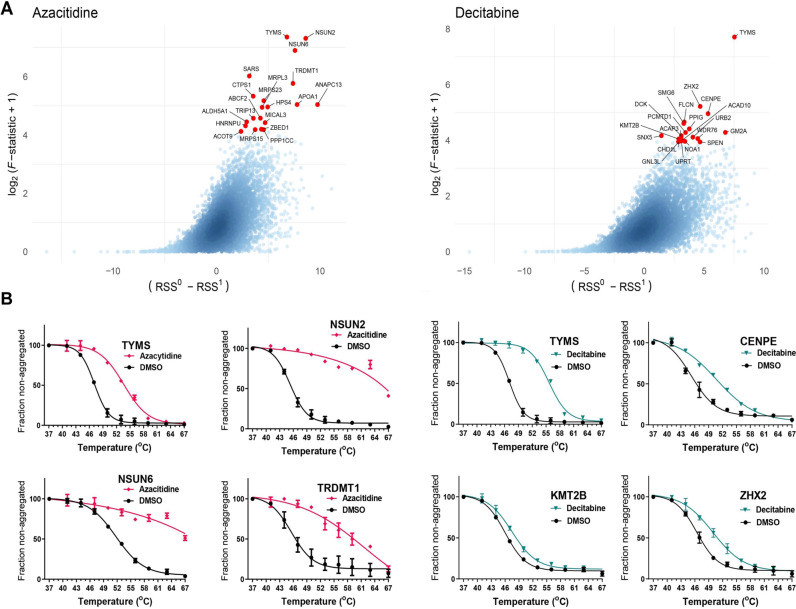
Thermal proteome profiling of the ALL-PO *KMT2A*-rearranged infant acute lymphoblastic leukemia cell line treated with azacitidine or decitabine reveals novel target proteins. **A** Volcano plot of the results obtained from the non-parametric analysis of response curves of thermal proteome profiling data for azacitidine and decitabine with selected significant hits annotated and highlighted in red. **B** Melt curves showing changes in aggregation temperature for TYMS, NSUN2, NSUN6 and TRDMT1 from azacitidine (magenta) and DMSO (black) treated ALL-PO cells. Melt curves showing changes in aggregation temperature for TYMS, CENPE, KMT2B and ZHX2 from decitabine (cyan) and DMSO (black) treated ALL-PO cells.

Insufficient characterization of drugs and their mechanism of action has been linked to costly clinical failure and approval rates. Drugs may have multiple primary and secondary targets, therefore it is important to comprehensively identify their targets to elucidate their mechanism of action. TPP and CETSA have emerged as powerful label-free tools to investigate target engagement of small molecule inhibitors at a proteome-wide level [24, 25]. Upon binding of a small molecule to a protein the thermal stability and aggregation temperature of the target protein is altered, and this change in aggregation temperature can be detected by various means such as western blotting [24], AlphaScreen [26], and mass spectrometry [25, 27]. We therefore investigated the proteome-wide target landscape of azacitidine and decitabine in ALL-PO, an infant ALL cell line which harbors the *KMT2A-AFF1* fusion. In total, TPP identified and quantified 6021 proteins for azacitidine and 5982 proteins for decitabine (Supplementary Tables 9–12). The significance threshold (*p*-value) was calculated using a non-parametric analysis [21]. Identified proteins with melt curves in both replicates of treatment and control and *p* ≤ 0.01 were selected for further analysis. These criteria were fulfilled for 218 proteins for azacitidine and 166 proteins for decitabine (Fig. 4A, Supplementary Fig. 7A, B, Supplementary Table 13). There was an overlap of 35 proteins between the hits for azacitidine and decitabine (Supplementary Table 13) which were mainly enriched for proteins involved in purine and pyrimidine metabolism, e.g., TYMS (Fig. 4A, B), DCK, UPRT and CTPS1 (Supplementary Fig. 7C).

It is well known that azacitidine is mainly incorporated into RNA, affecting RNA modifications and translation, however it is unclear which protein(s) are responsible or affected upon incorporation of azacitidine into RNA. Here, among the unique hits for azacitidine, we identified several RNA methyltransferases, such as NSUN2, NSUN6 and TRDMT1 (Fig. 4B). Among the hits for decitabine we also identified changes in the thermal stability for CENPE, ZHX2 and KMT2B (Fig. 4B).

CETSA and western blot analysis of azacitidine-treated ALL-PO cells validated the thermal stabilization of NSUN2 and TYMS by azacitidine (Supplementary Fig. 8A). Conversely, when lysates from ALL-PO cells were treated with azacitidine, NSUN2 and TYMS were not stabilized (Supplementary Fig. 8B) indicating that intracellular modification of azacitidine is necessary for its thermal stabilization of NSUN2 and TYMS. To investigate further, we performed a dose response CETSA experiment of ALL-PO cells treated with varying concentrations of azacitidine across multiple time points at 48 °C. The results demonstrated that azacitidine-mediated thermal stabilization of NSUN2 was time-dependent, further strengthening that intracellular modification of azacitidine is necessary for thermal stabilization of NSUN2 (Supplementary Fig. 8C).

### Venetoclax synergizes with hypomethylating and conventional chemotherapeutic agents in vitro

Given that hypomethylating agents have undergone extensive clinical investigation in combination with venetoclax and represents a therapeutic option for adults with AML [11], we next sought to investigate whether there was a combinatorial role for venetoclax in infants with *KMT2A*-rearranged ALL. Venetoclax demonstrated low IC_50_ in vitro, ranging between 0.003–6.350 µM (Supplementary Table 1). In order to confirm that venetoclax binds to BCL-2 we performed dose response CETSA in ALL-PO cells followed by western blot analysis, which demonstrated dose-dependent thermal stabilization of BCL-2 following venetoclax treatment (Supplementary Fig. 9). Venetoclax was shown to induce apoptosis followed by necrosis at the IC_50_ for each cellular model (Supplementary Fig. 10). In vitro drug combination testing revealed that venetoclax was either additive or synergistic when tested in combination with each of the three hypomethylating agents and each of the nine conventional chemotherapeutic agents in all 14 cellular models (Table 2, Supplementary Table 14, Supplementary Fig. 11).

**Table 2:**
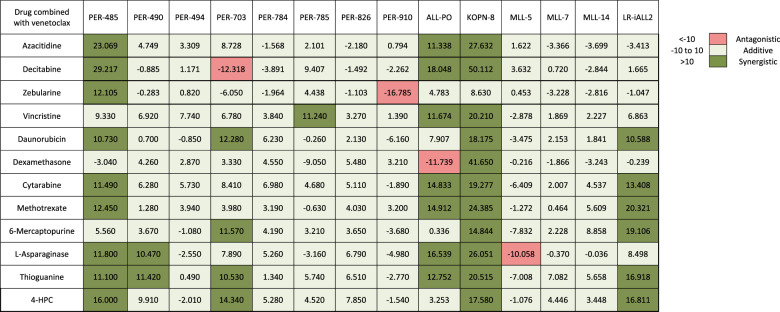
Total in vitro synergy scores between venetoclax in combination with hypomethylating or conventional chemotherapeutic agents.

### Azacitidine combined with venetoclax prolongs survival in *KMT2A*-rearranged infant ALL xenografts

**Fig. 5:**
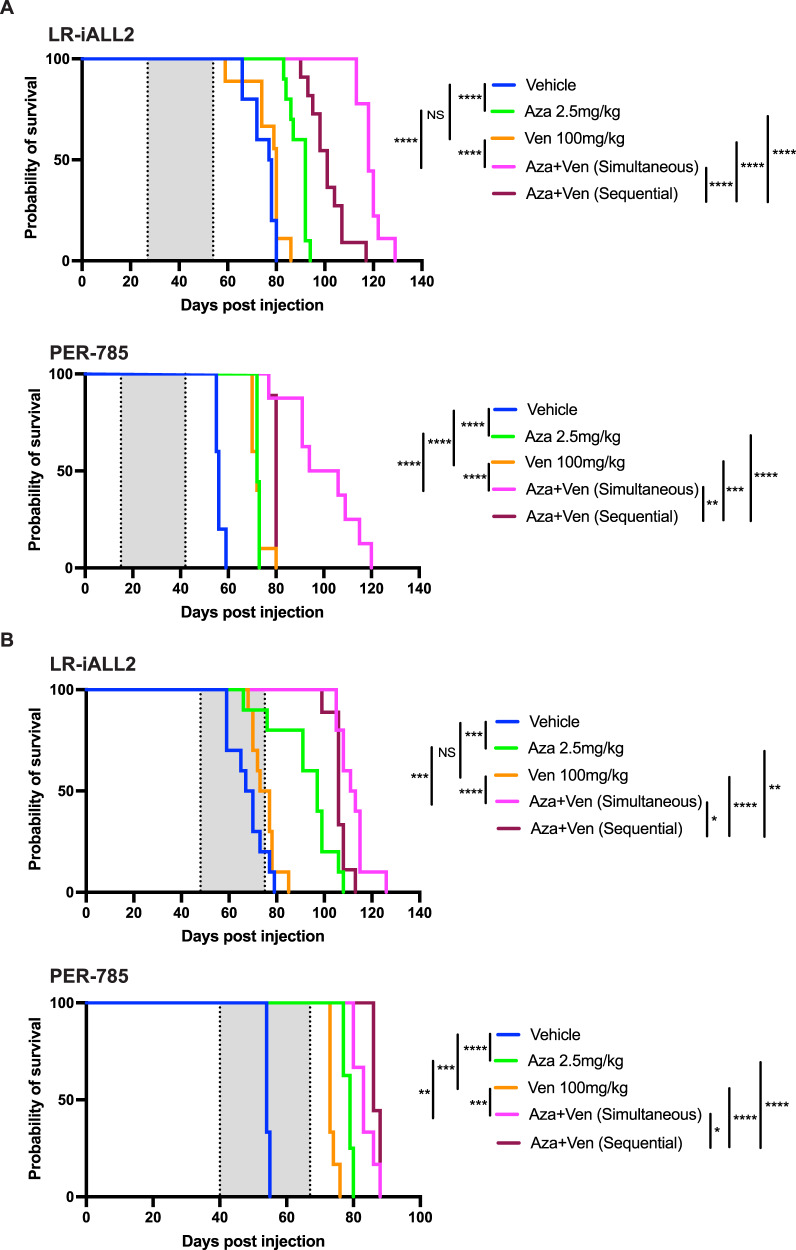
Azacitidine combined with venetoclax improves survival in *KMT2A*-rearranged infant acute lymphoblastic leukemia xenografts. Kaplan–Meier survival curves of leukemia-bearing mice treated with 2.5 mg/kg azacitidine once daily for five days, 100 mg/kg venetoclax once daily for 21 days, simultaneous administration of 2.5 mg/kg azacitidine once daily for five days in combination with 100 mg/kg venetoclax once daily for 21 days, sequential administration of 2.5 mg/kg azacitidine once daily for five days followed by 100 mg/kg venetoclax once daily for 21 days, and vehicle control (*n* = 9–10 mice/group). Two xenograft models were used: LR-iALL2 and PER-785. Treatment commenced at (**A**) low disease burden when the percentage of human CD19^+^ CD45^+^ cells reached 1% in the bone marrow and (**B**) high disease burden when the percentage of human CD19^+^ CD45^+^ cells reached 1% in the peripheral blood. The gray shaded areas indicate the treatment periods, with the first dotted line representing the start of treatment for all cohorts and the last dotted line representing the end of treatment for sequential administration cohort. *p < 0.05, **p < 0.01, ***p < 0.001, ****p < 0.0001.

Finally, we sought to determine the in vivo efficacy of azacitidine in combination with venetoclax. Azacitidine was prioritized and selected for further assessment in combination with venetoclax as any benefit identified would be readily translatable into clinical practice given that the tolerability of azacitidine is currently being investigated in the COG AALL15P1 trial for infants with *KMT2A*-rearranged ALL (NCT02828358). The 2.5 mg/kg azacitidine dose was selected to mimic the same dose and schedule used in AALL15P1 and to abrogate risk of toxicity with combination therapy at higher doses. The combination of azacitidine and venetoclax produced a significant survival advantage in both LR-iALL2 and PER-785 xenograft models compared to each individual agent alone. The effect was seen for treatment of disease at both low and high burden and also when the agents were administered simultaneously or sequentially (Fig. 5A, B).

## Discussion

Improvements in outcome for children with ALL has largely been achieved through randomized clinical trials optimizing conventional chemotherapy, improvements in risk stratification based on blast genetics and early response to therapy and delivering appropriately intense therapy. However, infants diagnosed with *KMT2A*-rearranged ALL remain an exception to this success, with 5-year EFS less than 40% [4]. Clinical trials for infant ALL have increased the dose-intensity of multi-agent chemotherapy, however, they have not improved outcome due to high relapse rates and dose-limiting toxicities of conventional chemotherapeutic agents, highlighting the urgent need to identify innovative therapies to advance outcomes for infant ALL [2, 3].

*KMT2A*-rearranged infant ALL cells are characterized by an abundance of promoter hypermethylation which, in the classical model of methylation, can lead to silencing of genes, including tumor suppressor genes [5–7]. This has led to hypomethylating agents emerging as promising therapeutic candidates, with azacitidine currently being investigated in the COG AALL15P1 pilot study (NCT02828358), for infants with *KMT2A*-rearranged ALL. However, there remains a distinct lack of preclinical evidence for use of azacitidine in this patient population. In addition to targeting hypermethylation, inhibition of BCL-2 has also been proposed as a novel strategy to treat *KMT2A-*rearranged infant ALL [28–30]. Therefore, the aims of this study were to (i) investigate the benefit of hypomethylating agents for infants with *KMT2A*-rearranged ALL, (ii) elucidate the effect of these drugs on the methylome, transcriptome and identify their target protein binding landscape and (iii) determine the benefit of adding the selective BCL-2 inhibitor, venetoclax, to treatment for infants with *KMT2A*-rearranged ALL.

We identified that treatment with single agent azacitidine and decitabine significantly improved EFS in *KMT2A*-rearranged infant ALL xenografts. Previously, two studies have shown mild delays in leukemia progression following treatment with decitabine in xenografts. Both used lower decitabine doses with less frequent administration, which may account for the modest effects compared to our study [31, 32]. In addition, both studies did not use patient-derived xenografts established from infants, with xenografts established from the RS4;11 and SEM *KMT2A*-rearranged B-ALL cell lines, derived from a 32-year-old adult and 5-year-old child respectively. The current COG AALL15P1 study was designed to test the tolerability of adding four five-day courses of azacitidine administered at 2.5 mg/kg/dose prior to chemotherapy following induction therapy. Our study has thus mimicked the same schedule and investigated efficacy in a similar setting of low disease burden as that of a patient on this study. Coupled with the predominance of additivity or synergy shown by the hypomethylating agents in combination with conventional chemotherapeutic agents in vitro, our study is the first to provide strong in vivo evidence to support the use of azacitidine or decitabine to treat infants with *KMT2A*-rearranged ALL. Notably, AALL15P1 was primarily designed for assessment of safety and feasibility, and studies to determine efficacy of azacitidine or decitabine should be considered in future clinical trial designs. In addition, the marked in vitro antagonism between methotrexate and the hypomethylating agents represents another clinically significant finding. The increased expression of several ATP-dependent efflux pumps following treatment with hypomethylating agents may contribute to reduced methotrexate sensitivity, as previously described in a breast cancer cell line [33], however further work is necessary to elucidate additional mechanisms that may contribute to this antagonistic effect. Nonetheless, our findings guard against concomitant administration of methotrexate with hypomethylating agents for infants with *KMT2A*-rearranged ALL. Furthermore, several other conventional chemotherapeutic agents exhibited antagonism with hypomethylating agents in a small subset of samples (PER-494, PER-784 and PER-785). This finding requires further exploration as it may indicate that a subpopulation of infants may not derive benefit from such combination therapy.

Similar to previous studies, we independently demonstrated differential methylation profiles following treatment with hypomethylating agents in a unique panel of extensively characterized *KMT2A*-rearranged infant ALL cell lines. These profiles revealed common gene targets of hypomethylation between azacitidine and decitabine suggesting shared pathways being regulated for both hypomethylating agents. However, we were unable to recapitulate the effects previously reported following treatment with zebularine [5, 7, 34]. In addition to the effect on methylation in *KMT2A*-rearranged infant ALL cells by azacitidine and decitabine, we investigated the effect of these hypomethylating drugs on genome-wide gene expression in our panel of cell lines. Decitabine induced a stronger effect on gene expression than azacitidine, which displayed a subtle and variable effect on gene expression across these cell lines. Such observations have also been reported at the transcriptome and cell-surface proteome level following treatment of AML cell lines with azacitidine at low doses [35]. In addition, we were only able to identify a small proportion of genes that were upregulated and located within hypomethylated regions following treatment. Our findings are supported by more recent studies, which indicate that the mechanisms for epigenetic regulation in infants with ALL extend beyond the classical model of methylation and gene expression and are more complex than previously thought [36, 37].

To further characterize the molecular impact of azacitidine and decitabine in *KMT2A*-rearranged infant ALL cells, TPP analysis was conducted to identify the protein binding targets of each drug. Using TPP as a label-free approach, we identified binding of azacitidine to several RNA methyltransferases, including NSUN2, NSUN6 and TRDMT1. RNA methyltransferases play an important role in epigenetic regulation, maintaining RNA stability, splicing, transport, localization and translation [38, 39]. These results suggest that there is a possible early immediate effect of azacitidine treatment acting through RNA methylation modifications. Furthermore, we discovered that azacitidine also binds to TYMS, increasing its aggregation temperature. We have previously shown that decitabine, following intracellular modification, binds to TYMS using a high throughput CETSA-based screen with the K562 chronic myeloid leukemia cell line [26]. In this current study, we used an unbiased approach with TPP to confirm this finding in the ALL-PO infant *KMT2A*-rearranged ALL cell line. TYMS is a pivotal protein in the thymidylate biosynthesis pathway and maintaining the dTMP pool which is necessary for DNA replication and repair. TYMS is a widely used cancer therapy target and thus its inhibition could contribute to the therapeutic effects of azacitidine and decitabine. Whilst our study has identified the protein binding target landscape for azacitidine and decitabine in *KMT2A*-rearranged infant ALL cells, further studies are required to functionally validate the biological effect of these interactions.

We previously demonstrated single-agent activity of venetoclax in sensitive infant *KMT2A*-rearranged patient-derived xenograft models [30]. In this study, we extended these findings to identify in vitro sensitivity of venetoclax in a broad panel of *KMT2A*-rearranged infant ALL cell lines, and demonstrated additivity or synergy of venetoclax in combination with conventional chemotherapeutic agents or hypomethylating agents. These findings provide support for the next COG upfront clinical trial, which proposes to determine the benefit of integrating venetoclax into the conventional chemotherapy backbone for infants with *KMT2A*-rearranged ALL.

The combination of azacitidine with venetoclax was selected for in vivo assessment to facilitate rapid clinical translation given that the AALL15P1 trial will establish a safe and tolerable dose of azacitidine in this patient population. The addition of venetoclax to azacitidine resulted in a significant extension to EFS in sensitive *KMT2A*-rearranged infant ALL xenografts treated at both high and low disease burden. Mechanistic insight of this combination has been explored in the setting of AML [40, 41]. Azacitidine and venetoclax synergize to efficiently and selectively target leukemia stem cells by disrupting energy metabolism via the tricarboxylic acid cycle, with reduced glutathionylation of succinate dehydrogenase leading to inhibition of the electron transport chain complex II and suppression of oxidative phosphorylation [40]. More recently, venetoclax was shown to enhance the effector activity of antileukemic T cells by increasing reactive oxygen species generation through inhibition of respiratory chain supercomplex formation, with azacitidine priming AML cells for killing by T cells by inducing a viral mimicry response through activation of the STING/cGAS pathway [41]. While further work is required to gain mechanistic insight of azacitidine and venetoclax for infants with *KMT2A*-rearranged ALL, this immune mediated mechanism would not be reflected by our experimental models due to the absence of T cells, thus there is the potential for our findings to be more pronounced in the setting of functional T cells.

In conclusion, we identify a significant survival benefit for single agent azacitidine and decitabine in *KMT2A*-rearranged infant ALL xenograft models providing support for clinical use of these agents in this patient population. The effect of hypomethylation following treatment led to changes in gene expression and TPP revealed the proteome-wide target landscape of these agents, including the binding of azacitidine to RNA methyltransferases and TYMS. The effect of azacitidine on in vivo survival was enhanced by the addition of venetoclax. The combination of azacitidine and venetoclax has been effectively used to treat patients with AML [11, 12], with disruption of the metabolic machinery driving energy metabolism leading to eradication of leukemia stem cells [40]. Given that a stem cell signature and myeloid features have been identified in *KMT2A*-rearranged infant ALL blasts [42, 43], cell stemness has been identified in relapse-initiating cells [44], and certain infants benefit from myeloid-style consolidation therapy [45], *KMT2A*-rearranged infant ALL represents an ideal disease model in which to further investigate the combination of azacitidine and venetoclax.

## Supplementary information

Supplementary Methods

Supplementary Figures

Supplementary Tables

## Data Availability

Raw methylation and transcriptomic data are available via the Gene Expression Omnibus (GEO) database under the accession number GSE212592. The TPP data were deposited to the ProteomeXchange Consortium via the PRIDE partner repository, identifier PXD030539 with reviewer account details: Username: reviewer_pxd030539@ebi.ac.uk and Password: bMPpON7w.
