# Supplementary material for: Preclinical efficacy of azacitidine and venetoclax for infant KMT2A-rearranged acute lymphoblastic leukemia reveals a new therapeutic strategy
Source: Leukemia. 2022 Nov 15;37(1):61–71. doi: 10.1038/s41375-022-01746-3 (PMC9883157; doi:10.1038/s41375-022-01746-3)
Supplement: Supplementary file 1 — Supplementary Methods [file 41375_2022_1746_MOESM1_ESM.docx]

**Supplemental Methods**

**Preclinical efficacy of azacitidine and venetoclax for infant *KMT2A*-rearranged ALL reveals a new therapeutic strategy**

Laurence C Cheung,^1,2,3^ Carlos Aya-Bonilla,^1,4^ Mark N Cruickshank,^4^ Sung K Chiu,^1^ Vincent Kuek,^1,2,4^ Denise Anderson,^1^ Grace-Alyssa Chua,^1^ Sajla Singh,^1^ Joyce Oommen,^1^ Emanuela Ferrari,^1^ Anastasia M Hughes,^1^ Jette Ford,^1^ Elena Kunold,^5^ Maria C Hesselman,^5^ Frederik Post,^5^ Kelly E Faulk,^6^ Erin H Breese,^7,8^ Erin M Guest,^9^ Patrick A Brown,^10^ Mignon L Loh,^11^ Richard B Lock,^12^ Ursula R Kees,^1,4^ Rozbeh Jafari,^5^ Sébastien Malinge,^1,4^ and Rishi S Kotecha.^1,2,4,13*^

^1^ Leukaemia Translational Research Laboratory, Telethon Kids Cancer Centre, Telethon Kids Institute, Perth, Western Australia, Australia

^2^ Curtin Medical School, Curtin University, Perth, Western Australia, Australia

^3^ Curtin Health Innovation Research Institute, Curtin University, Perth, Western Australia, Australia

^4^ The University of Western Australia, Perth, Western Australia, Australia

^5^ Department of Oncology-Pathology, Clinical Proteomics Mass Spectrometry, Karolinska Institutet, Science for Life Laboratory, Solna, Sweden

^6^ University of Colorado Anschutz Medical Campus, Children’s Hospital Colorado, Aurora, Colorado, USA

^7^ Cancer and Blood Diseases Institute, Division of Oncology, Cincinnati Children’s Hospital Medical Center, Cincinnati, Ohio, USA

^8^ Department of Pediatrics, University of Cincinnati College of Medicine, Cincinnati, Ohio, USA

^9^ Division of Hematology, Oncology, Blood and Marrow Transplantation, Children’s Mercy Kansas City, Kansas City, Missouri, USA

^10^ Division of Pediatric Oncology, Sidney Kimmel Comprehensive Cancer Centre, John Hopkins University, Baltimore, Maryland, USA

^11^ Division of Pediatric Hematology, Oncology, Bone Marrow Transplant and Cellular Therapy, Seattle Children’s Hospital, Seattle, Washington, USA

^12^ Children’s Cancer Institute, Lowy Cancer Research Centre/School of Women’s and Children’s Health/UNSW Centre for Childhood Cancer Research, UNSW Sydney, Kensington, New South Wales, Australia

^13^ Department of Clinical Haematology, Oncology, Blood and Marrow Transplantation, Perth Children’s Hospital, Perth, Western Australia, Australia

**Corresponding Author:**

*Dr Rishi S Kotecha

Department of Clinical Haematology, Oncology, Blood and Marrow Transplantation, Perth Children’s Hospital, Level 2D, 15 Hospital Avenue, Nedlands, Perth, Western Australia 6009, Telephone: +61 8 6456 4431, Fax: +61 8 6456 2360, Email: [rishi.kotecha@health.wa.gov.au](mailto:rishi.kotecha@health.wa.gov.au)

**Western blotting**

Six cell lines (PER-490, PER-703, PER-784, PER-785, PER-826 and PER-910) were incubated with 1.5µM azacitidine or 1.5µM decitabine for 72 hours at 37°C and protein extraction was performed. Samples were washed twice with cold phosphate buffered saline (PBS) and resuspended in 10µl of EVAN’s buffer (1mM EDTA, 1mM EGTA, 1% NP-40, 50mM Tris-HCl, 120nM NaCl) containing cOmplete™ protease inhibitor (Roche) for every one million cells. After 30 minutes of incubation at 4°C, samples were centrifuged at 13,000 rpm for 10 minutes, the supernatant was collected and quantified using the Direct Detect Spectrometer (Merck). Western blots were performed using the WES machine (ProteinSimple, Bio-Techne) according to the manufacturer’s instructions. Briefly, 2µg of total protein per samples were diluted in 0.1X sample buffer, combined with fluorescent marker and heated at 95°C for 5 minutes. Once heated, samples were kept on ice until they were ready to be loaded. Primary antibodies (Cell Signaling Technology) were diluted in antibody diluent (ProteinSimple) and used at a final dilution of 1:50 for DNMT1 and 1:100 for β-actin. Secondary antibodies (ProteinSimple) were bought ready to use. Samples, antibody diluent, primary antibodies, HRP-conjugated secondary antibodies, chemiluminescence substrate and wash buffer were loaded onto the plate according to the pre-designed layout. Data were analyzed using the Compass software (ProteinSimple), quantified and normalized to B actin according to the area under the peak.

**Apoptosis and necrosis assays**

Apoptosis and necrosis assays were performed using the RealTime-Glo™ Annexin V Apoptosis and Necrosis kit (Promega) according to the manufacturer’s instructions. In brief, cells were seeded into a 96-well plate with azacitidine, decitabine and venetoclax added at the half maximum inhibitory concentration (IC_50_) for each of the 14 *KMT2A*-rearranged infant ALL *in vitro* models and monitored for emission of luminescence and fluorescence every 30 minutes over 72 hours using a CLARIOstar multi-mode plate reader (BMG Labtech). Positive controls included dactinomycin at the IC_50_ for each cell line as an apoptosis inducing drug and 5% DMSO for induction of necrosis (data not shown). The data were analysed using MARS Data Analysis Software (BMG Labtech).

**Assessment of *in vivo* efficacy**

Female NOD/SCID mice were purchased from the Animal Research Centre, Perth. Animals were housed under pathogen free conditions and all studies were approved by the Animal Ethics Committee, Telethon Kids Institute, Perth (Ethics numbers #312 and #355). Eight-week old mice were inoculated with 1x10^6^ PER-785, 1x10^6^ MLL-5, 2x10^6^ MLL-7, 2x10^6^ MLL-14 or 2x10^6^ LR-iALL2 cells. The maximum tolerated doses (MTDs) of azacitidine and decitabine were determined in the PER-785 xenograft. Drug treatment was commenced when the percentage of human CD19^+^ CD45^+^ cells reached 1% in the bone marrow, identified from extensive mapping of leukemia cell kinetics.^1^ Following a 12-day engraftment period, mice were randomized into eight groups of five mice. Azacitidine was administered intravenously *via* tail vein injection once daily for five continuous days at 2.5mg/kg, 5.0mg/kg and 8.0mg/kg and compared to vehicle control. Decitabine was administered once daily for five continuous days *via* intraperitoneal injection at 0.3mg/kg, 0.4mg/kg and 0.5mg/kg and compared to vehicle control. Mice were euthanized one week from the start of therapy and leukemia burden was determined by measuring the percentage of human CD19^+^ CD45^+^ cells in the bone marrow and spleen by flow cytometry with anti-human CD19-APC and CD45-PE antibodies (BD Biosciences). Data were analyzed using the two tailed unpaired Student’s t-test.

All five xenograft models were used to determine the response to single agent drug treatment by event-free survival. For each xenograft model, mice were randomized into four groups of 8 to 10 mice, comprising of two azacitidine treatment groups at the MTD and dose level below the MTD, one decitabine treatment group at the MTD and vehicle control. Drug treatment was commenced when the percentage of human CD19^+^ CD45^+^ cells reached 1% in the bone marrow. The efficacy of azacitidine in combination with venetoclax was assessed in the PER-785 and LR-iALL2 xenografts. For each xenograft model, mice were randomized into five groups of 9 or 10 mice, and treatment commenced when the percentage of human CD19^+^ CD45^+^ cells reached 1% in the bone marrow. Treatment groups comprised of single agent azacitidine administered intravenously *via* tail vein injection once daily for five continuous days at the dose level below the MTD, single agent venetoclax at 100mg/kg via oral gavage for 21 days,^2^ azacitidine and venetoclax administered simultaneously, azacitidine followed by sequential administration of venetoclax and vehicle control. The experiment was also repeated in the setting of high disease burden, with drug treatment commencing when the percentage of human CD19^+^ CD45^+^ cells reached 1% in the peripheral blood. Individual mouse event-free survival was calculated as the time in days from treatment initiation until mice reached a humane end point with evidence of leukemia-related morbidity. *In vivo* survival studies were analyzed using log-rank test. Statistical analyses and graphics were performed using GraphPad Prism version 9.2.0. A *p*-value <0.05 was considered statistically significant.

**Methylation profiling**

Six cell lines (PER-490, PER-703, PER-784, PER-785, PER-826 and PER-910) were used for analysis of DNA methylation. One million cells per millilitre were seeded into each well of a 24-well cell culture plate (Nunclon, Thermo Fisher Scientific). For each cell line, hypomethylating drugs were added at a final concentration of 1.5µM azacitidine, 1.5µM decitabine, 6.0µM zebularine in comparison to untreated control. These concentrations were selected to reflect very low inhibitory concentration values of each drug, with the knowledge that DNA hypomethylation is more effective at low concentrations.^3^ Cells were incubated for 72 hours at 37°C and genomic DNA was extracted using the DNeasy Blood & Tissue Kit (Qiagen) according to manufacturer’s instructions. DNA was quantified using a NanoDrop 1000 Spectrophotometer (Thermo Fisher Scientific). DNA methylation assays were performed as per standard manufacturer’s instructions by the Australian Genome Research Facility. Briefly, samples were randomized for distribution across three bead chips. 750ng of DNA was treated with sodium bisulfite using the Zymo EZ DNA Methylation kit (Irvine). DNA was then hybridized to the Infinium MethylationEPIC BeadChip (Illumina). After washing off unhybridized DNA, a single base extension was recorded to calculate the methylation level at the CpG probe site.

**Differential methylation analysis**

Quality control checks and preprocessing were performed using the Bioconductor packages, lumi and minfi, and R (version 3.4.0).^4^ No samples were excluded based on mean detection *p*-values across the probes or due to gender discrepancies. Furthermore, multi-dimensional scaling plots did not reveal any outlying samples for exclusion. The data were quantile normalized and probes that were undetected in one or more samples were removed (n=5,007; detection *p-*value <0.01).^5^ Probes with SNPs at the CpG site, probes targeting sex chromosomes and cross-reactive probes were also removed.^6^ Following quality control checks, 773,929 probes were taken forward for downstream analyses. Differential methylation analysis of CpG sites was performed using *limma*, adjusting for pairing by cell line.^7^ Manhattan plots of the results were produced using the qqman package.^8^ Differential methylation analysis of regions was performed using *DMRcate* where CpGs were annotated and the *limma* model specified using the cpg.annotate() function.^9^ Significant CpGs with adjusted *p*-values less than 0.05 were taken forward to the dmrcate() function to identify differentially methylated regions using a Gaussian kernel bandwidth of 1,000 and a scaling factor of 2.^10^ Stouffer scores were used to rank differentially methylated regions. Significant hypomethylated regions were selected based on Stouffer *p*-value (<0.05), mean beta fold change (<-0.1) and five or more CpG sites per region.

**RNA sequencing**

Transcriptomic profiling was assessed in the same six cell lines and following the same experimental design as used for the methylation profiling. Three biological replicates were performed for all samples. Total RNA was extracted using the RNeasy Plus Mini Kit (Qiagen) as per manufacturer’s instructions. The yield and quality of the isolated RNA was assessed using a LabChip GX (PerkinElmer) with all samples displaying high RNA quality (RIN >7.5). After passing quality control, 500ng of purified total RNA per sample was used as input for library preparation using the Illumina Stranded mRNA Prep Kit (Illumina), as per manufacturer’s instructions by the Australian Genome Research Facility. A total of 54 poly A stranded libraries (six untreated controls, six azacitidine-treated and six decitabine-treated cell lines in triplicate) were sequenced to a depth of >20 million paired-end reads (150 bp) per sample using a NovaSeq 6000 (Illumina). Bioinformatic analysis was performed by the Australian Genome Research Facility following standardized pipelines, which involved demultiplexing (DRAGEN BCL Convert 07.021.609.3.9.3 pipeline), quality control (>87% bases above Q30 across all samples), alignment (STAR aligner version 2.7.10a, reference human genome build version hg38) and transcriptome assembly (StringTie tool version 2.1.4, Gencode annotation).

Differential expression analysis was performed using the edgeR (version 3.32.1) package and R (version 4.0.3).^4^ Raw counts were normalized using the trimmed mean of M-values (TMM) method. Gene expression profiles were compared to untreated control for each individual cell line following each treatment and also after merging all cell lines following each treatment, accounting for 14 paired analyses. Differentially expressed genes were defined as genes with a log_2_ fold change lower than -1 (downregulated) and higher than 1 (upregulated) with false discovery rate adjusted *p*-values less than 0.05. Volcano plots were created using the EnhancedVolcano package (version 1.14) and R (version 4.2.1).^4, 11^

Gene set enrichment analysis (GSEA) was performed on ranked differentially expressed gene lists which interrogated enrichment of gene sets within annotated hallmark (H) and curated gene sets (C2) collections available through the Molecular Signatures Database (MSigDB version 7.5.1).^12^ Of the curated gene sets (C2) collections, interrogated gene sets included chemical and genetic perturbations (CGP) and canonical pathways, containing KEGG, Reactome, WikiPathways and BioCarta databases. GSEA calculated normalized enrichment scores (NES) across all gene-sets, for each sample, using the GSEA() function within the clusterProfiler package (version 4.4.4) and R (version 4.2.1),^4, 13^ with 10,000 permutations and a false discovery rate cut off of <0.1 as parameters.

**Targeted gene expression analysis**

RNA was extracted from an extended cohort of 13 *KMT2A*-rearranged infant ALL *in vitro* models (PER-490, PER-494, PER-703, PER-784, PER-785, PER-826, PER-910, ALL-PO, KOPN-8, MLL-5, MLL-7, MLL-14, and LR-iALL2), placed under the same conditions as for the methylation and transcriptomic profiling, using the RNeasy Plus Mini Kit (Qiagen) according to manufacturer’s instructions. Total RNA was quantified using the Qubit™ RNA BR Assay Kit on a Qubit 4 fluorometer (Thermo Fisher Scientific). cDNA was synthesized from 1µg of total RNA using SuperScript™ IV VILO™ Master Mix (Thermo Fisher Scientific) according to manufacturer’s instructions. TaqMan assays (Thermo Fisher Scientific) were used to determine gene expression for *MMP15* (Hs00233997_m1), *CD82* (Hs01017982_m1), *BAIAP3* (Hs00187529_m1) and *GAPDH* (Hs02786624_g1) as a housekeeping gene.

To determine gene expression for the ATP-binding cassette transporters, three cell lines which demonstrated antagonism between methotrexate and the hypomethylating agents were treated with azacitidine (40µM for PER-485 and PER-826; 20µM for PER-490) or decitabine (20µM for PER-490 and PER-826; 50µM for PER-485) for 72 hours. Total RNA was extracted, quantified and cDNA synthesized as previously described. TaqMan assays were used to determine gene expression for *ABCB1* (Hs00184500_m1), *ABCB4* (Hs00983957_m1), *ABCC1* (Hs01561483_m1), *ABCC2* (Hs00960489_m1), *ABCC3* (Hs00978452_m1), *ABCG2* (Hs01053790_m1) and *GAPDH* (Hs02786624_g1) as a housekeeping gene.

TaqMan assays were performed in three biological replicates for all samples. For each sample, a mastermix containing 1μg of cDNA, 1X TaqMan Fast Advanced Master Mix (Thermo Fisher Scientific) and 1X TaqMan assay, in a final volume of 10μl was subjected to the following cycling conditions: 50°C for 2 minutes and 95°C for 2 minutes, followed by 40 cycles of 95°C for 1 second and 60°C for 20 seconds in a QuantStudio™ 7 Flex System (Thermo Fisher Scientific). Relative gene expression quantification was performed using the 2^-ΔΔ^*^CT^* method.^14^ The unpaired t test was used to compare expression levels between untreated and treated samples. A *p*-value <0.05 was considered statistically significant.

**Thermal proteome profiling**

The ALL-PO cell line was obtained from Banca Biologica e Cell Factory (San Martino, Italy). ALL-PO cells were cultured in Roswell Park Memorial Institute (RPMI) 1640 (AQmedia, Sigma-Aldrich) containing 2mM stable glutamine (L-Ala-L-Gln dipeptide) and supplemented with 10% fetal bovine serum (FBS, Sigma-Aldrich), 20mM HEPES (Gibco/Life Technologies), 1mM sodium pyruvate (Sigma-Aldrich), 1x MEM non-essential amino acids (Sigma-Aldrich), and 1x Penicillin-Streptomycin (Sigma-Aldrich). ALL-PO cells were grown at 37°C and 5% CO_2_ to a cell density of approximately 1-2 million cells/ml (NucleoCounter® NC-200™, ChemoMetec). Cell were distributed into six different T25 flasks to a density of 8x10^6^ cells/ml in 6ml medium. They were incubated with 100μM azacitidine, decitabine or DMSO at a final DMSO concentration of 0.2% for 2 hours at 37°C and 5% CO_2_. Two replicates for each drug and DMSO treatment were performed. Cells were pelleted at 300 x *g* and room temperature for 3 minutes and washed 2x with Hank’s balanced salt solution (Thermo Fisher Scientific). Cells were resuspended to a density of ~70x10^6^ cells/ml and distributed as 65μl aliquots into 0.2ml PCR tubes (Thermo Scientific/Life Technologies) and one of each of the compounds and DMSO containing tubes was heated for 3 minutes in a Veriti Thermal Cycler (Applied Biosystems/Thermo Scientific) to the respective ten temperatures (37, 41, 44, 47, 50, 53, 56, 59, 63, 67°C), followed by a 3 minute incubation at room temperature. Afterwards, cells were flash-frozen in liquid nitrogen. Cells were thawed at 25°C and lysed by this freeze-thawing cycle, repeated another three times. Cell debris and precipitated proteins were removed by centrifugation at 21,000 x *g* for 30 minutes at 4°C. Supernatants were transferred to new tubes and protein concentrations were determined (DC protein assay, Bio-Rad). Equal volumes of each condition that correspond to 120μg protein in the 37°C sample were transferred to new tubes and subjected to the following digestion. First, the samples were diluted to contain 50mM TEAB, 0.1% SDS and 5mM TCEP. Reduction was performed at 65°C for 30 minutes. The samples were then cooled down to room temperature and alkylated with 15mM of chloroacetamide for 30 minutes. The proteins were digested overnight with 1-40 Lys-C (Wako Chemicals GmbH) to protein-ratio and consecutively with Trypsin (Thermo Fisher Scientific) at a 1:25 enzyme to protein ratio. The digested peptides were labelled by 10-plex TMT-tags (TMT10, Thermo Fisher Scientific) using 0.6mg of the respective label for each sample. The labelling efficiency was determined by liquid chromatography tandem mass spectrometry (LC-MS/MS) before pooling of the samples. An aliquot of 10µg was suspended in LC mobile phase A (3% acetonitrile in 0.1% formic acid) and 1µg was injected on the LC-MS/MS system. Fully labelled peptide extracts were combined to a single pooled sample per experiment (azacitidine, decitabine, DMSO). For the sample clean-up step, a solid phase extraction (SPE strata-X-C, Phenomenex, Torrance, CA, USA) was performed and purified samples were dried in a Savant SpeedVac™ vacuum centrifuge (Thermo Fisher Scientific) and 300μg of pooled, cleaned peptides were pre-fractionated by means of high pH reversed-phase chromatography (XBridge BEH C18 (2.1mmx250 mm) 300A, 3.5μm, Waters Corporation) using a 63 minute gradient from 3% to 80% acetonitrile in 20mM ammonia. Collected fractions were pooled into 20 samples, dried in Savant SpeedVac™ vacuum centrifuge (Thermo Fisher Scientific) and subjected to LC-MS/MS measurements.

**LC-MS/MS runs**

Online LC-MS/MS was performed using a Dionex UltiMate™ 3000 RSLCnano System coupled to a Q-Exactive-HF mass spectrometer (Thermo Fisher Scientific). Each of the samples was dissolved in 20μl solvent A and 10μl were injected. Samples were trapped on a C18 guard-desalting column (Acclaim PepMap 100, 75μmx2cm, nanoViper, C18, 5µm, 100Å, Thermo Fisher Scientific), and separated on a 50cm long C18 column (Easy spray PepMap RSLC, C18, 2μm, 100Å, 75μmx50cm, Thermo Fisher Scientific). The nano capillary solvent A was 95% water, 5% DMSO, 0.1% FA; and solvent B was 5% water, 5% DMSO, 95% acetonitrile, 0.1% FA. At a constant flow of 0.25μlmin^−1^, a curved gradient from 3-8% B (in 2 minutes, curve of 4) and from 8%-45% B (in 148 minutes, curve of 5) was used followed by a steep increase to 99% B in 2 minutes. FTMS master scans were performed in a mass range of 3000-1500m/z applying a resolution of 60,000 (mass range 300-1500m/z), followed by data-dependent MS/MS (35,000 resolution) on the top 5 ions using higher energy collision dissociation at 30% normalized collision energy. Precursors were isolated with a 2m/z window and 0.5m/z isolation offset. Automatic gain control targets were 1e^6^ for MS1 and 1e^5^ for MS2. Maximum injection times were 100ms for MS1 and 100ms for MS2. The entire duty cycle lasted ~2.5 seconds. Dynamic exclusion was used with 30 second duration. Precursors with unassigned charge state or charge state 1 were excluded. An underfill ratio of 1% was used.

**Thermal proteome profiling MS data processing**

For processing, quantification, and normalization the IsobarQuant (1.1.0) package was used. Peptide and protein identification were performed using Mascot 2.4 (Matrix Science) search engine. The searches were done against a human database from Uniprot including known contaminants and the reversed protein sequences. Search parameters included trypsin as digestion enzyme, three allowed missed cleavages, a precursor tolerance of 10ppm at 0.02 Da for MS/MS tolerance. As fixed modifications, carbamidomethylation on cysteines and TMT10plex on lysines were included. Variable modifications were acetylation on protein N termini, oxidation of methionines and TMT10plex on peptide N-termini. The determination of melt curves and significant aggregation temperature shifts from the quantitative protein data of the raw output data from IsobarQuant was then performed with the “NPARC” and “TPP” packages.^15, 16^

**CETSA temperature range and dose response time course in cells**

For CETSA temperature range, ALL-PO cells were cultured as previously described, azacitidine was added to a final concentration of 100µM and DMSO was added as vehicle to control samples and incubated for 3 hours at 37°C and 5% CO_2_. Cell suspensions were then centrifuged at 300 x *g* for 5 minutes, the supernatant was discarded, and the cells were washed twice with Hank’s Balanced Salt Solution (HBSS, Gibco/Life Technologies). Pelleted cells were resuspended in HBSS and cell suspensions were aliquoted to 0.2ml tubes. Samples were then heated to a temperature range of 37-70°C in a Veriti Thermal Cycler (Applied Biosystems/Thermo Fisher Scientific) for 3 minutes, followed by 3 minutes cooling at room temperature and immediate snap-freezing in liquid nitrogen. The cells were then lysed by three repeated freeze-thaws and centrifuged at 21,000 x *g* for 30 minutes at 4°C. The cleared supernatants were transferred to new tubes, denatured in LDS sample buffer (Thermo Fisher Scientific) and analyzed by western blotting.

For CETSA dose response, ALL-PO cells were cultured as previously described, azacitidine was added to final concentrations of 100, 50, 25, 12.5, 6.25, 3.125, 1.56µM and DMSO in 100µl aliquots of 10x10^6^ cells/ml. Each concentration series was then incubated for 10, 20, 30, 60, 90, 120, 150 and 180 minutes respectively at 37°C and 5% CO_2_. Cells from each time point were then heated at a constant temperature of 48°C for 3 minutes in a Veriti Thermal Cycler (Applied Biosystems/Thermo Scientific) followed by 3 minutes of cooling at room temperature and immediate snap-freezing in liquid nitrogen. The cells were then lysed by three repeated freeze-thaw cycles and centrifuged at 21,000 x *g* for 30 minutes at 4°C. The cleared supernatants were transferred to new tubes, denatured in LDS sample buffer (Thermo Fisher Scientific) and analyzed by western blotting.

For CETSA dose response of BCL-2, ALL-PO cells were cultured as previously described, venetoclax was added to final concentrations of 1, 0.25, 0.0625, 0.156, 0.0001µM and DMSO in 100µl aliquots of 10x10^6^ cells/ml respectively and incubated at 37°C and 5% CO_2_ for 3 hours. Two replica experiments were performed. Cells were then heated at a constant temperature of 60°C for 3 minutes in a Veriti Thermal Cycler (Applied Biosystems/Thermo Fisher Scientific) followed by 3 minutes of cooling at room temperature and immediate snap-freezing in liquid nitrogen. The cells were then lysed by three repeated freeze-thaw cycles and centrifuged at 21,000 x *g* for 30 minutes at 4°C. The cleared supernatants were transferred to new tubes, denatured in LDS sample buffer (Thermo Fisher Scientific) and analyzed by western blotting.

Proteins in the cleared supernatants were denatured in SDS sample buffer (Thermo Fisher Scientific), resolved by SDS-PAGE using NuPAGE™ 4 to 12%, Bis-Tris Gel (Invitrogen™, Thermo Fisher Scientific), and transferred to Nitrocellulose membranes (Invitrogen™, Thermo Fisher Scientific). Afterwards, the membranes were blocked with 5% non-fat dry milk in TBST (Thermo Fisher Scientific) and incubated with primary antibodies. The following primary antibodies were used: NSUN2 Recombinant Rabbit Monoclonal Antibody (3H24L11) (#702036, Thermo Fisher Scientific), TYMS rabbit monoclonal antibody (D5B3) (#9045S, Cell Signaling Technology), BCL-2 (C-2) (sc-7382, Santa Cruz Biotech) and as loading controls, SOD-1 (G-11) (sc-17767, Santa Cruz Biotech) and actin (I-19) (sc-1616-R, Santa Cruz Biotech). Secondary antibodies were purchased from Santa Cruz Biotech. Protein bands were developed with Clarity ECL Substrate Chemiluminescent HRP substrate (Bio-Rad) in a ChemiDoc MP System (BioRad). Quantification was performed using Image Lab software version 5.2.1 (Bio-Rad).

**References for the Supplementary Methods**

1. Cheung LC, Cruickshank MN, Hughes AM, Singh S, Chua GA, Ford J*, et al.* Romidepsin enhances the efficacy of cytarabine in vivo, revealing histone deacetylase inhibition as a promising therapeutic strategy for KMT2A-rearranged infant acute lymphoblastic leukemia. *Haematologica* 2019; **104**(7)**:** e300-e303.

2. Khaw SL, Suryani S, Evans K, Richmond J, Robbins A, Kurmasheva RT*, et al.* Venetoclax responses of pediatric ALL xenografts reveal sensitivity of MLL-rearranged leukemia. *Blood* 2016; **128**(10)**:** 1382-1395.

3. Sato T, Issa JJ, Kropf P. DNA hypomethylating drugs in cancer therapy. *Cold Spring Harb Perspect Med* 2017; **7**(5):a026948.

4. R Core Team. R: a language and environment for statistical computing. *R Foundation for Statistical Computing, Vienna, Austria***:** 2021.

5. Bolstad BM, Irizarry RA, Astrand M, Speed TP. A comparison of normalization methods for high density oligonucleotide array data based on variance and bias. *Bioinformatics* 2003; **19**(2)**:** 185-193.

6. Chen YA, Lemire M, Choufani S, Butcher DT, Grafodatskaya D, Zanke BW*, et al.* Discovery of cross-reactive probes and polymorphic CpGs in the Illumina Infinium HumanMethylation450 microarray. *Epigenetics* 2013; **8**(2)**:** 203-209.

7. Ritchie ME, Phipson B, Wu D, Hu Y, Law CW, Shi W*, et al.* limma powers differential expression analyses for RNA-sequencing and microarray studies. *Nucleic Acids Res* 2015; **43**(7)**:** e47.

8. Turner SD. qqman: an R package for visualizing GWAS results using Q-Q and manhattan plots. *Journal of Open Source Software* 2018; **3**(25)**:** 731.

9. Peters TJ, Buckley MJ, Statham AL, Pidsley R, Samaras K, R VL*, et al.* De novo identification of differentially methylated regions in the human genome. *Epigenetics Chromatin* 2015; **8:** 6.

10. Benjamini Y, Hochberg Y. Controlling the false discovery rate: a practical and powerful approach to multiple testing. *Journal of the Royal Statistical Society Series B (Methodological)* 1995; **57**(1)**:** 289-300.

11. Blighe K, Rana S, Lewis M. EnhancedVolcano: Publication-ready volcano plots with enhanced colouring and labeling. 2022. <https://github.com/kevinblighe/EnhancedVolcano>.

12. Liberzon A, Birger C, Thorvaldsdóttir H, Ghandi M, Mesirov Jill P, Tamayo P. The molecular signatures database hallmark gene set collection. *Cell Systems* 2015; **1**(6)**:** 417-425.

13. Wu T, Hu E, Xu S, Chen M, Guo P, Dai Z*, et al.* clusterProfiler 4.0: A universal enrichment tool for interpreting omics data. *Innovation (Camb)* 2021; **2**(3)**:** 100141.

14. Livak KJ, Schmittgen TD. Analysis of relative gene expression data using real-time quantitative PCR and the 2(-Delta Delta C(T)) Method. *Methods* 2001; **25**(4)**:** 402-408.

15. Childs D, Bach K, Franken H, Anders S, Kurzawa N, Bantscheff M*, et al.* Nonparametric analysis of thermal proteome profiles reveals novel drug-binding proteins. *Mol Cell Proteomics* 2019; **18**(12)**:** 2506-2515.

16. Franken H, Mathieson T, Childs D, Sweetman GM, Werner T, Togel I*, et al.* Thermal proteome profiling for unbiased identification of direct and indirect drug targets using multiplexed quantitative mass spectrometry. *Nat Protoc* 2015; **10**(10)**:** 1567-1593.
