# Supplementary material for: Preclinical efficacy of azacitidine and venetoclax for infant KMT2A-rearranged acute lymphoblastic leukemia reveals a new therapeutic strategy
Source: Leukemia. 2022 Nov 15;37(1):61–71. doi: 10.1038/s41375-022-01746-3 (PMC9883157; doi:10.1038/s41375-022-01746-3)
Supplement: Supplementary file 2 — Supplementary Figures [file 41375_2022_1746_MOESM2_ESM.pdf]

Supplemental Figure S1. Azacitidine induces apoptosis and necrosis in *KMT2A*-rearranged infant acute lymphoblastic leukemia cell lines. Cells were incubated with IC_{50} concentrations of azacitidine for 72 hours. Measurements of luminescence (apoptosis) and fluorescence (necrosis) were performed every 30 minutes.

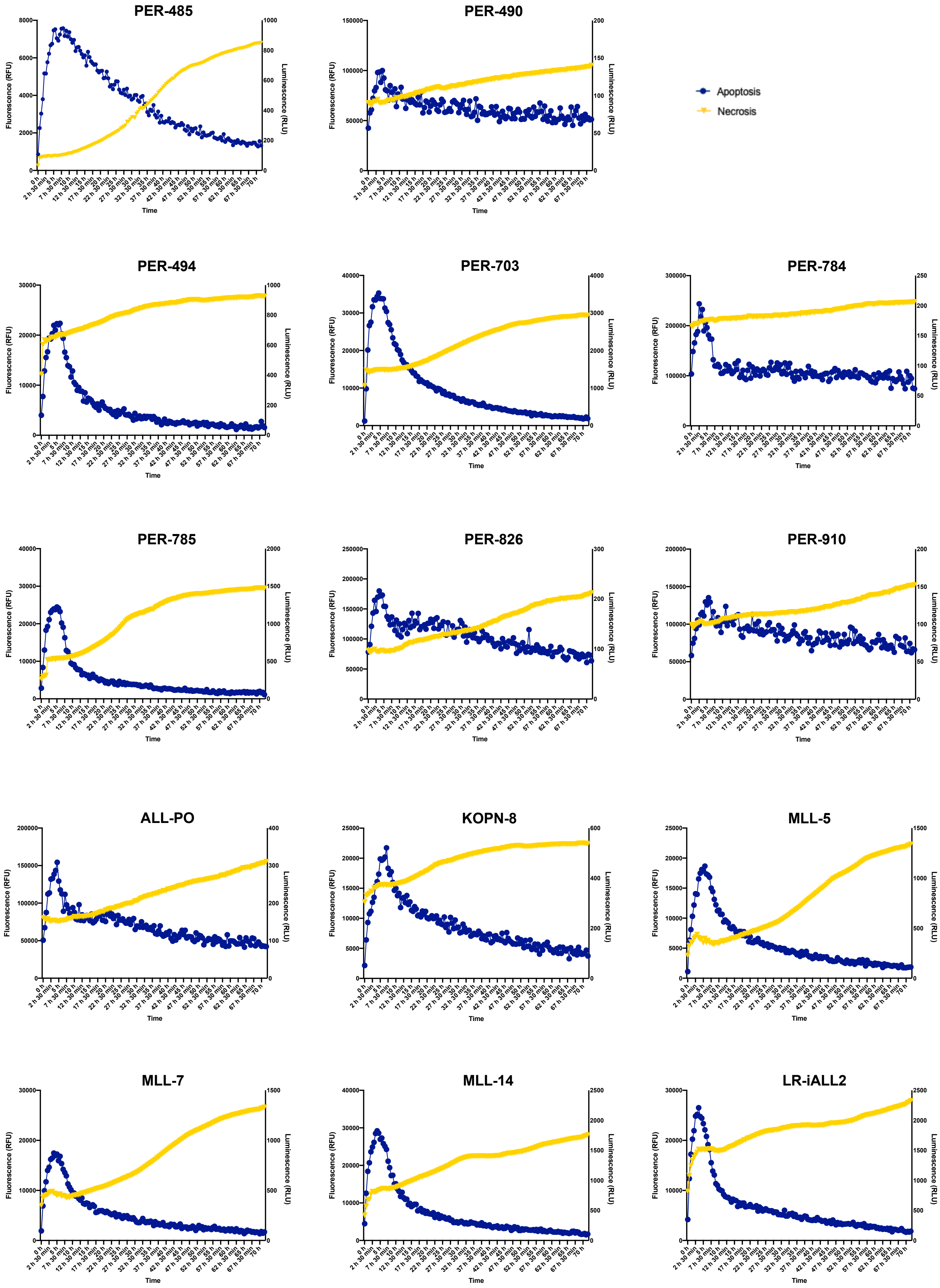

Supplemental Figure S2. Decitabine induces apoptosis and necrosis in *KMT2A*-rearranged infant acute lymphoblastic leukemia cell lines. Cells were incubated with IC_{50} concentrations of decitabine for 72 hours. Measurements of luminescence (apoptosis) and fluorescence (necrosis) were performed every 30 minutes.

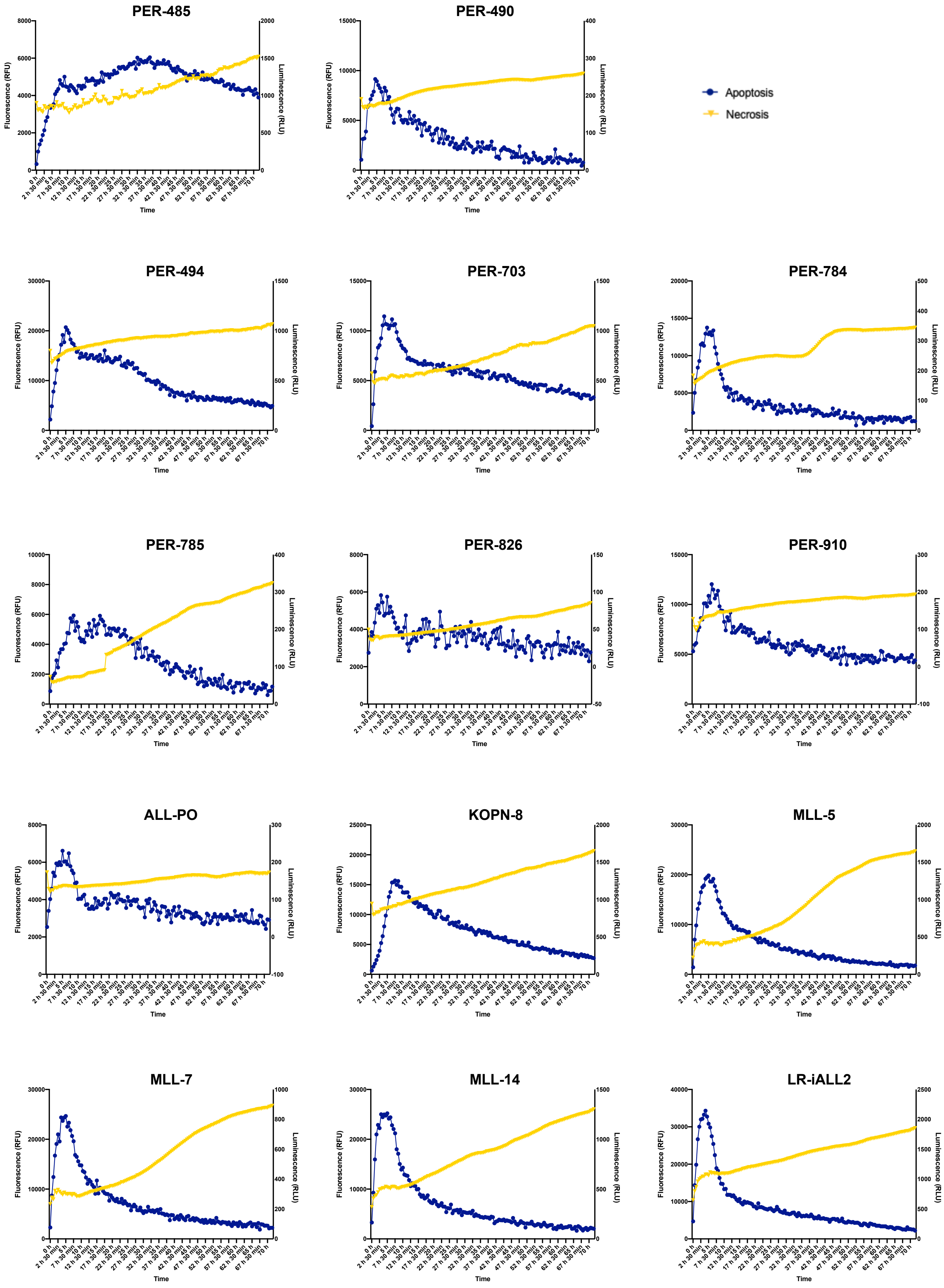

Supplemental Figure S3. Azacitidine and decitabine reduce expression of DNMT1 in *KMT2A*-rearranged infant acute lymphoblastic leukemia cell lines. Leukemia cell lines were treated with 1.5µM of azacitidine (AZA) or decitabine (DEC) for 72 hours before performing protein extraction. (A) Western blot against DNMT1 (~180kDa) and B actin (~47kDa). (B) Quantification of DNMT1 expression by densitometry from three independent experiments. The expression of DNMT1 is normalized to untreated control. *p<0.05, **p<0.01, ***p<0.001, ****p<0.0001.

A

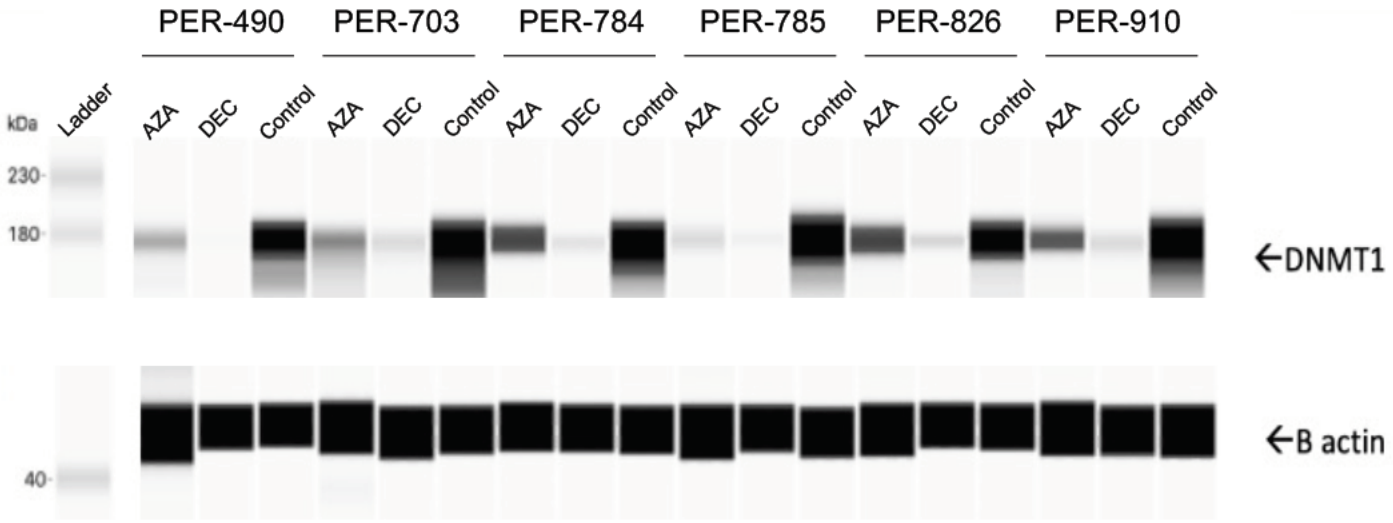

B

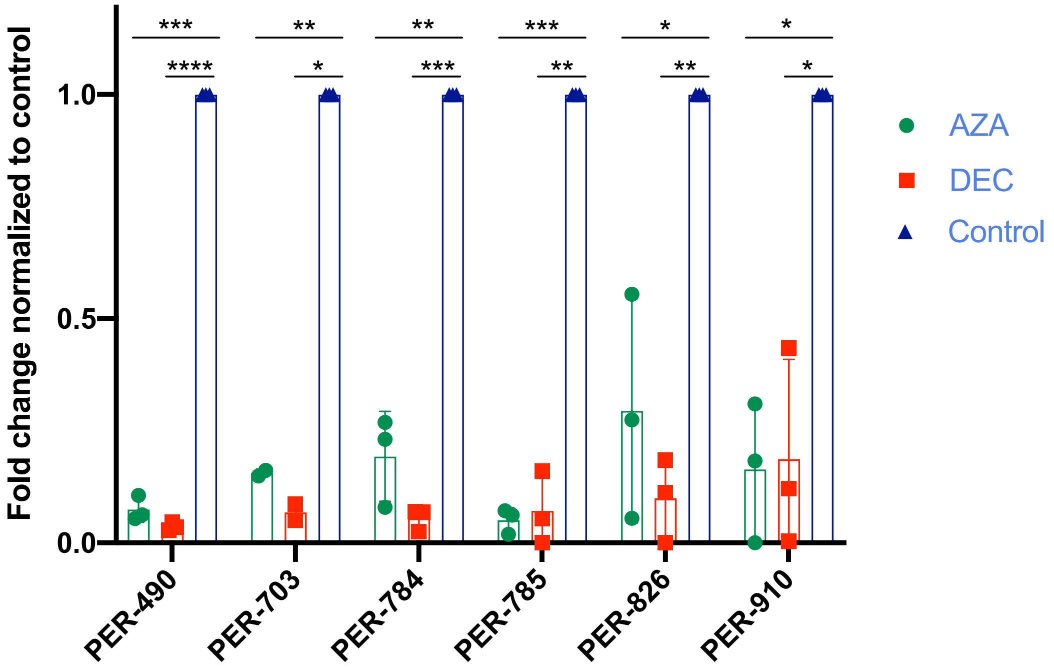

Supplemental Figure S4. The combinatorial dose response of hypomethylating agents with conventional chemotherapeutic agents compared to single agent on cell survival. Conventional chemotherapeutic agents include vincristine, daunorubicin, dexamethasone, cytarabine, methotrexate, 6-mercaptopurine, L-asparaginase, thioguanine and 4-hydroperoxycyclophosphamide (4-HPC). (A) Azacitidine with conventional chemotherapeutic agents. (B) Decitabine with conventional chemotherapeutic agents. (C) Zebularine with conventional chemotherapeutic agents.

A

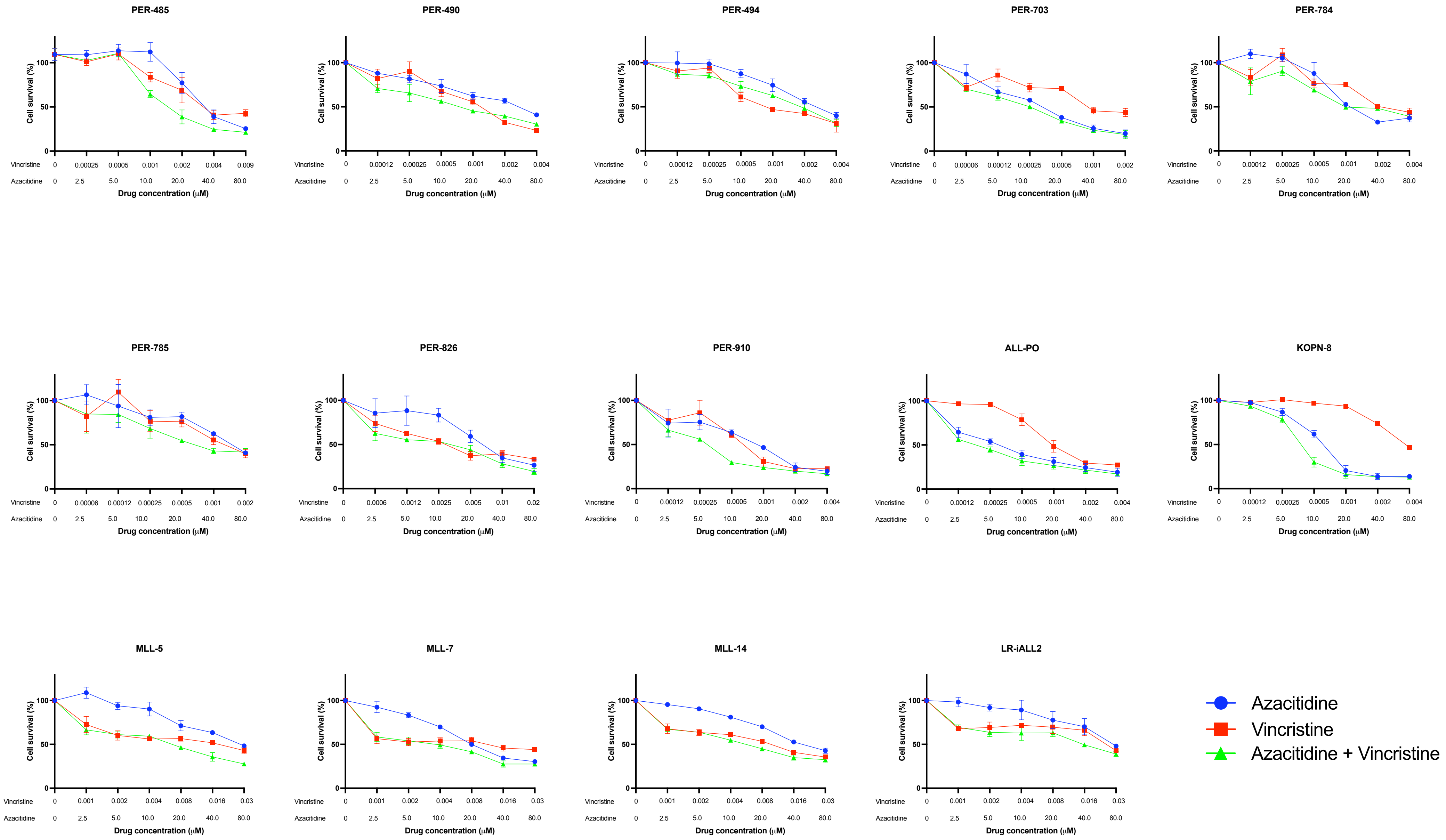

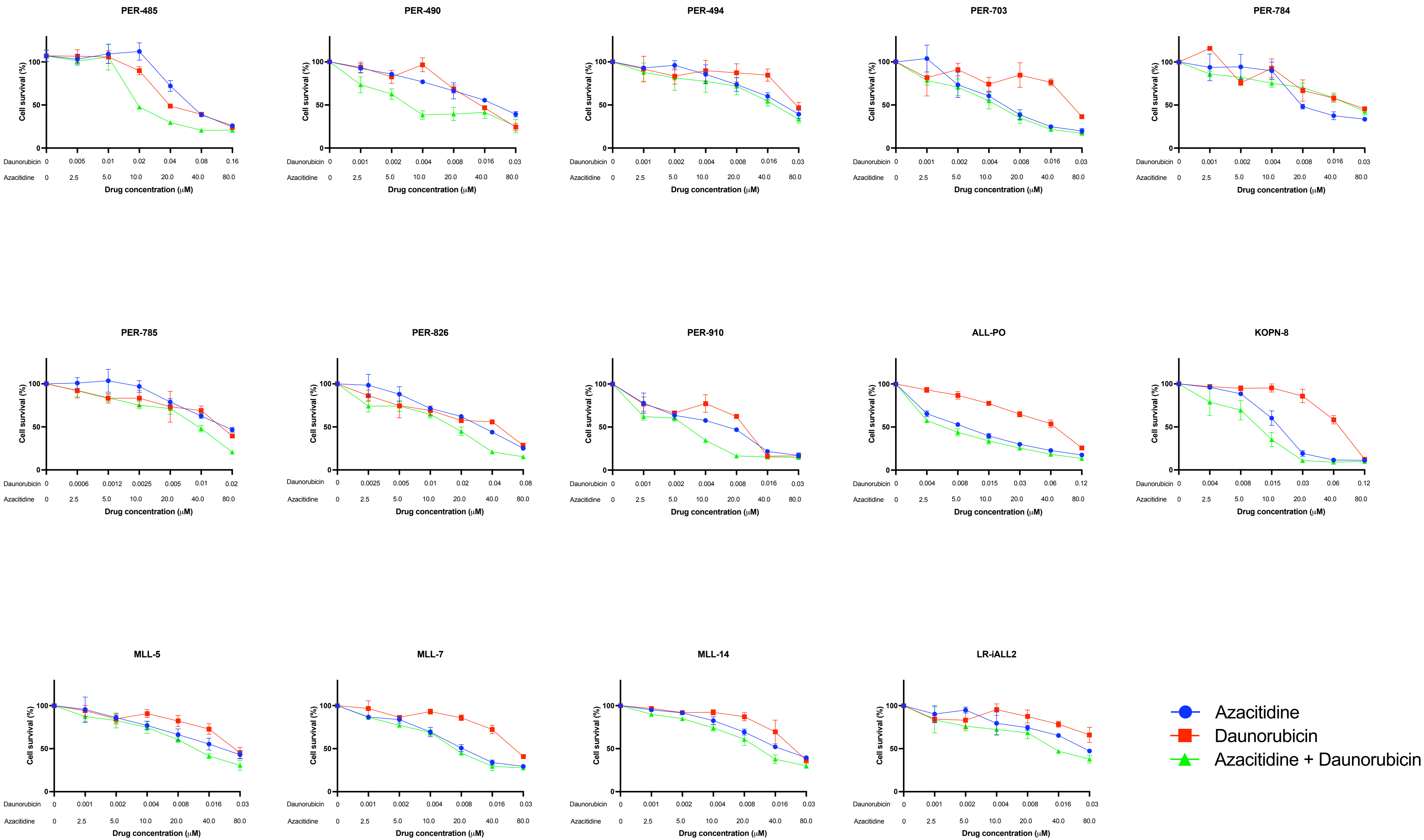

PER-485
Cell survival (%)
Daunorubicin 0 0.005 0.01 0.02 0.04 0.08 0.16
Azacitidine 0 2.5 5.0 10.0 20.0 40.0 80.0
Drug concentration (μM)
PER-490
Daunorubicin 0 0.001 0.002 0.004 0.008 0.016 0.03
Azacitidine 0 2.5 5.0 10.0 20.0 40.0 80.0
PER-494
Daunorubicin 0 0.001 0.002 0.004 0.008 0.016 0.03
Azacitidine 0 2.5 5.0 10.0 20.0 40.0 80.0
PER-703
Daunorubicin 0 0.001 0.002 0.004 0.008 0.016 0.03
Azacitidine 0 2.5 5.0 10.0 20.0 40.0 80.0
PER-784
Daunorubicin 0 0.001 0.002 0.004 0.008 0.016 0.03
Azacitidine 0 2.5 5.0 10.0 20.0 40.0 80.0
PER-785
Daunorubicin 0 0.0006 0.0012 0.0025 0.005 0.01 0.02
Azacitidine 0 2.5 5.0 10.0 20.0 40.0 80.0
PER-826
Daunorubicin 0 0.0025 0.005 0.01 0.02 0.04 0.08
Azacitidine 0 2.5 5.0 10.0 20.0 40.0 80.0
PER-910
Daunorubicin 0 0.001 0.002 0.004 0.008 0.016 0.03
Azacitidine 0 2.5 5.0 10.0 20.0 40.0 80.0
ALL-PO
Daunorubicin 0 0.004 0.008 0.015 0.03 0.06 0.12
Azacitidine 0 2.5 5.0 10.0 20.0 40.0 80.0
KOPN-8
Daunorubicin 0 0.004 0.008 0.015 0.03 0.06 0.12
Azacitidine 0 2.5 5.0 10.0 20.0 40.0 80.0
MLL-5
Daunorubicin 0 0.001 0.002 0.004 0.008 0.016 0.03
Azacitidine 0 2.5 5.0 10.0 20.0 40.0 80.0
MLL-7
Daunorubicin 0 0.001 0.002 0.004 0.008 0.016 0.03
Azacitidine 0 2.5 5.0 10.0 20.0 40.0 80.0
MLL-14
Daunorubicin 0 0.001 0.002 0.004 0.008 0.016 0.03
Azacitidine 0 2.5 5.0 10.0 20.0 40.0 80.0
LR-iALL2
Daunorubicin 0 0.001 0.002 0.004 0.008 0.016 0.03
Azacitidine 0 2.5 5.0 10.0 20.0 40.0 80.0
Azacitidine
Daunorubicin
Azacitidine + Daunorubicin

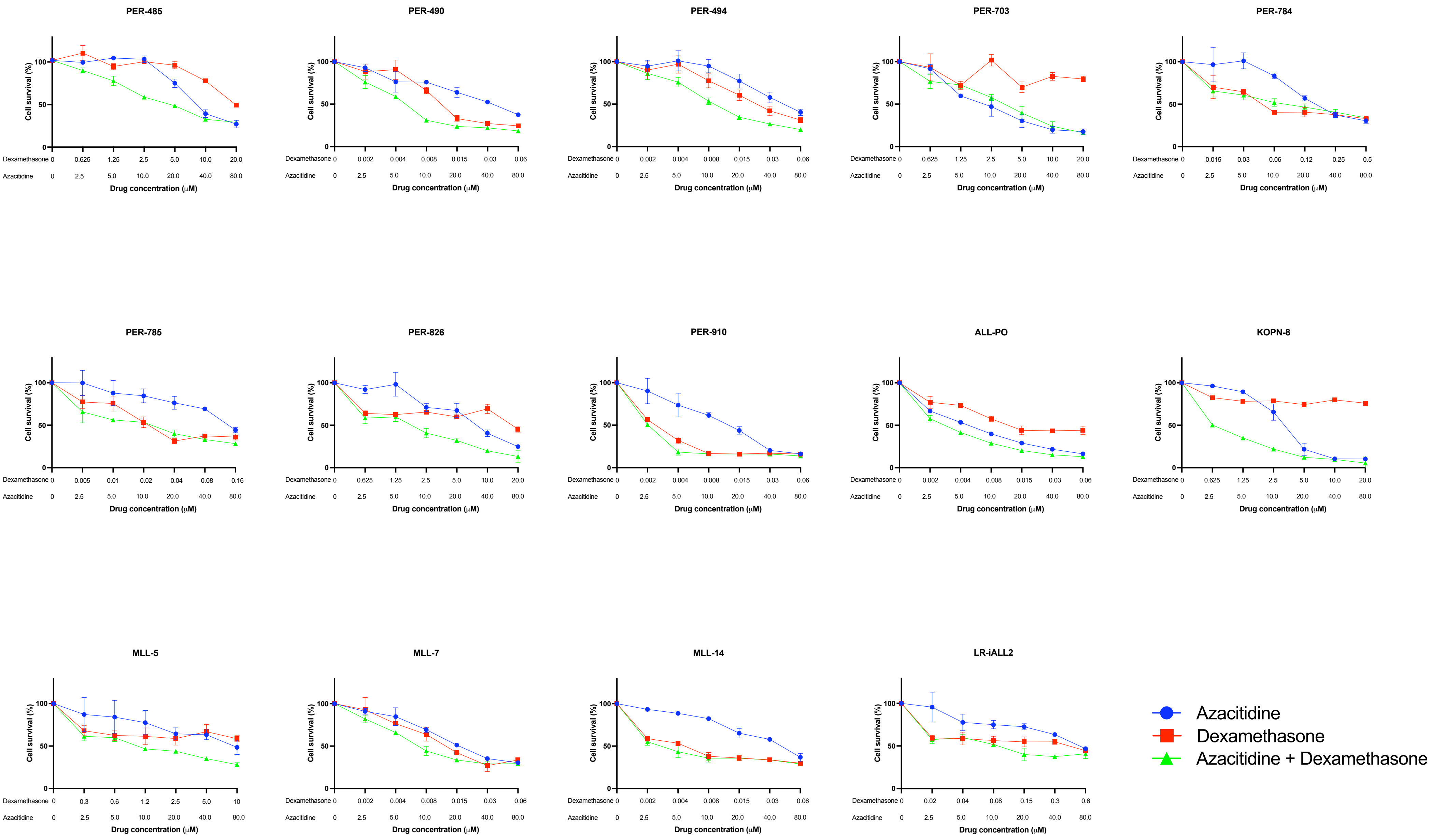
PER-485
Cell survival (%)
0
50
100
Dexamethasone 0 0.625 1.25 2.5 5.0 10.0 20.0
Azacitidine 0 2.5 5.0 10.0 20.0 40.0 80.0
Drug concentration (µM)
PER-490
Dexamethasone 0 0.002 0.004 0.008 0.015 0.03 0.06
Azacitidine 0 2.5 5.0 10.0 20.0 40.0 80.0
PER-494
Dexamethasone 0 0.002 0.004 0.008 0.015 0.03 0.06
Azacitidine 0 2.5 5.0 10.0 20.0 40.0 80.0
PER-703
Dexamethasone 0 0.625 1.25 2.5 5.0 10.0 20.0
Azacitidine 0 2.5 5.0 10.0 20.0 40.0 80.0
PER-784
Dexamethasone 0 0.015 0.03 0.06 0.12 0.25 0.5
Azacitidine 0 2.5 5.0 10.0 20.0 40.0 80.0
PER-785
Dexamethasone 0 0.005 0.01 0.02 0.04 0.08 0.16
Azacitidine 0 2.5 5.0 10.0 20.0 40.0 80.0
PER-826
Dexamethasone 0 0.625 1.25 2.5 5.0 10.0 20.0
Azacitidine 0 2.5 5.0 10.0 20.0 40.0 80.0
PER-910
Dexamethasone 0 0.002 0.004 0.008 0.015 0.03 0.06
Azacitidine 0 2.5 5.0 10.0 20.0 40.0 80.0
ALL-PO
Dexamethasone 0 0.002 0.004 0.008 0.015 0.03 0.06
Azacitidine 0 2.5 5.0 10.0 20.0 40.0 80.0
KOPN-8
Dexamethasone 0 0.625 1.25 2.5 5.0 10.0 20.0
Azacitidine 0 2.5 5.0 10.0 20.0 40.0 80.0
MLL-5
Dexamethasone 0 0.3 0.6 1.2 2.5 5.0 10
Azacitidine 0 2.5 5.0 10.0 20.0 40.0 80.0
MLL-7
Dexamethasone 0 0.002 0.004 0.008 0.015 0.03 0.06
Azacitidine 0 2.5 5.0 10.0 20.0 40.0 80.0
MLL-14
Dexamethasone 0 0.002 0.004 0.008 0.015 0.03 0.06
Azacitidine 0 2.5 5.0 10.0 20.0 40.0 80.0
LR-iALL2
Dexamethasone 0 0.02 0.04 0.08 0.15 0.3 0.6
Azacitidine 0 2.5 5.0 10.0 20.0 40.0 80.0
Azacitidine
Dexamethasone
Azacitidine + Dexamethasone

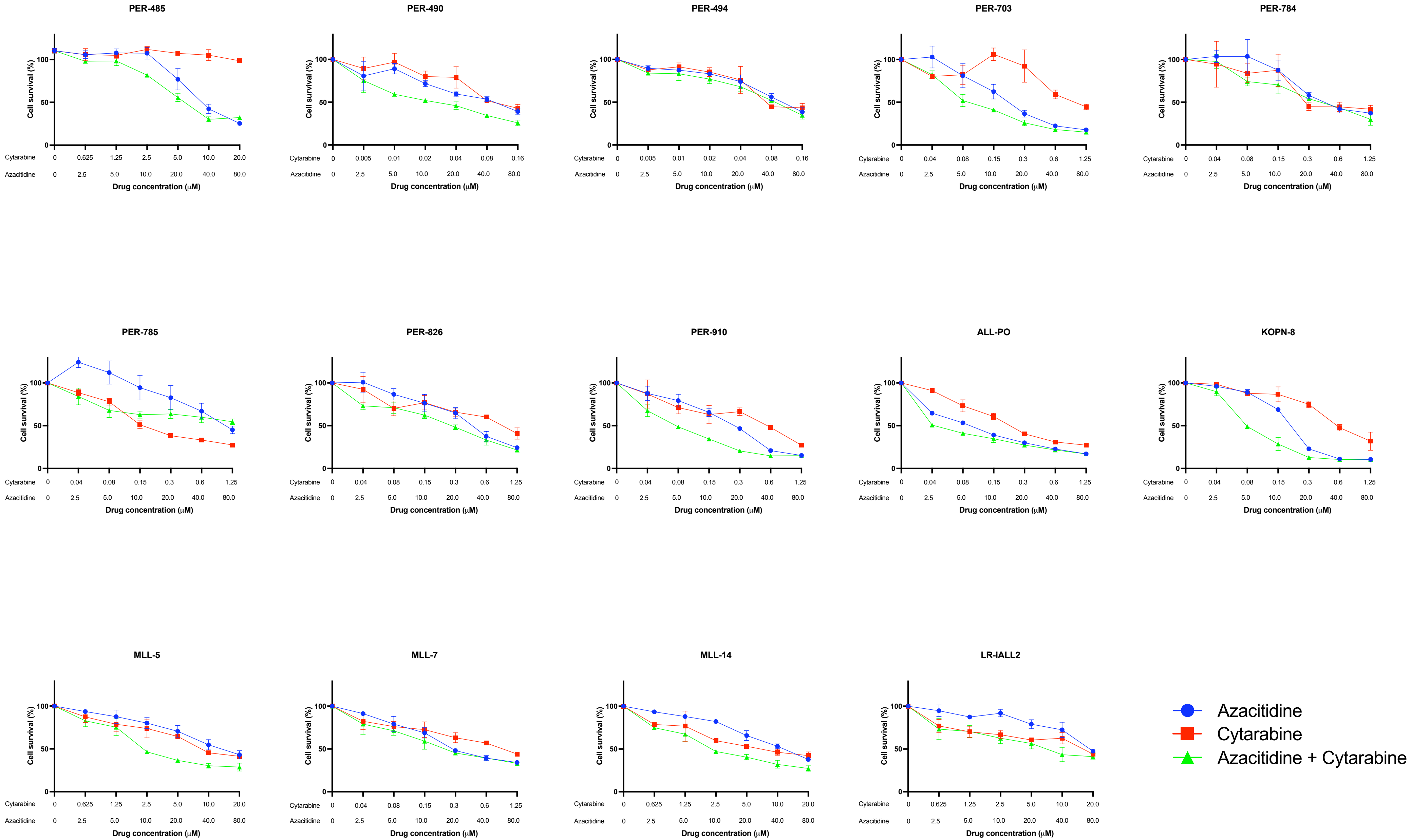
PER-485
Cell survival (%)
Cytarabine 0 0.625 1.25 2.5 5.0 10.0 20.0
Azacitidine 0 2.5 5.0 10.0 20.0 40.0 80.0
Drug concentration (μM)
PER-490
Cell survival (%)
Cytarabine 0 0.005 0.01 0.02 0.04 0.08 0.16
Azacitidine 0 2.5 5.0 10.0 20.0 40.0 80.0
Drug concentration (μM)
PER-494
Cell survival (%)
Cytarabine 0 0.005 0.01 0.02 0.04 0.08 0.16
Azacitidine 0 2.5 5.0 10.0 20.0 40.0 80.0
Drug concentration (μM)
PER-703
Cell survival (%)
Cytarabine 0 0.04 0.08 0.15 0.3 0.6 1.25
Azacitidine 0 2.5 5.0 10.0 20.0 40.0 80.0
Drug concentration (μM)
PER-784
Cell survival (%)
Cytarabine 0 0.04 0.08 0.15 0.3 0.6 1.25
Azacitidine 0 2.5 5.0 10.0 20.0 40.0 80.0
Drug concentration (μM)
PER-785
Cell survival (%)
Cytarabine 0 0.04 0.08 0.15 0.3 0.6 1.25
Azacitidine 0 2.5 5.0 10.0 20.0 40.0 80.0
Drug concentration (μM)
PER-826
Cell survival (%)
Cytarabine 0 0.04 0.08 0.15 0.3 0.6 1.25
Azacitidine 0 2.5 5.0 10.0 20.0 40.0 80.0
Drug concentration (μM)
PER-910
Cell survival (%)
Cytarabine 0 0.04 0.08 0.15 0.3 0.6 1.25
Azacitidine 0 2.5 5.0 10.0 20.0 40.0 80.0
Drug concentration (μM)
ALL-PO
Cell survival (%)
Cytarabine 0 0.04 0.08 0.15 0.3 0.6 1.25
Azacitidine 0 2.5 5.0 10.0 20.0 40.0 80.0
Drug concentration (μM)
KOPN-8
Cell survival (%)
Cytarabine 0 0.04 0.08 0.15 0.3 0.6 1.25
Azacitidine 0 2.5 5.0 10.0 20.0 40.0 80.0
Drug concentration (μM)
MLL-5
Cell survival (%)
Cytarabine 0 0.625 1.25 2.5 5.0 10.0 20.0
Azacitidine 0 2.5 5.0 10.0 20.0 40.0 80.0
Drug concentration (μM)
MLL-7
Cell survival (%)
Cytarabine 0 0.04 0.08 0.15 0.3 0.6 1.25
Azacitidine 0 2.5 5.0 10.0 20.0 40.0 80.0
Drug concentration (μM)
MLL-14
Cell survival (%)
Cytarabine 0 0.625 1.25 2.5 5.0 10.0 20.0
Azacitidine 0 2.5 5.0 10.0 20.0 40.0 80.0
Drug concentration (μM)
LR-iALL2
Cell survival (%)
Cytarabine 0 0.625 1.25 2.5 5.0 10.0 20.0
Azacitidine 0 2.5 5.0 10.0 20.0 40.0 80.0
Drug concentration (μM)
Azacitidine
Cytarabine
Azacitidine + Cytarabine

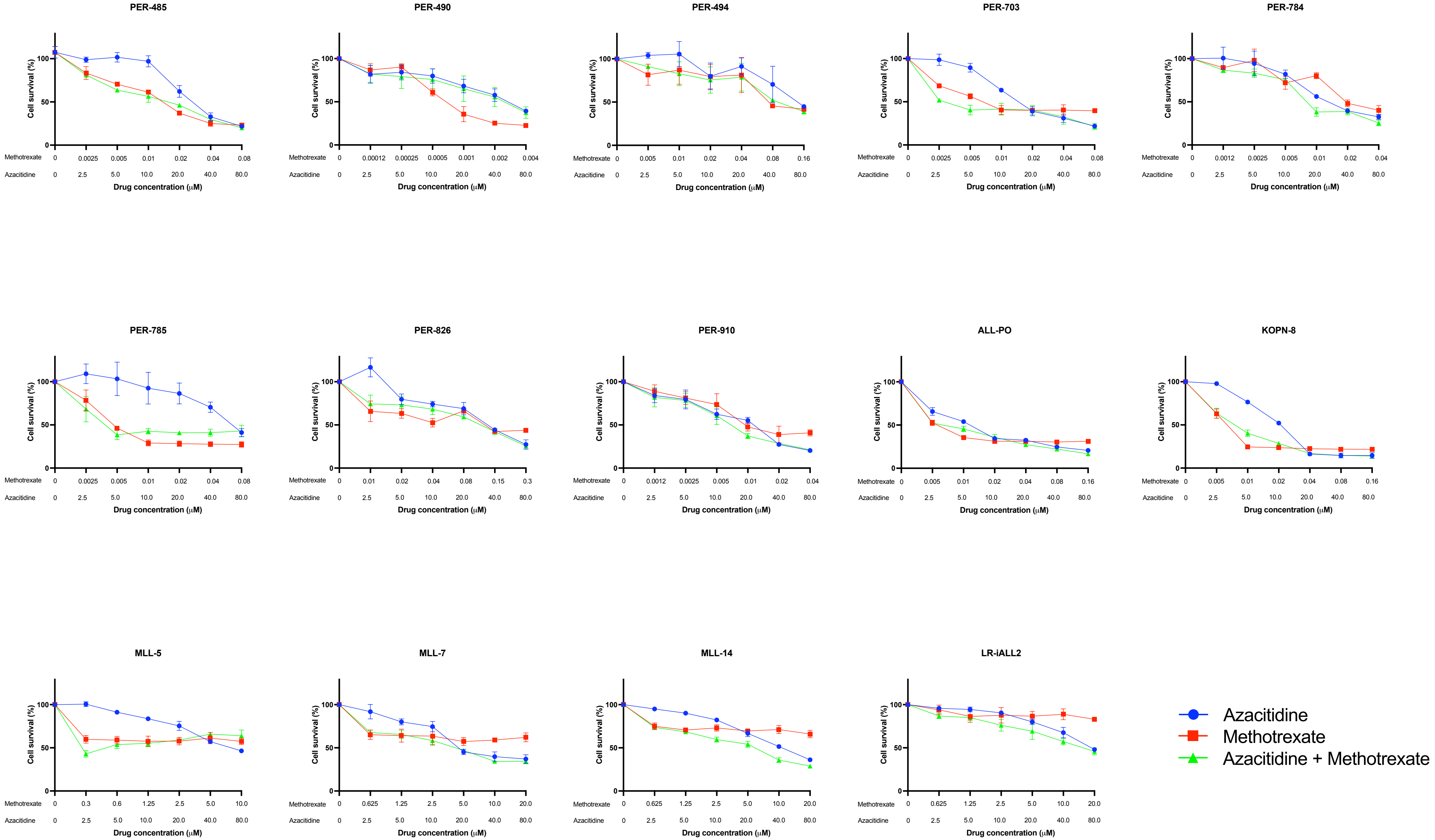
PER-485
Cell survival (%)
Methotrexate 0 0.0025 0.005 0.01 0.02 0.04 0.08
Azacitidine 0 2.5 5.0 10.0 20.0 40.0 80.0
Drug concentration (μM)
PER-490
Cell survival (%)
Methotrexate 0 0.00012 0.00025 0.0005 0.001 0.002 0.004
Azacitidine 0 2.5 5.0 10.0 20.0 40.0 80.0
Drug concentration (μM)
PER-494
Cell survival (%)
Methotrexate 0 0.005 0.01 0.02 0.04 0.08 0.16
Azacitidine 0 2.5 5.0 10.0 20.0 40.0 80.0
Drug concentration (μM)
PER-703
Cell survival (%)
Methotrexate 0 0.0025 0.005 0.01 0.02 0.04 0.08
Azacitidine 0 2.5 5.0 10.0 20.0 40.0 80.0
Drug concentration (μM)
PER-784
Cell survival (%)
Methotrexate 0 0.00012 0.0025 0.005 0.01 0.02 0.04
Azacitidine 0 2.5 5.0 10.0 20.0 40.0 80.0
Drug concentration (μM)
PER-785
Cell survival (%)
Methotrexate 0 0.0025 0.005 0.01 0.02 0.04 0.08
Azacitidine 0 2.5 5.0 10.0 20.0 40.0 80.0
Drug concentration (μM)
PER-826
Cell survival (%)
Methotrexate 0 0.01 0.02 0.04 0.08 0.15 0.3
Azacitidine 0 2.5 5.0 10.0 20.0 40.0 80.0
Drug concentration (μM)
PER-910
Cell survival (%)
Methotrexate 0 0.0012 0.0025 0.005 0.01 0.02 0.04
Azacitidine 0 2.5 5.0 10.0 20.0 40.0 80.0
Drug concentration (μM)
ALL-PO
Cell survival (%)
Methotrexate 0 0.005 0.01 0.02 0.04 0.08 0.16
Azacitidine 0 2.5 5.0 10.0 20.0 40.0 80.0
Drug concentration (μM)
KOPN-8
Cell survival (%)
Methotrexate 0 0.005 0.01 0.02 0.04 0.08 0.16
Azacitidine 0 2.5 5.0 10.0 20.0 40.0 80.0
Drug concentration (μM)
MLL-5
Cell survival (%)
Methotrexate 0 0.3 0.6 1.25 2.5 5.0 10.0
Azacitidine 0 2.5 5.0 10.0 20.0 40.0 80.0
Drug concentration (μM)
MLL-7
Cell survival (%)
Methotrexate 0 0.625 1.25 2.5 5.0 10.0 20.0
Azacitidine 0 2.5 5.0 10.0 20.0 40.0 80.0
Drug concentration (μM)
MLL-14
Cell survival (%)
Methotrexate 0 0.625 1.25 2.5 5.0 10.0 20.0
Azacitidine 0 2.5 5.0 10.0 20.0 40.0 80.0
Drug concentration (μM)
LR-iALL2
Cell survival (%)
Methotrexate 0 0.625 1.25 2.5 5.0 10.0 20.0
Azacitidine 0 2.5 5.0 10.0 20.0 40.0 80.0
Drug concentration (μM)
Azacitidine
Methotrexate
Azacitidine + Methotrexate

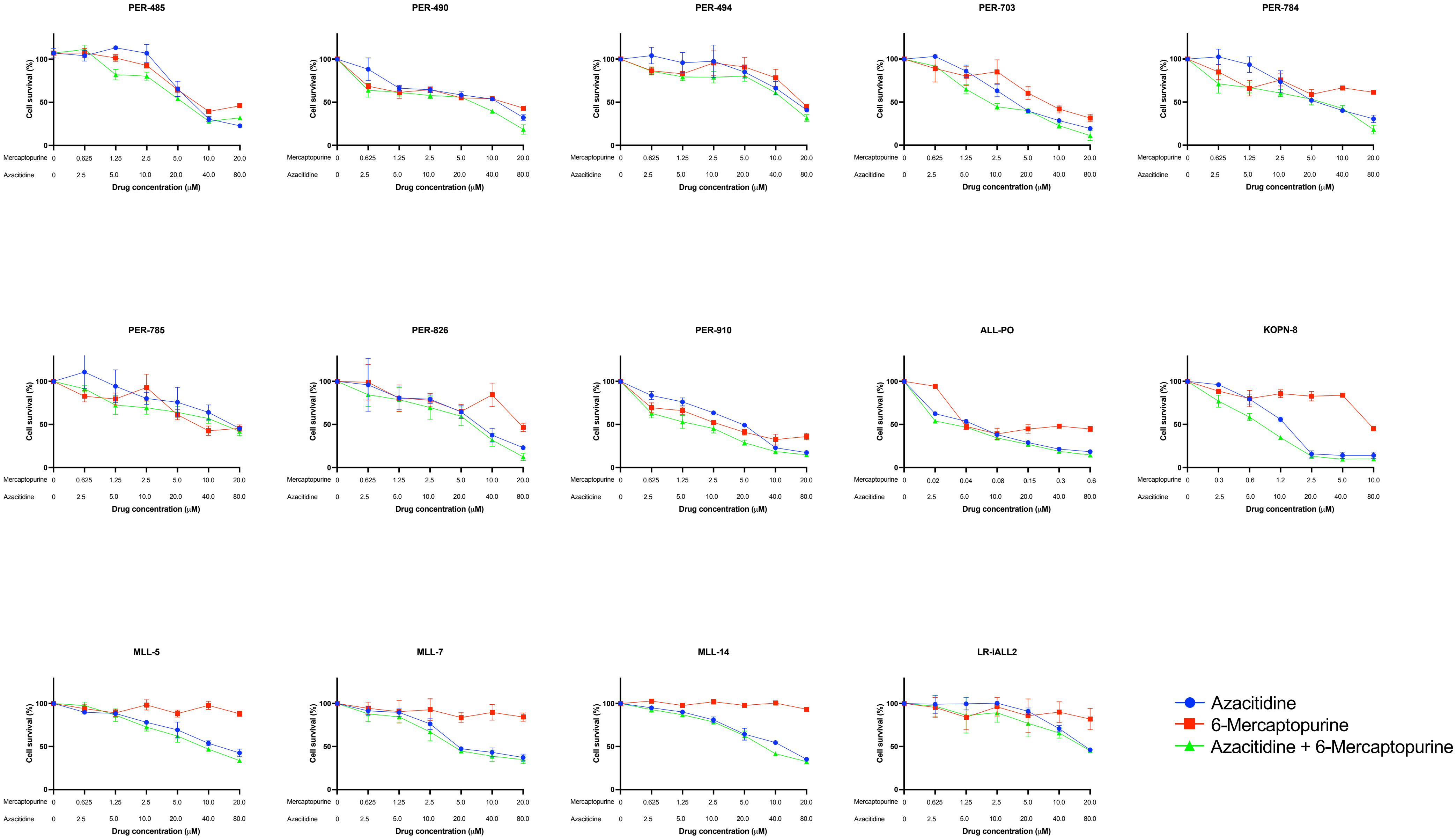
PER-485
PER-490
PER-494
PER-703
PER-784
PER-785
PER-826
PER-910
ALL-PO
KOPN-8
MLL-5
MLL-7
MLL-14
LR-iALL2
Cell survival (%)
0
50
100
Mercaptopurine 0 0.625 1.25 2.5 5.0 10.0 20.0
Azacitidine 0 2.5 5.0 10.0 20.0 40.0 80.0
Mercaptopurine 0 0.02 0.04 0.08 0.15 0.3 0.6
Mercaptopurine 0 0.3 0.6 1.2 2.5 5.0 10.0
Drug concentration (μM)
Azacitidine
6-Mercaptopurine
Azacitidine + 6-Mercaptopurine

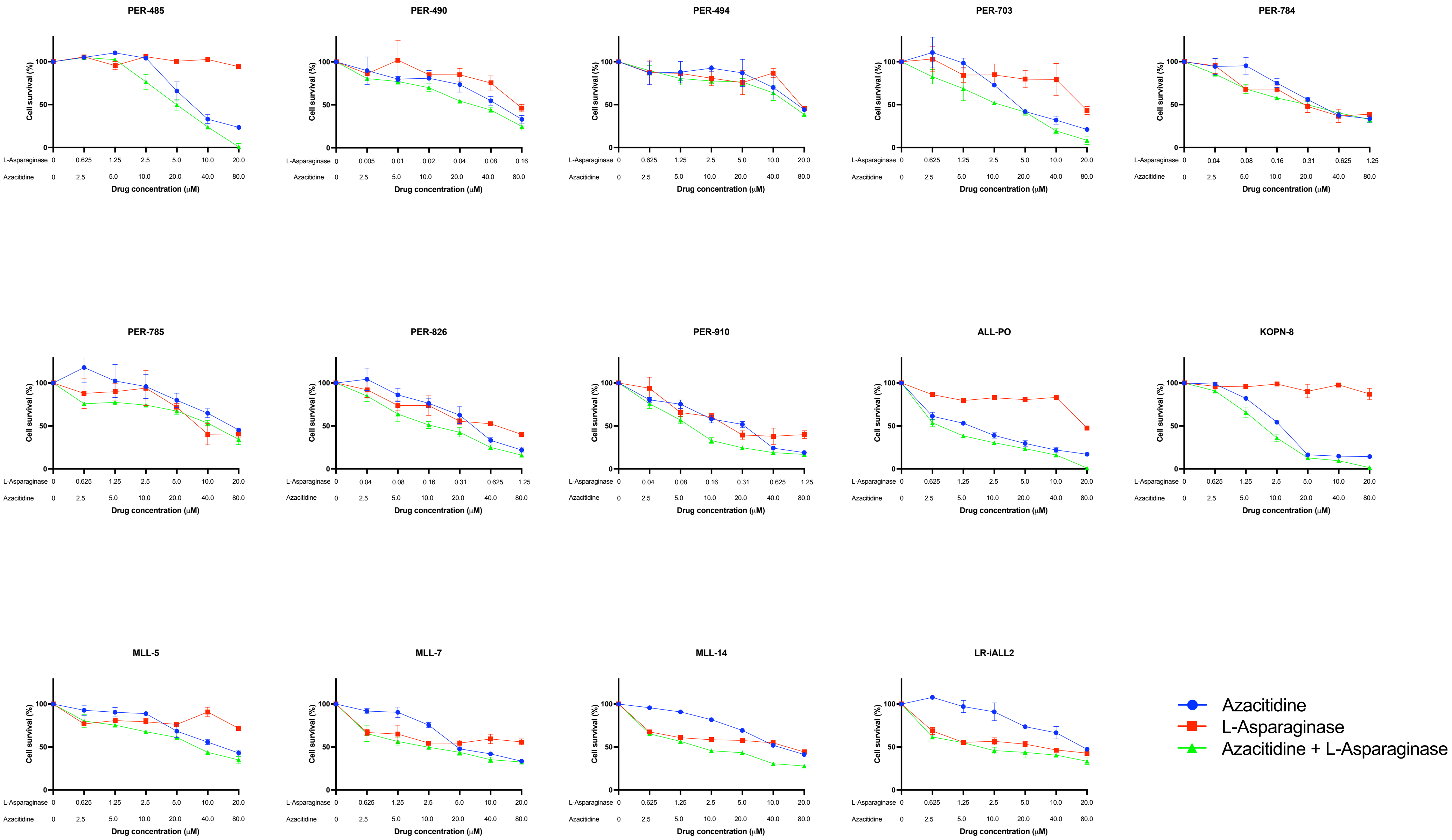

PER-485
PER-490
PER-494
PER-703
PER-784
PER-785
PER-826
PER-910
ALL-PO
KOPN-8
MLL-5
MLL-7
MLL-14
LR-iALL2
Cell survival (%)
L-Asparaginase 0 0.625 1.25 2.5 5.0 10.0 20.0
L-Asparaginase 0 0.005 0.01 0.02 0.04 0.08 0.16
L-Asparaginase 0 0.04 0.08 0.16 0.31 0.625 1.25
Azacitidine 0 2.5 5.0 10.0 20.0 40.0 80.0
Drug concentration (μM)
Azacitidine
L-Asparaginase
Azacitidine + L-Asparaginase

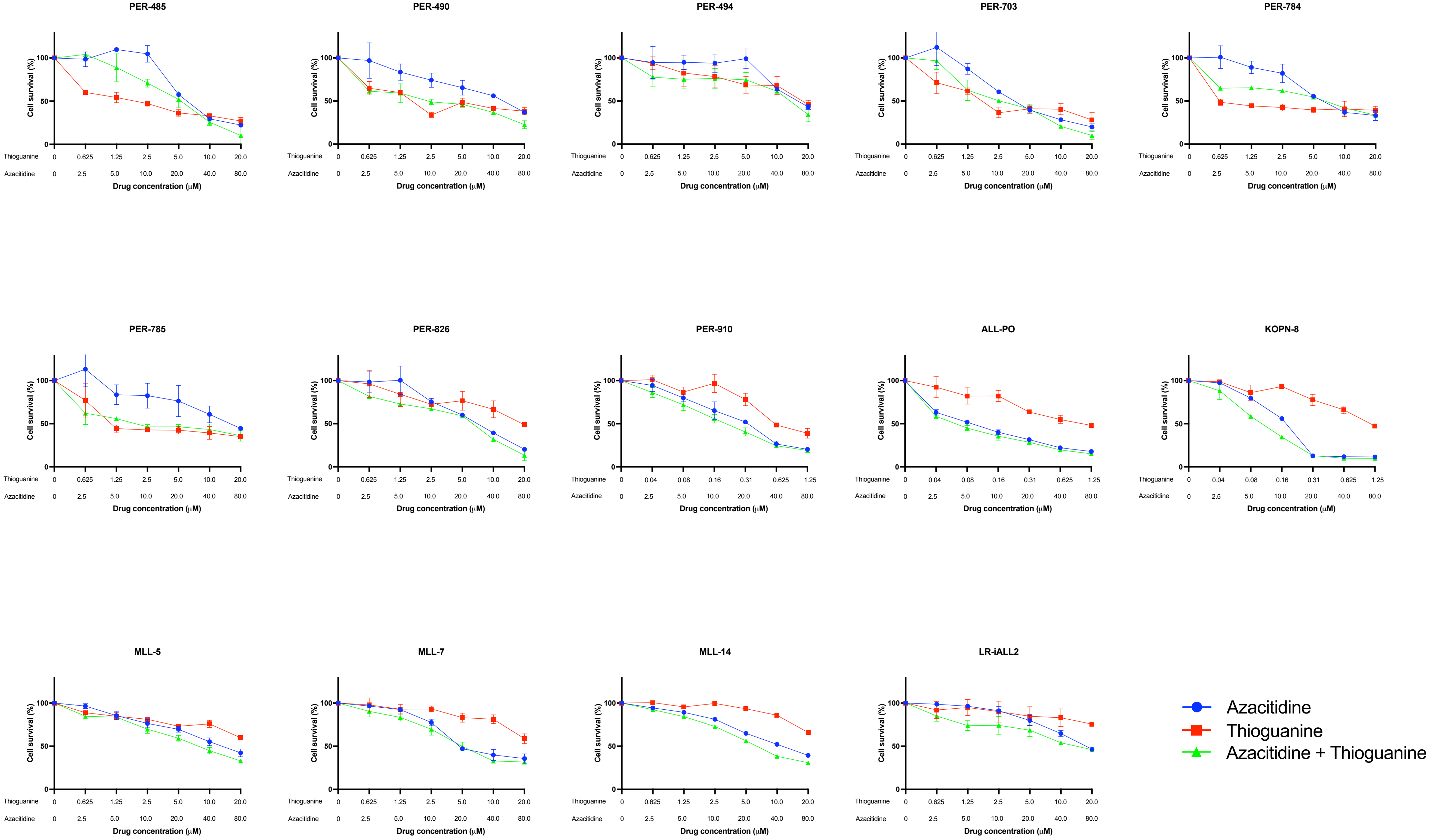

PER-485
PER-490
PER-494
PER-703
PER-784
PER-785
PER-826
PER-910
ALL-PO
KOPN-8
MLL-5
MLL-7
MLL-14
LR-iALL2
Cell survival (%)
Thioguanine
Azacitidine
Drug concentration (μM)
Azacitidine
Thioguanine
Azacitidine + Thioguanine

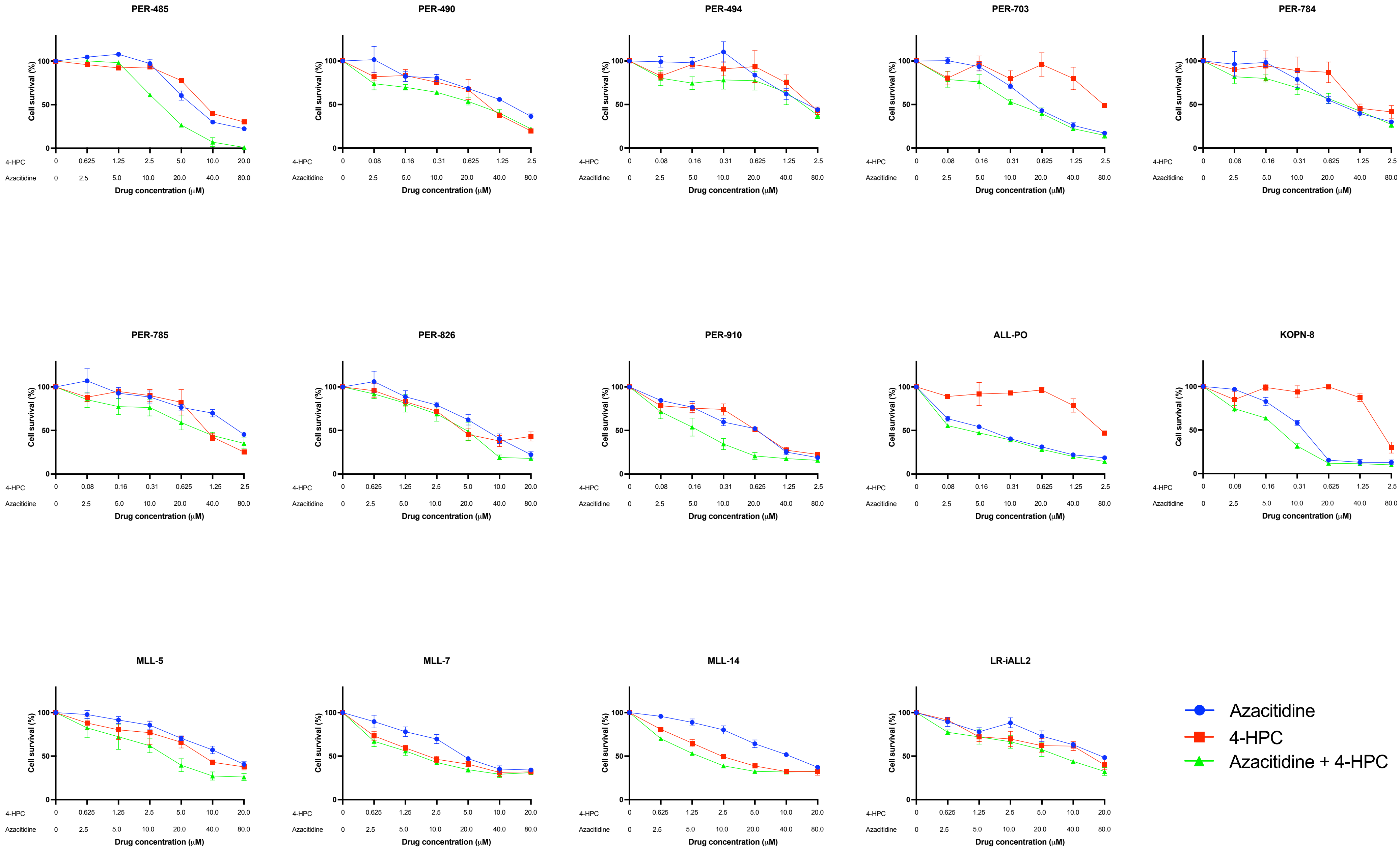

PER-485
PER-490
PER-494
PER-703
PER-784
PER-785
PER-826
PER-910
ALL-PO
KOPN-8
MLL-5
MLL-7
MLL-14
LR-iALL2
Cell survival (%)
0
50
100
4-HPC
0 0.625 1.25 2.5 5.0 10.0 20.0
0 0.08 0.16 0.31 0.625 1.25 2.5
Azacitidine
0 2.5 5.0 10.0 20.0 40.0 80.0
Drug concentration (µM)
Azacitidine
4-HPC
Azacitidine + 4-HPC

B

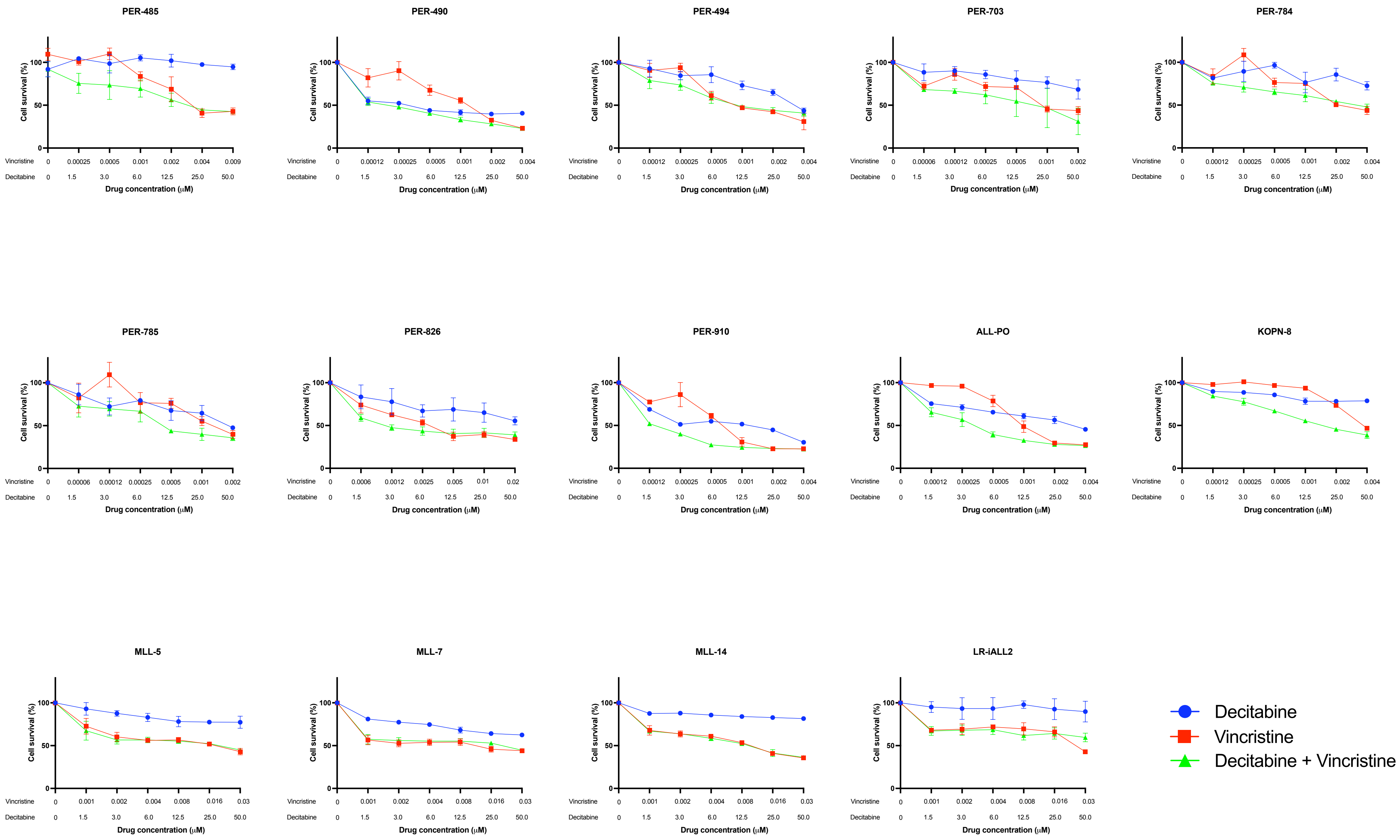
PER-485
PER-490
PER-494
PER-703
PER-784
PER-785
PER-826
PER-910
ALL-PO
KOPN-8
MLL-5
MLL-7
MLL-14
LR-iALL2
Cell survival (%)
Vincristine
Decitabine
Drug concentration (μM)
Decitabine
Vincristine
Decitabine + Vincristine

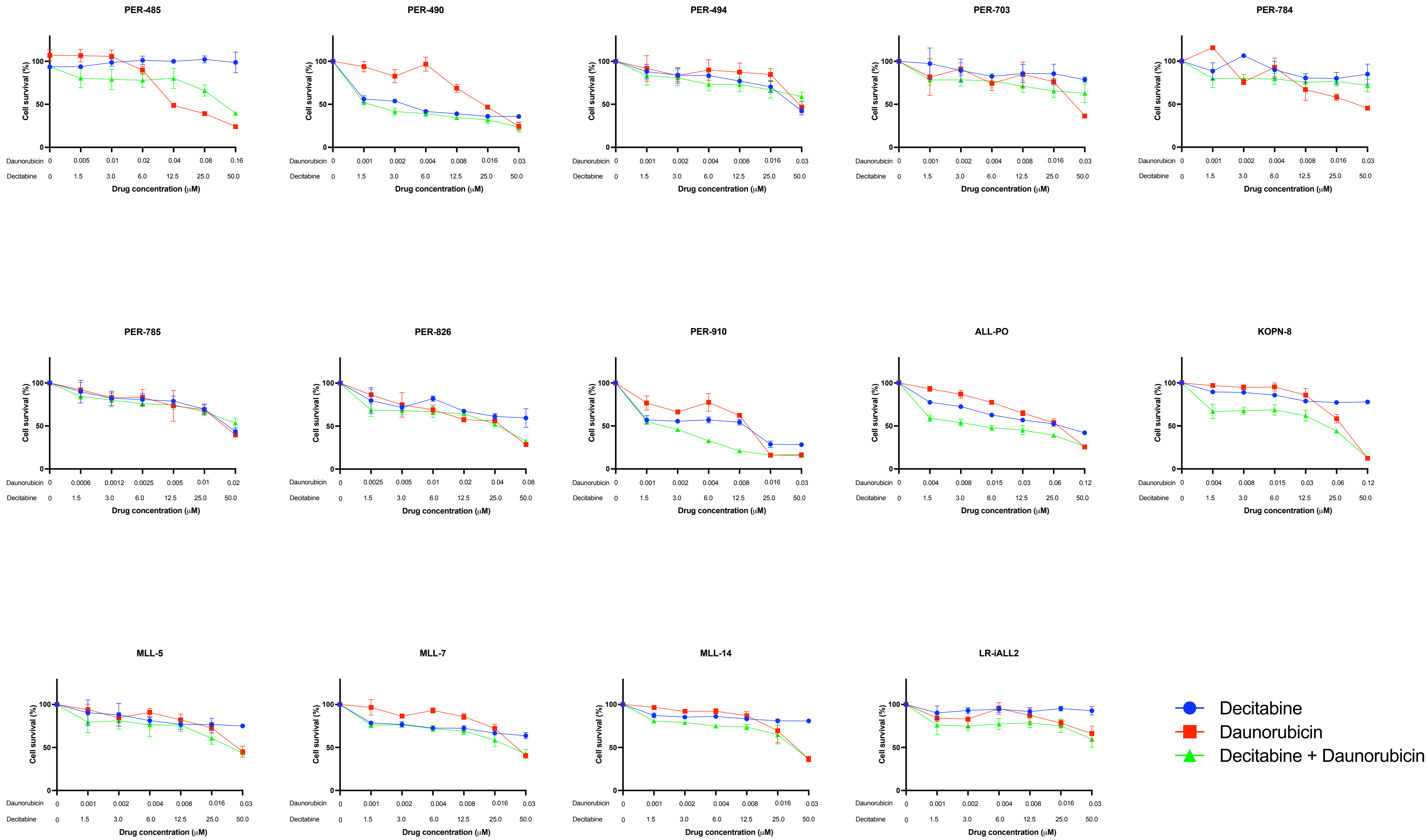
PER-485
Cell survival (%)
Daunorubicin 0 0.005 0.01 0.02 0.04 0.08 0.16
Decitabine 0 1.5 3.0 6.0 12.5 25.0 50.0
Drug concentration (μM)
PER-490
Daunorubicin 0 0.001 0.002 0.004 0.008 0.016 0.03
Decitabine 0 1.5 3.0 6.0 12.5 25.0 50.0
PER-494
Daunorubicin 0 0.001 0.002 0.004 0.008 0.016 0.03
Decitabine 0 1.5 3.0 6.0 12.5 25.0 50.0
PER-703
Daunorubicin 0 0.001 0.002 0.004 0.008 0.016 0.03
Decitabine 0 1.5 3.0 6.0 12.5 25.0 50.0
PER-784
Daunorubicin 0 0.001 0.002 0.004 0.008 0.016 0.03
Decitabine 0 1.5 3.0 6.0 12.5 25.0 50.0
PER-785
Daunorubicin 0 0.0006 0.0012 0.0025 0.005 0.01 0.02
Decitabine 0 1.5 3.0 6.0 12.5 25.0 50.0
PER-826
Daunorubicin 0 0.0025 0.005 0.01 0.02 0.04 0.08
Decitabine 0 1.5 3.0 6.0 12.5 25.0 50.0
PER-910
Daunorubicin 0 0.001 0.002 0.004 0.008 0.016 0.03
Decitabine 0 1.5 3.0 6.0 12.5 25.0 50.0
ALL-PO
Daunorubicin 0 0.004 0.008 0.015 0.03 0.06 0.12
Decitabine 0 1.5 3.0 6.0 12.5 25.0 50.0
KOPN-8
Daunorubicin 0 0.004 0.008 0.015 0.03 0.06 0.12
Decitabine 0 1.5 3.0 6.0 12.5 25.0 50.0
MLL-5
Daunorubicin 0 0.001 0.002 0.004 0.008 0.016 0.03
Decitabine 0 1.5 3.0 6.0 12.5 25.0 50.0
MLL-7
Daunorubicin 0 0.001 0.002 0.004 0.008 0.016 0.03
Decitabine 0 1.5 3.0 6.0 12.5 25.0 50.0
MLL-14
Daunorubicin 0 0.001 0.002 0.004 0.008 0.016 0.03
Decitabine 0 1.5 3.0 6.0 12.5 25.0 50.0
LR-iALL2
Daunorubicin 0 0.001 0.002 0.004 0.008 0.016 0.03
Decitabine 0 1.5 3.0 6.0 12.5 25.0 50.0
Decitabine
Daunorubicin
Decitabine + Daunorubicin

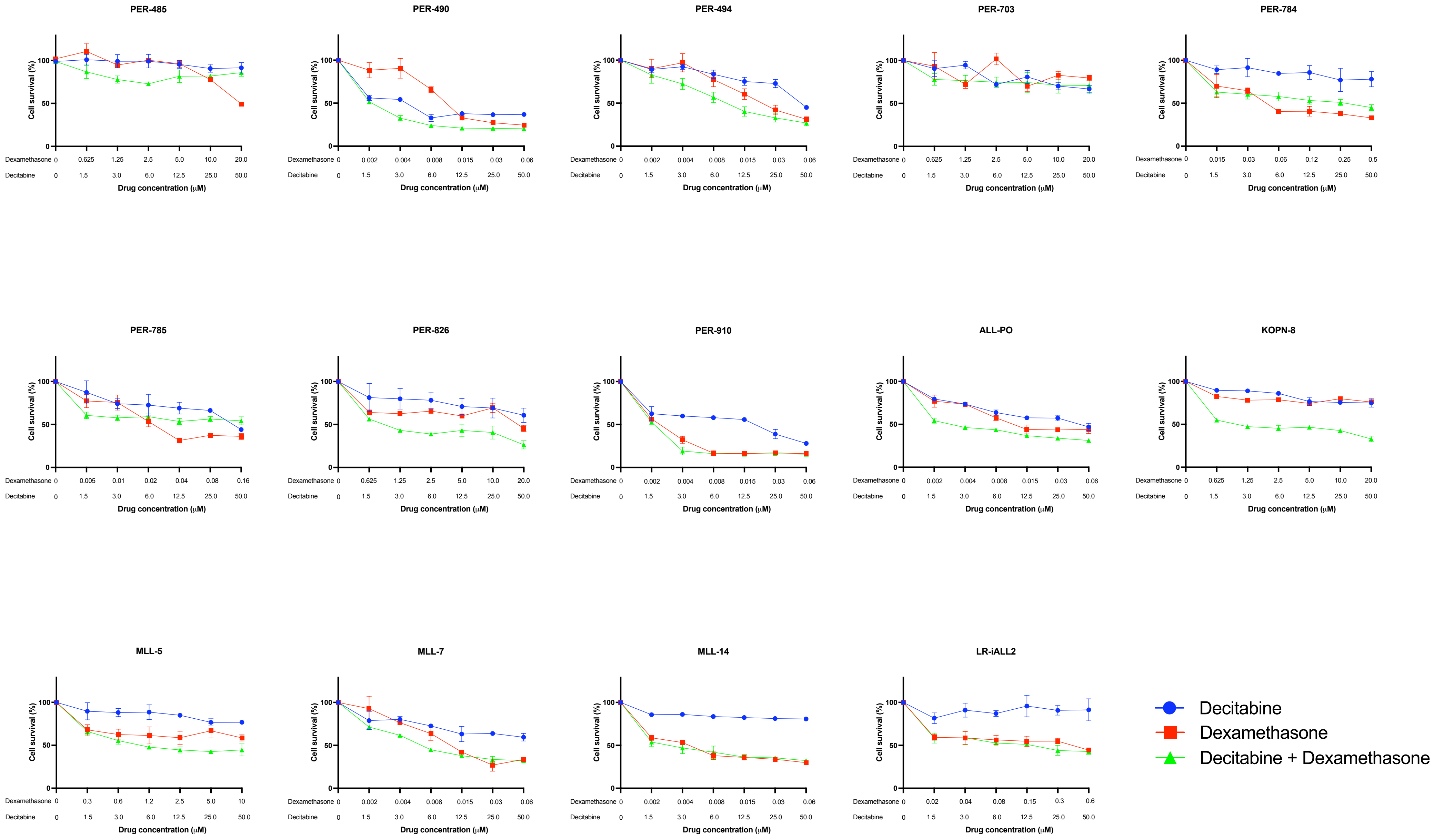
PER-485
Cell survival (%)
Dexamethasone 0 0.625 1.25 2.5 5.0 10.0 20.0
Decitabine 0 1.5 3.0 6.0 12.5 25.0 50.0
Drug concentration (μM)
PER-490
Dexamethasone 0 0.002 0.004 0.008 0.015 0.03 0.06
Decitabine 0 1.5 3.0 6.0 12.5 25.0 50.0
PER-494
Dexamethasone 0 0.002 0.004 0.008 0.015 0.03 0.06
Decitabine 0 1.5 3.0 6.0 12.5 25.0 50.0
PER-703
Dexamethasone 0 0.625 1.25 2.5 5.0 10.0 20.0
Decitabine 0 1.5 3.0 6.0 12.5 25.0 50.0
PER-784
Dexamethasone 0 0.015 0.03 0.06 0.12 0.25 0.5
Decitabine 0 1.5 3.0 6.0 12.5 25.0 50.0
PER-785
Dexamethasone 0 0.005 0.01 0.02 0.04 0.08 0.16
Decitabine 0 1.5 3.0 6.0 12.5 25.0 50.0
PER-826
Dexamethasone 0 0.625 1.25 2.5 5.0 10.0 20.0
Decitabine 0 1.5 3.0 6.0 12.5 25.0 50.0
PER-910
Dexamethasone 0 0.002 0.004 0.008 0.015 0.03 0.06
Decitabine 0 1.5 3.0 6.0 12.5 25.0 50.0
ALL-PO
Dexamethasone 0 0.002 0.004 0.008 0.015 0.03 0.06
Decitabine 0 1.5 3.0 6.0 12.5 25.0 50.0
KOPN-8
Dexamethasone 0 0.625 1.25 2.5 5.0 10.0 20.0
Decitabine 0 1.5 3.0 6.0 12.5 25.0 50.0
MLL-5
Dexamethasone 0 0.3 0.6 1.2 2.5 5.0 10
Decitabine 0 1.5 3.0 6.0 12.5 25.0 50.0
MLL-7
Dexamethasone 0 0.002 0.004 0.008 0.015 0.03 0.06
Decitabine 0 1.5 3.0 6.0 12.5 25.0 50.0
MLL-14
Dexamethasone 0 0.002 0.004 0.008 0.015 0.03 0.06
Decitabine 0 1.5 3.0 6.0 12.5 25.0 50.0
LR-iALL2
Dexamethasone 0 0.02 0.04 0.08 0.15 0.3 0.6
Decitabine 0 1.5 3.0 6.0 12.5 25.0 50.0
Decitabine
Dexamethasone
Decitabine + Dexamethasone

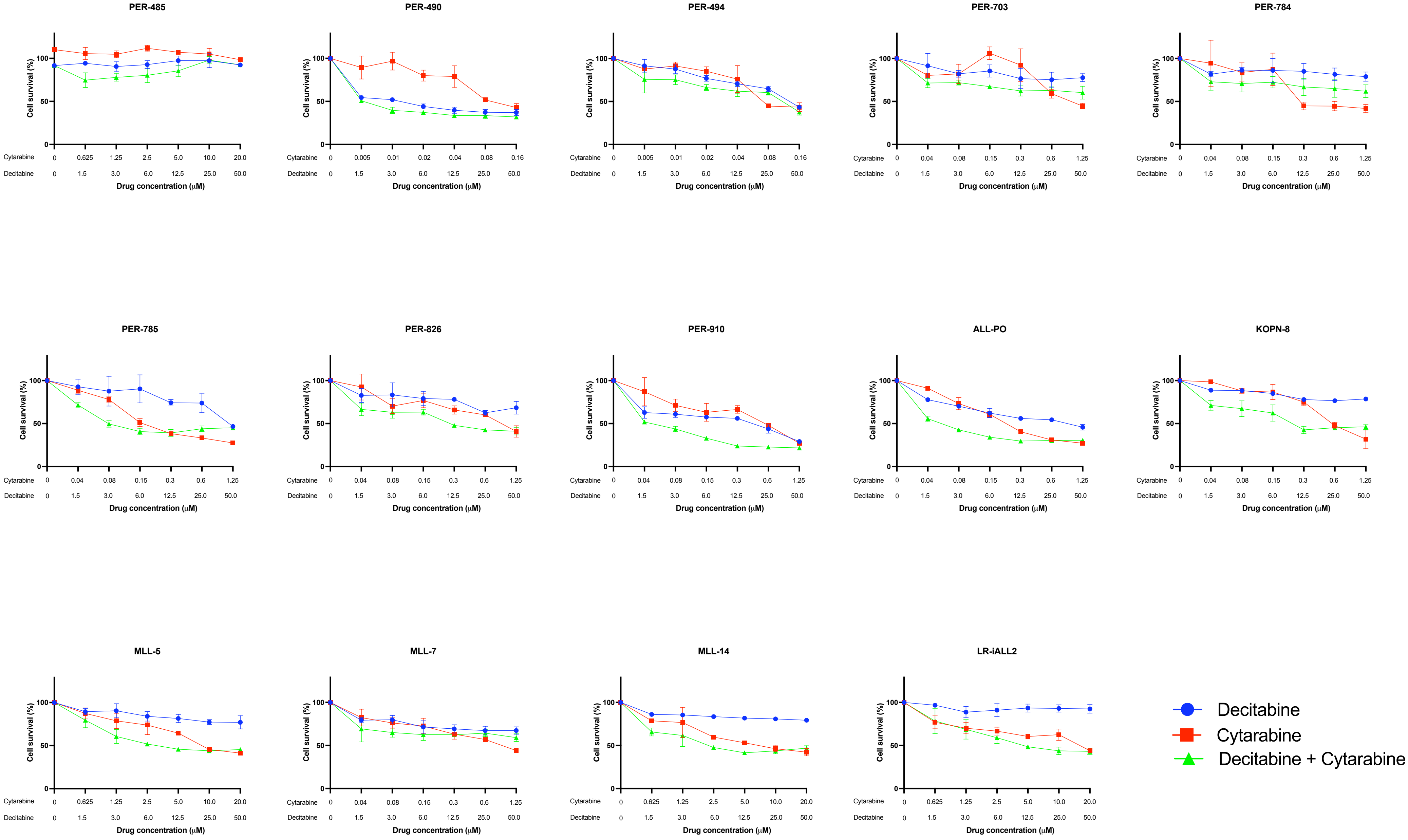
PER-485
PER-490
PER-494
PER-703
PER-784
PER-785
PER-826
PER-910
ALL-PO
KOPN-8
MLL-5
MLL-7
MLL-14
LR-iALL2
Cell survival (%)
Cytarabine
Decitabine
Drug concentration (µM)
Decitabine
Cytarabine
Decitabine + Cytarabine

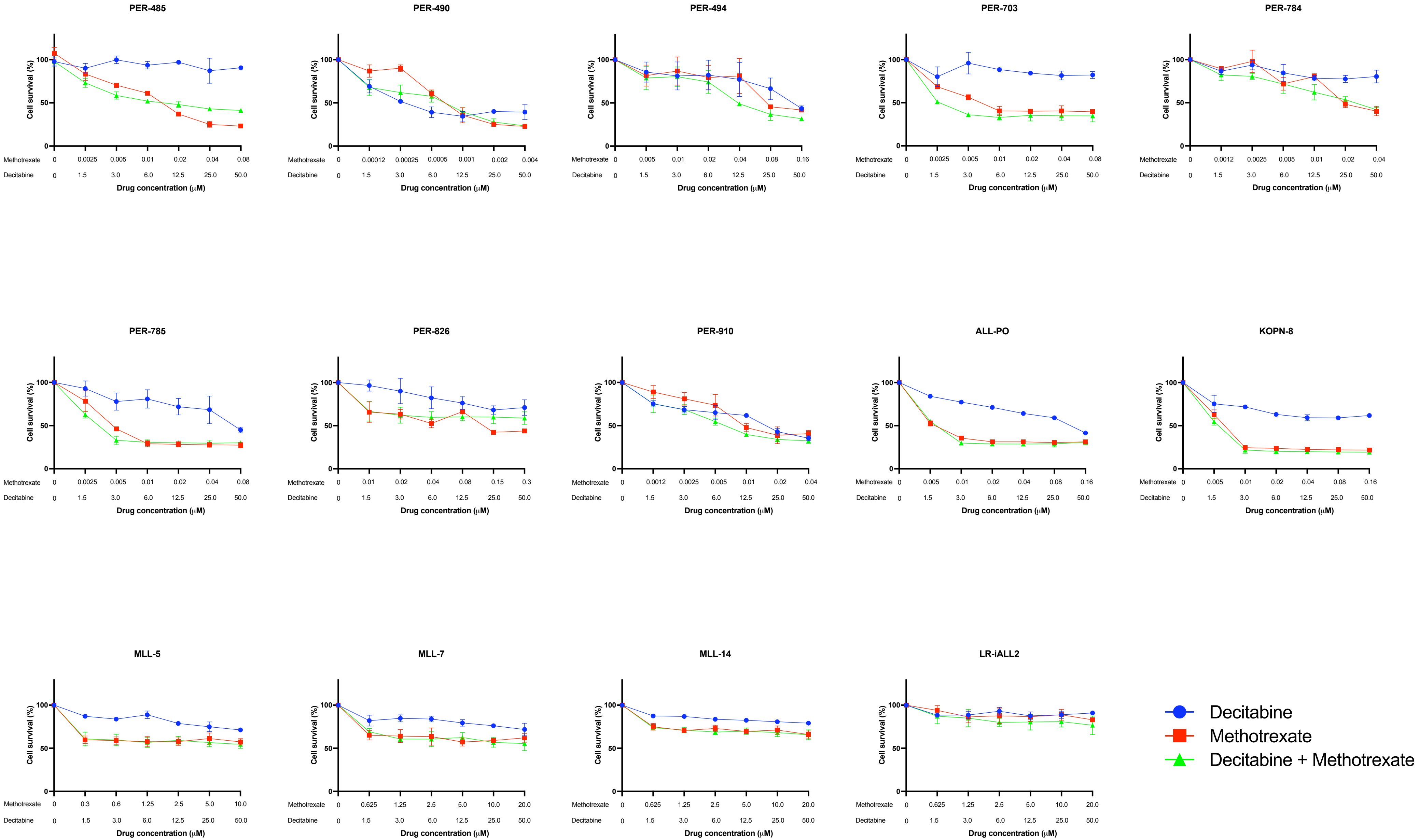
PER-485
Cell survival (%)
0
50
100
Methotrexate 0 0.0025 0.005 0.01 0.02 0.04 0.08
Decitabine 0 1.5 3.0 6.0 12.5 25.0 50.0
Drug concentration (μM)
PER-490
Cell survival (%)
0
50
100
Methotrexate 0 0.00012 0.00025 0.0005 0.001 0.002 0.004
Decitabine 0 1.5 3.0 6.0 12.5 25.0 50.0
Drug concentration (μM)
PER-494
Cell survival (%)
0
50
100
Methotrexate 0 0.005 0.01 0.02 0.04 0.08 0.16
Decitabine 0 1.5 3.0 6.0 12.5 25.0 50.0
Drug concentration (μM)
PER-703
Cell survival (%)
0
50
100
Methotrexate 0 0.0025 0.005 0.01 0.02 0.04 0.08
Decitabine 0 1.5 3.0 6.0 12.5 25.0 50.0
Drug concentration (μM)
PER-784
Cell survival (%)
0
50
100
Methotrexate 0 0.0012 0.0025 0.005 0.01 0.02 0.04
Decitabine 0 1.5 3.0 6.0 12.5 25.0 50.0
Drug concentration (μM)
PER-785
Cell survival (%)
0
50
100
Methotrexate 0 0.0025 0.005 0.01 0.02 0.04 0.08
Decitabine 0 1.5 3.0 6.0 12.5 25.0 50.0
Drug concentration (μM)
PER-826
Cell survival (%)
0
50
100
Methotrexate 0 0.01 0.02 0.04 0.08 0.15 0.3
Decitabine 0 1.5 3.0 6.0 12.5 25.0 50.0
Drug concentration (μM)
PER-910
Cell survival (%)
0
50
100
Methotrexate 0 0.0012 0.0025 0.005 0.01 0.02 0.04
Decitabine 0 1.5 3.0 6.0 12.5 25.0 50.0
Drug concentration (μM)
ALL-PO
Cell survival (%)
0
50
100
Methotrexate 0 0.005 0.01 0.02 0.04 0.08 0.16
Decitabine 0 1.5 3.0 6.0 12.5 25.0 50.0
Drug concentration (μM)
KOPN-8
Cell survival (%)
0
50
100
Methotrexate 0 0.005 0.01 0.02 0.04 0.08 0.16
Decitabine 0 1.5 3.0 6.0 12.5 25.0 50.0
Drug concentration (μM)
MLL-5
Cell survival (%)
0
50
100
Methotrexate 0 0.3 0.6 1.25 2.5 5.0 10.0
Decitabine 0 1.5 3.0 6.0 12.5 25.0 50.0
Drug concentration (μM)
MLL-7
Cell survival (%)
0
50
100
Methotrexate 0 0.625 1.25 2.5 5.0 10.0 20.0
Decitabine 0 1.5 3.0 6.0 12.5 25.0 50.0
Drug concentration (μM)
MLL-14
Cell survival (%)
0
50
100
Methotrexate 0 0.625 1.25 2.5 5.0 10.0 20.0
Decitabine 0 1.5 3.0 6.0 12.5 25.0 50.0
Drug concentration (μM)
LR-iALL2
Cell survival (%)
0
50
100
Methotrexate 0 0.625 1.25 2.5 5.0 10.0 20.0
Decitabine 0 1.5 3.0 6.0 12.5 25.0 50.0
Drug concentration (μM)
Decitabine
Methotrexate
Decitabine + Methotrexate

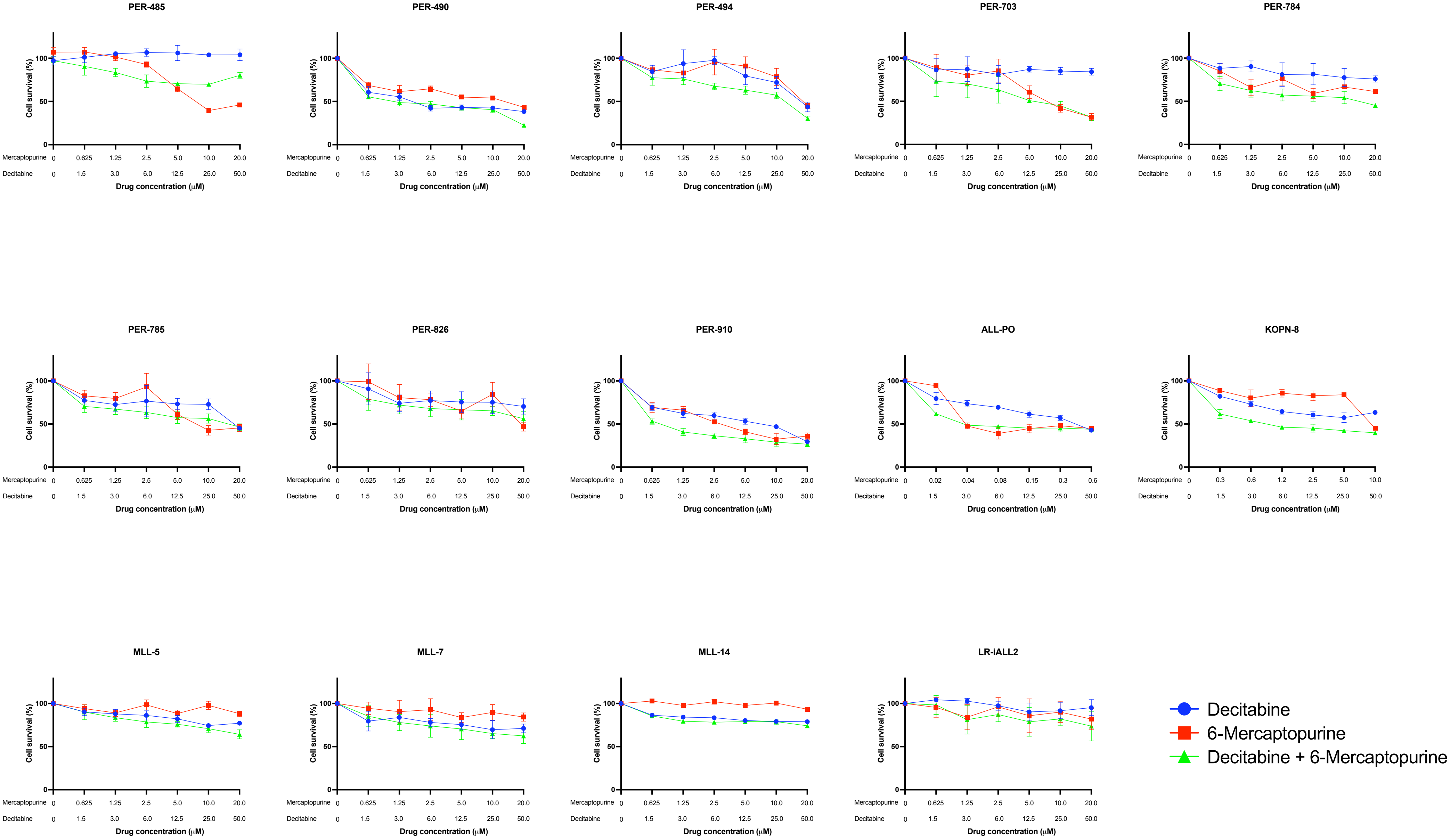
PER-485
PER-490
PER-494
PER-703
PER-784
PER-785
PER-826
PER-910
ALL-PO
KOPN-8
MLL-5
MLL-7
MLL-14
LR-iALL2
Cell survival (%)
0
50
100
Mercaptopurine 0 0.625 1.25 2.5 5.0 10.0 20.0
Decitabine 0 1.5 3.0 6.0 12.5 25.0 50.0
Mercaptopurine 0 0.02 0.04 0.08 0.15 0.3 0.6
Mercaptopurine 0 0.3 0.6 1.2 2.5 5.0 10.0
Drug concentration (μM)
Decitabine
6-Mercaptopurine
Decitabine + 6-Mercaptopurine

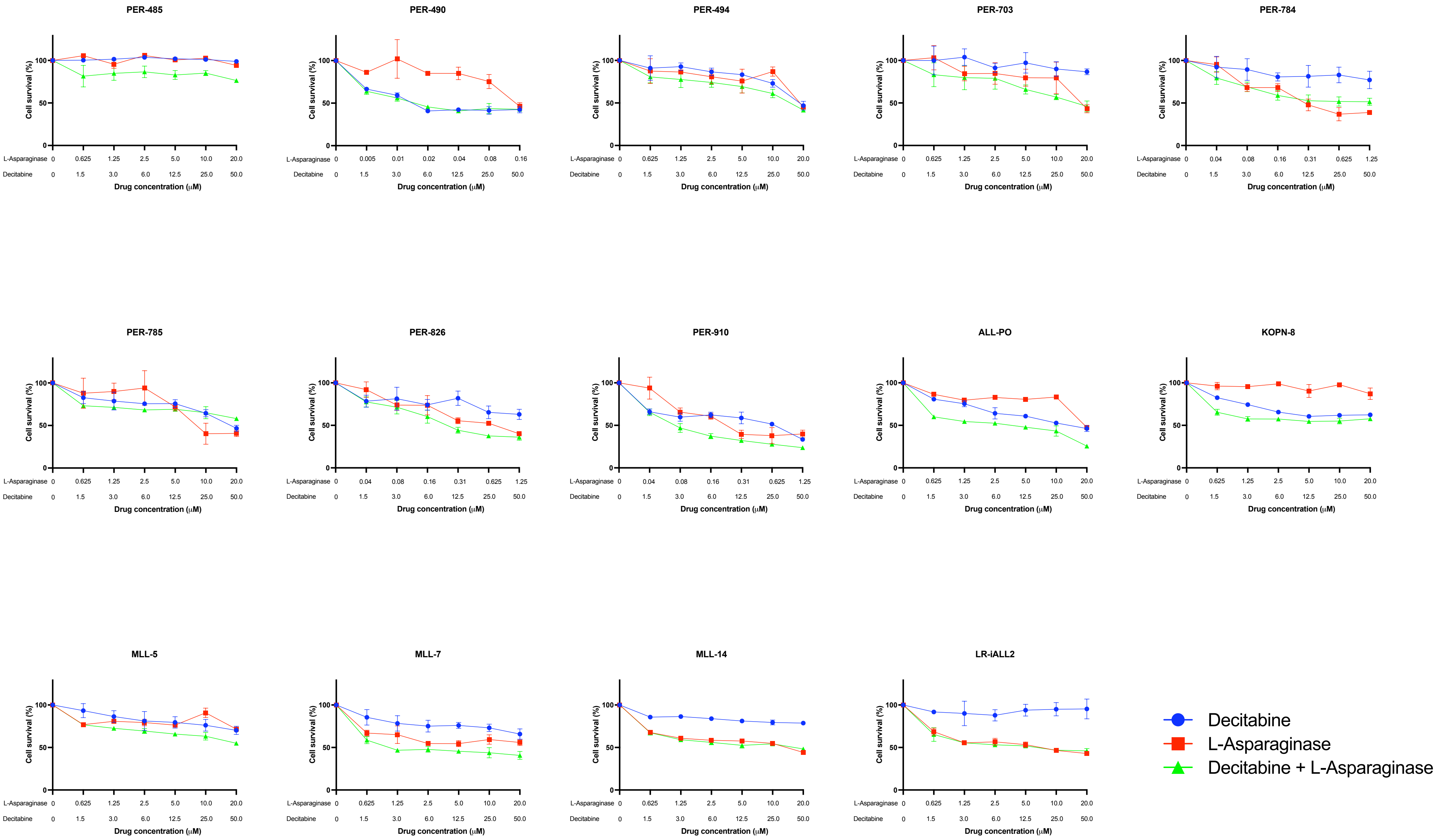
PER-485
Cell survival (%)
100
50
0
L-Asparaginase 0 0.625 1.25 2.5 5.0 10.0 20.0
Decitabine 0 1.5 3.0 6.0 12.5 25.0 50.0
Drug concentration (μM)
PER-490
L-Asparaginase 0 0.005 0.01 0.02 0.04 0.08 0.16
Decitabine 0 1.5 3.0 6.0 12.5 25.0 50.0
PER-494
L-Asparaginase 0 0.625 1.25 2.5 5.0 10.0 20.0
Decitabine 0 1.5 3.0 6.0 12.5 25.0 50.0
PER-703
L-Asparaginase 0 0.625 1.25 2.5 5.0 10.0 20.0
Decitabine 0 1.5 3.0 6.0 12.5 25.0 50.0
PER-784
L-Asparaginase 0 0.04 0.08 0.16 0.31 0.625 1.25
Decitabine 0 1.5 3.0 6.0 12.5 25.0 50.0
PER-785
L-Asparaginase 0 0.625 1.25 2.5 5.0 10.0 20.0
Decitabine 0 1.5 3.0 6.0 12.5 25.0 50.0
PER-826
L-Asparaginase 0 0.04 0.08 0.16 0.31 0.625 1.25
Decitabine 0 1.5 3.0 6.0 12.5 25.0 50.0
PER-910
L-Asparaginase 0 0.04 0.08 0.16 0.31 0.625 1.25
Decitabine 0 1.5 3.0 6.0 12.5 25.0 50.0
ALL-PO
L-Asparaginase 0 0.625 1.25 2.5 5.0 10.0 20.0
Decitabine 0 1.5 3.0 6.0 12.5 25.0 50.0
KOPN-8
L-Asparaginase 0 0.625 1.25 2.5 5.0 10.0 20.0
Decitabine 0 1.5 3.0 6.0 12.5 25.0 50.0
MLL-5
L-Asparaginase 0 0.625 1.25 2.5 5.0 10.0 20.0
Decitabine 0 1.5 3.0 6.0 12.5 25.0 50.0
MLL-7
L-Asparaginase 0 0.625 1.25 2.5 5.0 10.0 20.0
Decitabine 0 1.5 3.0 6.0 12.5 25.0 50.0
MLL-14
L-Asparaginase 0 0.625 1.25 2.5 5.0 10.0 20.0
Decitabine 0 1.5 3.0 6.0 12.5 25.0 50.0
LR-iALL2
L-Asparaginase 0 0.625 1.25 2.5 5.0 10.0 20.0
Decitabine 0 1.5 3.0 6.0 12.5 25.0 50.0
Decitabine
L-Asparaginase
Decitabine + L-Asparaginase

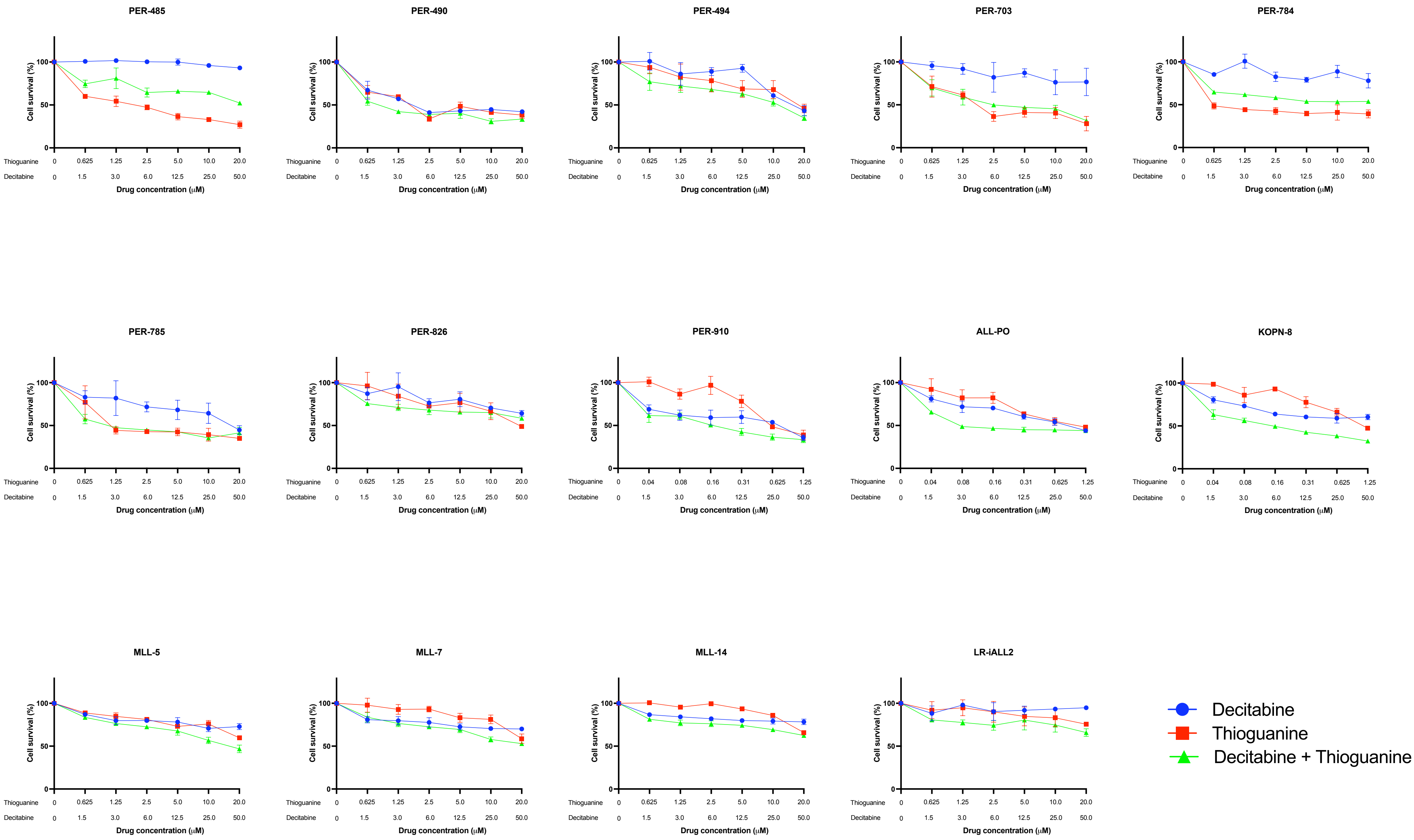
PER-485
PER-490
PER-494
PER-703
PER-784
Cell survival (%)
0
50
100
Thioguanine 0 0.625 1.25 2.5 5.0 10.0 20.0
Decitabine 0 1.5 3.0 6.0 12.5 25.0 50.0
Drug concentration (μM)
PER-785
PER-826
Thioguanine 0 0.625 1.25 2.5 5.0 10.0 20.0
Decitabine 0 1.5 3.0 6.0 12.5 25.0 50.0
PER-910
ALL-PO
KOPN-8
Thioguanine 0 0.04 0.08 0.16 0.31 0.625 1.25
Decitabine 0 1.5 3.0 6.0 12.5 25.0 50.0
MLL-5
MLL-7
MLL-14
LR-iALL2
Thioguanine 0 0.625 1.25 2.5 5.0 10.0 20.0
Decitabine 0 1.5 3.0 6.0 12.5 25.0 50.0
Drug concentration (μM)
Decitabine
Thioguanine
Decitabine + Thioguanine

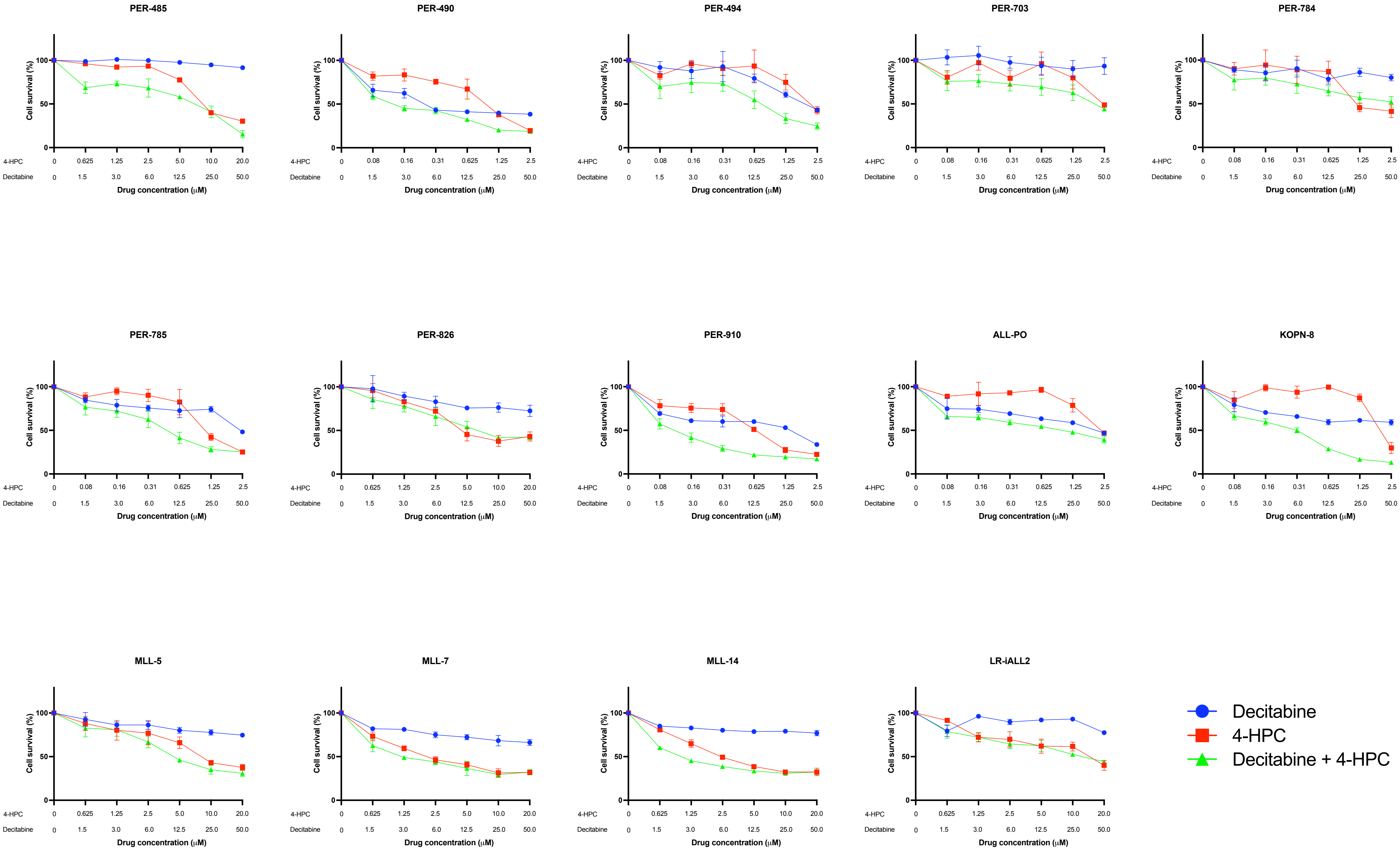
PER-485
Cell survival (%)
0 50 100
4-HPC 0 0.625 1.25 2.5 5.0 10.0 20.0
Decitabine 0 1.5 3.0 6.0 12.5 25.0 50.0
Drug concentration (µM)
PER-490
Cell survival (%)
0 50 100
4-HPC 0 0.08 0.16 0.31 0.625 1.25 2.5
Decitabine 0 1.5 3.0 6.0 12.5 25.0 50.0
Drug concentration (µM)
PER-494
Cell survival (%)
0 50 100
4-HPC 0 0.08 0.16 0.31 0.625 1.25 2.5
Decitabine 0 1.5 3.0 6.0 12.5 25.0 50.0
Drug concentration (µM)
PER-703
Cell survival (%)
0 50 100
4-HPC 0 0.08 0.16 0.31 0.625 1.25 2.5
Decitabine 0 1.5 3.0 6.0 12.5 25.0 50.0
Drug concentration (µM)
PER-784
Cell survival (%)
0 50 100
4-HPC 0 0.08 0.16 0.31 0.625 1.25 2.5
Decitabine 0 1.5 3.0 6.0 12.5 25.0 50.0
Drug concentration (µM)
PER-785
Cell survival (%)
0 50 100
4-HPC 0 0.08 0.16 0.31 0.625 1.25 2.5
Decitabine 0 1.5 3.0 6.0 12.5 25.0 50.0
Drug concentration (µM)
PER-826
Cell survival (%)
0 50 100
4-HPC 0 0.625 1.25 2.5 5.0 10.0 20.0
Decitabine 0 1.5 3.0 6.0 12.5 25.0 50.0
Drug concentration (µM)
PER-910
Cell survival (%)
0 50 100
4-HPC 0 0.08 0.16 0.31 0.625 1.25 2.5
Decitabine 0 1.5 3.0 6.0 12.5 25.0 50.0
Drug concentration (µM)
ALL-PO
Cell survival (%)
0 50 100
4-HPC 0 0.08 0.16 0.31 0.625 1.25 2.5
Decitabine 0 1.5 3.0 6.0 12.5 25.0 50.0
Drug concentration (µM)
KOPN-8
Cell survival (%)
0 50 100
4-HPC 0 0.08 0.16 0.31 0.625 1.25 2.5
Decitabine 0 1.5 3.0 6.0 12.5 25.0 50.0
Drug concentration (µM)
MLL-5
Cell survival (%)
0 50 100
4-HPC 0 0.625 1.25 2.5 5.0 10.0 20.0
Decitabine 0 1.5 3.0 6.0 12.5 25.0 50.0
Drug concentration (µM)
MLL-7
Cell survival (%)
0 50 100
4-HPC 0 0.625 1.25 2.5 5.0 10.0 20.0
Decitabine 0 1.5 3.0 6.0 12.5 25.0 50.0
Drug concentration (µM)
MLL-14
Cell survival (%)
0 50 100
4-HPC 0 0.625 1.25 2.5 5.0 10.0 20.0
Decitabine 0 1.5 3.0 6.0 12.5 25.0 50.0
Drug concentration (µM)
LR-iALL2
Cell survival (%)
0 50 100
4-HPC 0 0.625 1.25 2.5 5.0 10.0 20.0
Decitabine 0 1.5 3.0 6.0 12.5 25.0 50.0
Drug concentration (µM)
Decitabine
4-HPC
Decitabine + 4-HPC

C

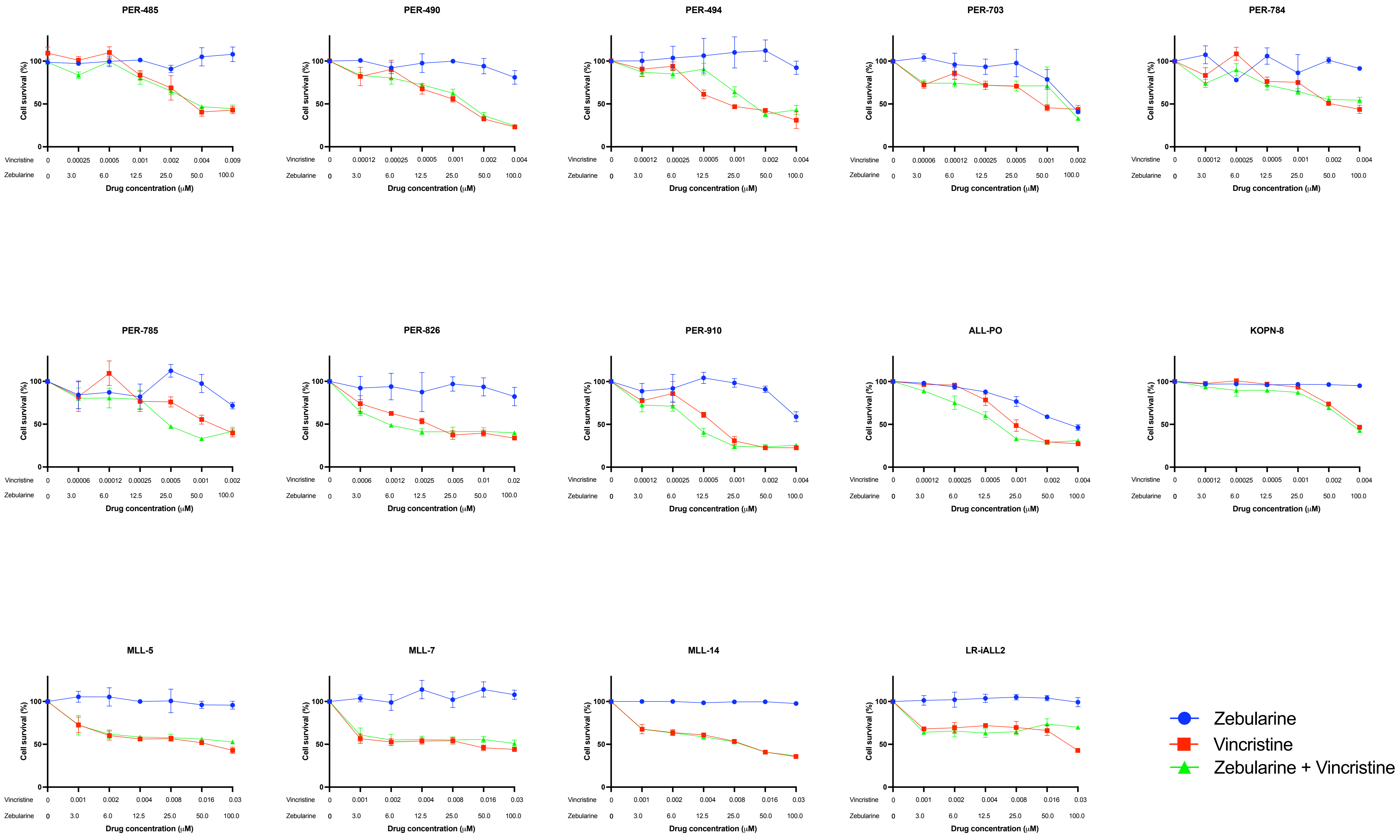

PER-485
Cell survival (%)
0
50
100
Vincristine 0 0.00025 0.0005 0.001 0.002 0.004 0.009
Zebularine 0 3.0 6.0 12.5 25.0 50.0 100.0
Drug concentration (μM)
PER-490
Cell survival (%)
Vincristine 0 0.00012 0.00025 0.0005 0.001 0.002 0.004
Zebularine 0 3.0 6.0 12.5 25.0 50.0 100.0
Drug concentration (μM)
PER-494
Cell survival (%)
Vincristine 0 0.00012 0.00025 0.0005 0.001 0.002 0.004
Zebularine 0 3.0 6.0 12.5 25.0 50.0 100.0
Drug concentration (μM)
PER-703
Cell survival (%)
Vincristine 0 0.00006 0.00012 0.00025 0.0005 0.001 0.002
Zebularine 0 3.0 6.0 12.5 25.0 50.0 100.0
Drug concentration (μM)
PER-784
Cell survival (%)
Vincristine 0 0.00012 0.00025 0.0005 0.001 0.002 0.004
Zebularine 0 3.0 6.0 12.5 25.0 50.0 100.0
Drug concentration (μM)
PER-785
Cell survival (%)
Vincristine 0 0.00006 0.00012 0.00025 0.0005 0.001 0.002
Zebularine 0 3.0 6.0 12.5 25.0 50.0 100.0
Drug concentration (μM)
PER-826
Cell survival (%)
Vincristine 0 0.0006 0.0012 0.0025 0.005 0.01 0.02
Zebularine 0 3.0 6.0 12.5 25.0 50.0 100.0
Drug concentration (μM)
PER-910
Cell survival (%)
Vincristine 0 0.00012 0.00025 0.0005 0.001 0.002 0.004
Zebularine 0 3.0 6.0 12.5 25.0 50.0 100.0
Drug concentration (μM)
ALL-PO
Cell survival (%)
Vincristine 0 0.00012 0.00025 0.0005 0.001 0.002 0.004
Zebularine 0 3.0 6.0 12.5 25.0 50.0 100.0
Drug concentration (μM)
KOPN-8
Cell survival (%)
Vincristine 0 0.00012 0.00025 0.0005 0.001 0.002 0.004
Zebularine 0 3.0 6.0 12.5 25.0 50.0 100.0
Drug concentration (μM)
MLL-5
Cell survival (%)
Vincristine 0 0.001 0.002 0.004 0.008 0.016 0.03
Zebularine 0 3.0 6.0 12.5 25.0 50.0 100.0
Drug concentration (μM)
MLL-7
Cell survival (%)
Vincristine 0 0.001 0.002 0.004 0.008 0.016 0.03
Zebularine 0 3.0 6.0 12.5 25.0 50.0 100.0
Drug concentration (μM)
MLL-14
Cell survival (%)
Vincristine 0 0.001 0.002 0.004 0.008 0.016 0.03
Zebularine 0 3.0 6.0 12.5 25.0 50.0 100.0
Drug concentration (μM)
LR-iALL2
Cell survival (%)
Vincristine 0 0.001 0.002 0.004 0.008 0.016 0.03
Zebularine 0 3.0 6.0 12.5 25.0 50.0 100.0
Drug concentration (μM)
Zebularine
Vincristine
Zebularine + Vincristine

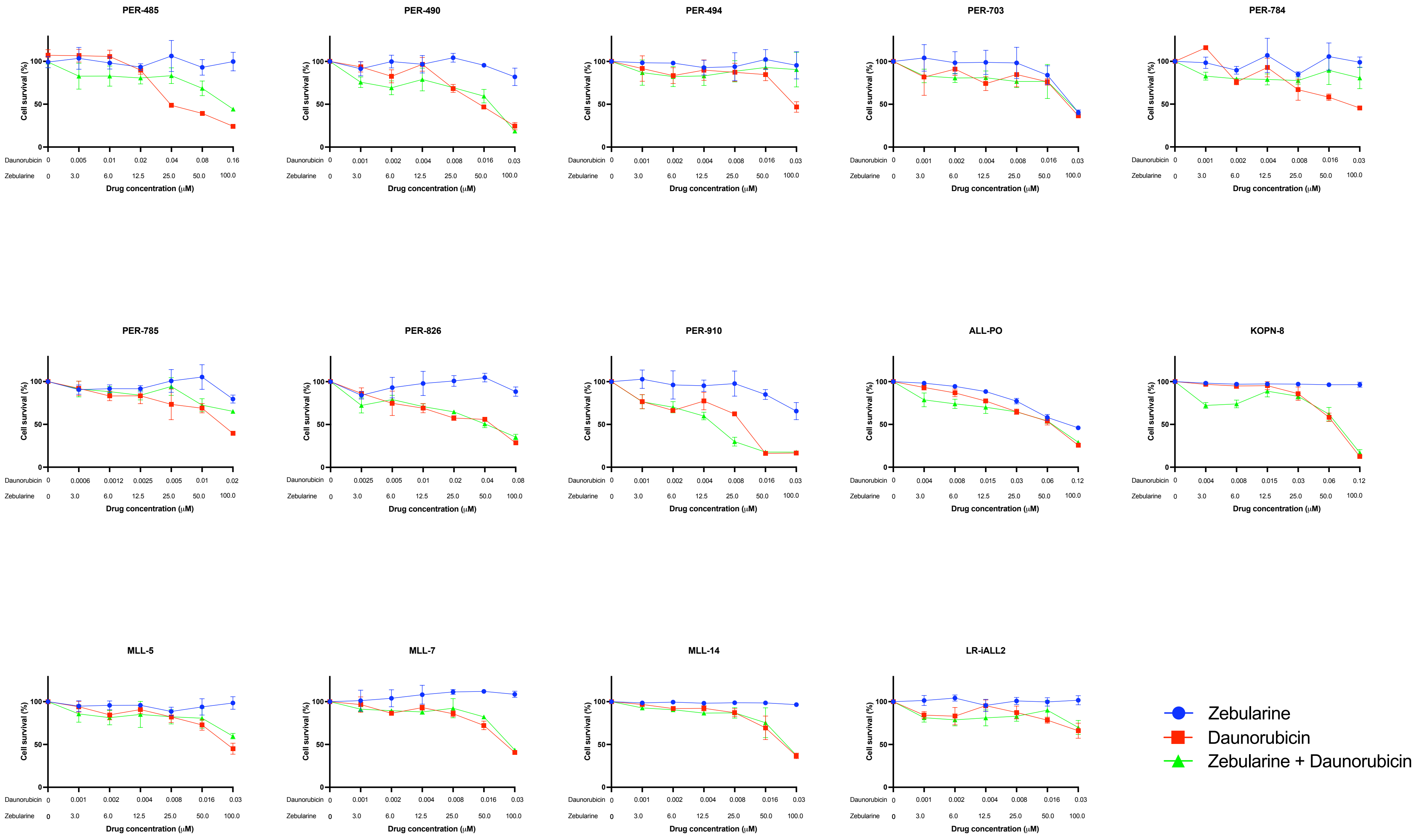
PER-485
PER-490
PER-494
PER-703
PER-784
PER-785
PER-826
PER-910
ALL-PO
KOPN-8
MLL-5
MLL-7
MLL-14
LR-iALL2
Cell survival (%)
Daunorubicin
Zebularine
Drug concentration (μM)
Zebularine
Daunorubicin
Zebularine + Daunorubicin

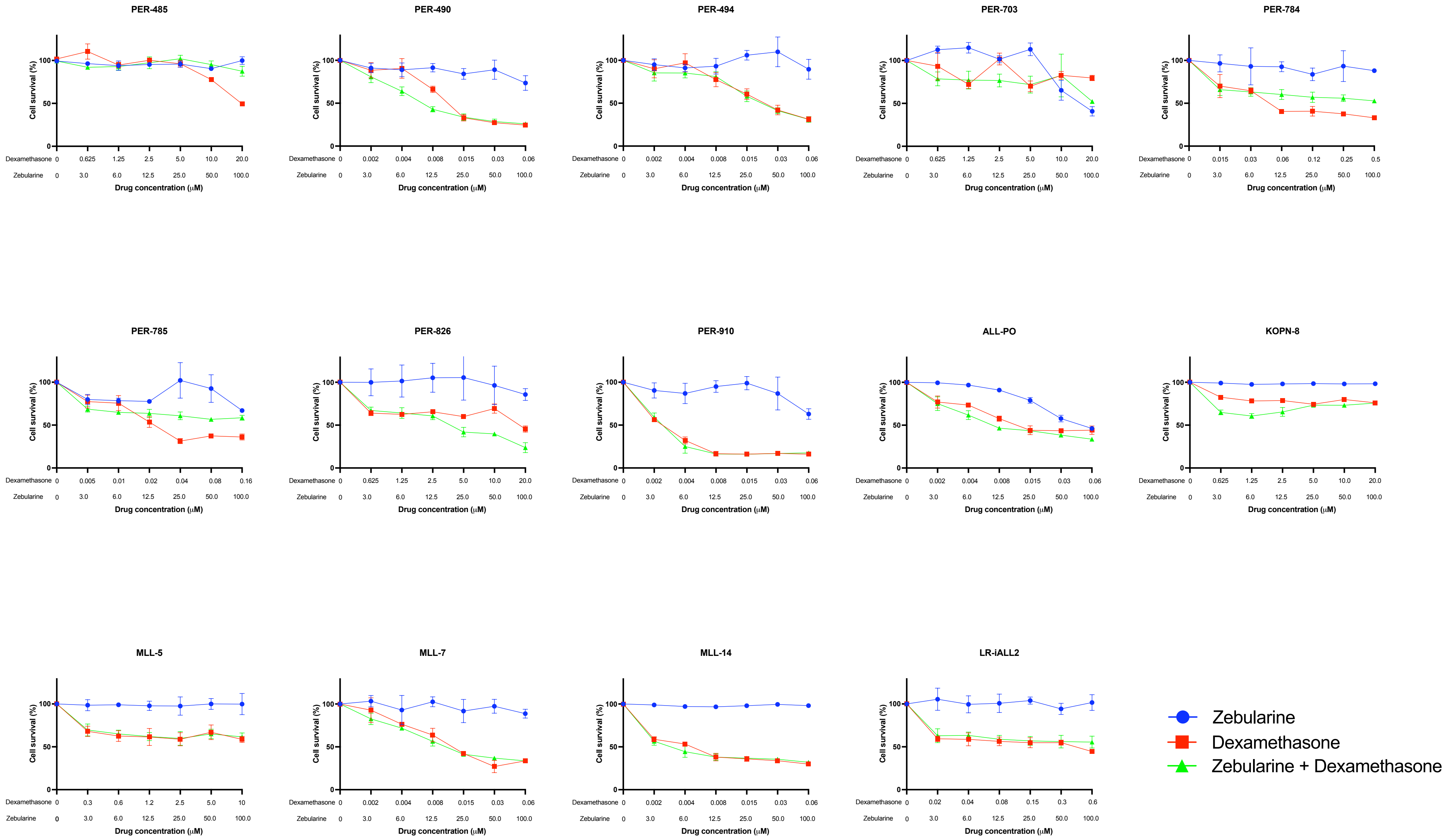
PER-485
Cell survival (%)
0
50
100
Dexamethasone 0 0.625 1.25 2.5 5.0 10.0 20.0
Zebularine 0 3.0 6.0 12.5 25.0 50.0 100.0
Drug concentration (μM)
PER-490
Dexamethasone 0 0.002 0.004 0.008 0.015 0.03 0.06
Zebularine 0 3.0 6.0 12.5 25.0 50.0 100.0
PER-494
Dexamethasone 0 0.002 0.004 0.008 0.015 0.03 0.06
Zebularine 0 3.0 6.0 12.5 25.0 50.0 100.0
PER-703
Dexamethasone 0 0.625 1.25 2.5 5.0 10.0 20.0
Zebularine 0 3.0 6.0 12.5 25.0 50.0 100.0
PER-784
Dexamethasone 0 0.015 0.03 0.06 0.12 0.25 0.5
Zebularine 0 3.0 6.0 12.5 25.0 50.0 100.0
PER-785
Dexamethasone 0 0.005 0.01 0.02 0.04 0.08 0.16
Zebularine 0 3.0 6.0 12.5 25.0 50.0 100.0
PER-826
Dexamethasone 0 0.625 1.25 2.5 5.0 10.0 20.0
Zebularine 0 3.0 6.0 12.5 25.0 50.0 100.0
PER-910
Dexamethasone 0 0.002 0.004 0.008 0.015 0.03 0.06
Zebularine 0 3.0 6.0 12.5 25.0 50.0 100.0
ALL-PO
Dexamethasone 0 0.002 0.004 0.008 0.015 0.03 0.06
Zebularine 0 3.0 6.0 12.5 25.0 50.0 100.0
KOPN-8
Dexamethasone 0 0.625 1.25 2.5 5.0 10.0 20.0
Zebularine 0 3.0 6.0 12.5 25.0 50.0 100.0
MLL-5
Dexamethasone 0 0.3 0.6 1.2 2.5 5.0 10
Zebularine 0 3.0 6.0 12.5 25.0 50.0 100.0
MLL-7
Dexamethasone 0 0.002 0.004 0.008 0.015 0.03 0.06
Zebularine 0 3.0 6.0 12.5 25.0 50.0 100.0
MLL-14
Dexamethasone 0 0.002 0.004 0.008 0.015 0.03 0.06
Zebularine 0 3.0 6.0 12.5 25.0 50.0 100.0
LR-iALL2
Dexamethasone 0 0.02 0.04 0.08 0.15 0.3 0.6
Zebularine 0 3.0 6.0 12.5 25.0 50.0 100.0
Zebularine
Dexamethasone
Zebularine + Dexamethasone

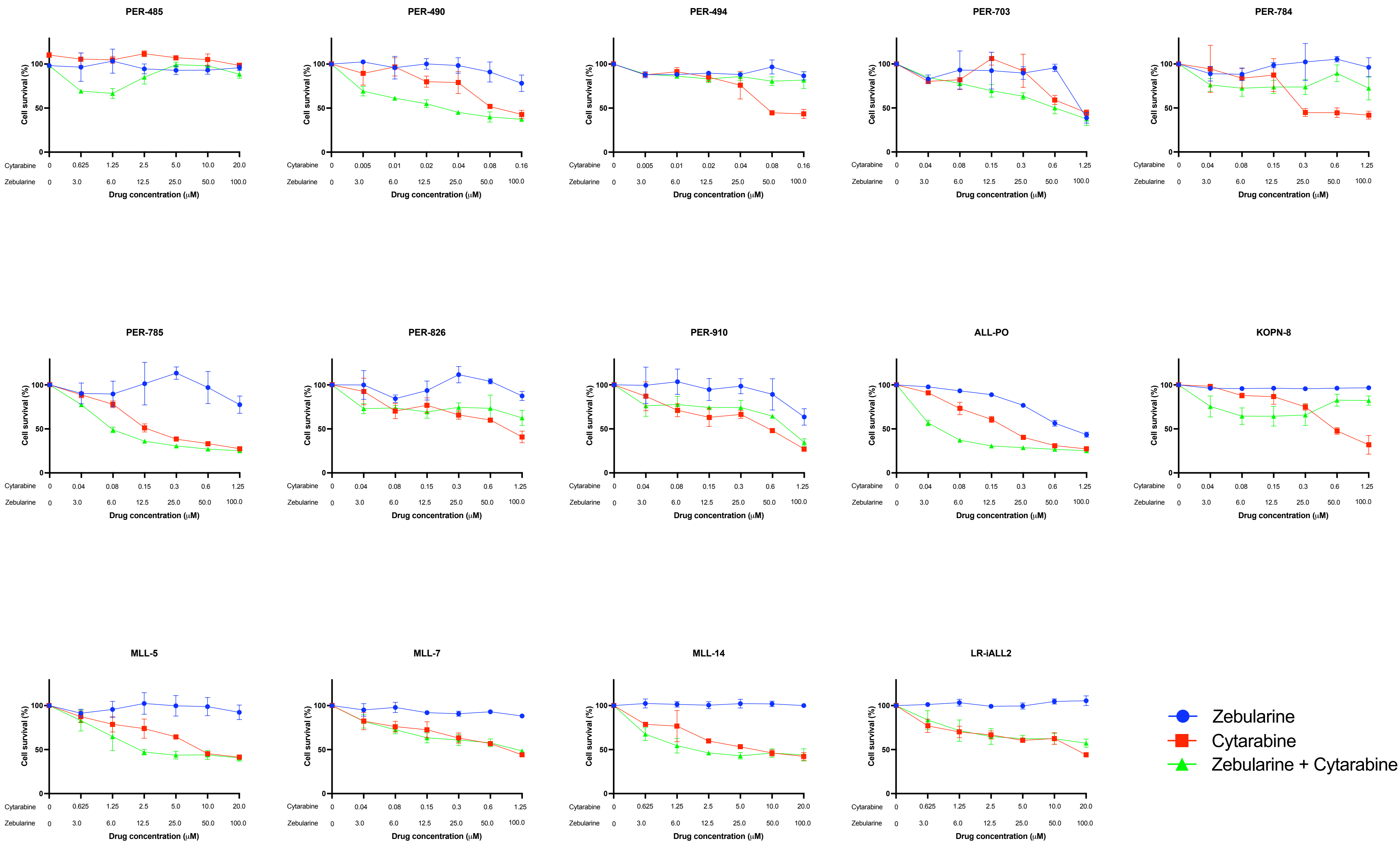
PER-485
Cell survival (%)
0
50
100
Cytarabine 0 0.625 1.25 2.5 5.0 10.0 20.0
Zebularine 0 3.0 6.0 12.5 25.0 50.0 100.0
Drug concentration (μM)
PER-490
Cell survival (%)
Cytarabine 0 0.005 0.01 0.02 0.04 0.08 0.16
Zebularine 0 3.0 6.0 12.5 25.0 50.0 100.0
Drug concentration (μM)
PER-494
Cell survival (%)
Cytarabine 0 0.005 0.01 0.02 0.04 0.08 0.16
Zebularine 0 3.0 6.0 12.5 25.0 50.0 100.0
Drug concentration (μM)
PER-703
Cell survival (%)
Cytarabine 0 0.04 0.08 0.15 0.3 0.6 1.25
Zebularine 0 3.0 6.0 12.5 25.0 50.0 100.0
Drug concentration (μM)
PER-784
Cell survival (%)
Cytarabine 0 0.04 0.08 0.15 0.3 0.6 1.25
Zebularine 0 3.0 6.0 12.5 25.0 50.0 100.0
Drug concentration (μM)
PER-785
Cell survival (%)
Cytarabine 0 0.04 0.08 0.15 0.3 0.6 1.25
Zebularine 0 3.0 6.0 12.5 25.0 50.0 100.0
Drug concentration (μM)
PER-826
Cell survival (%)
Cytarabine 0 0.04 0.08 0.15 0.3 0.6 1.25
Zebularine 0 3.0 6.0 12.5 25.0 50.0 100.0
Drug concentration (μM)
PER-910
Cell survival (%)
Cytarabine 0 0.04 0.08 0.15 0.3 0.6 1.25
Zebularine 0 3.0 6.0 12.5 25.0 50.0 100.0
Drug concentration (μM)
ALL-PO
Cell survival (%)
Cytarabine 0 0.04 0.08 0.15 0.3 0.6 1.25
Zebularine 0 3.0 6.0 12.5 25.0 50.0 100.0
Drug concentration (μM)
KOPN-8
Cell survival (%)
Cytarabine 0 0.04 0.08 0.15 0.3 0.6 1.25
Zebularine 0 3.0 6.0 12.5 25.0 50.0 100.0
Drug concentration (μM)
MLL-5
Cell survival (%)
Cytarabine 0 0.625 1.25 2.5 5.0 10.0 20.0
Zebularine 0 3.0 6.0 12.5 25.0 50.0 100.0
Drug concentration (μM)
MLL-7
Cell survival (%)
Cytarabine 0 0.04 0.08 0.15 0.3 0.6 1.25
Zebularine 0 3.0 6.0 12.5 25.0 50.0 100.0
Drug concentration (μM)
MLL-14
Cell survival (%)
Cytarabine 0 0.625 1.25 2.5 5.0 10.0 20.0
Zebularine 0 3.0 6.0 12.5 25.0 50.0 100.0
Drug concentration (μM)
LR-iALL2
Cell survival (%)
Cytarabine 0 0.625 1.25 2.5 5.0 10.0 20.0
Zebularine 0 3.0 6.0 12.5 25.0 50.0 100.0
Drug concentration (μM)
Zebularine
Cytarabine
Zebularine + Cytarabine

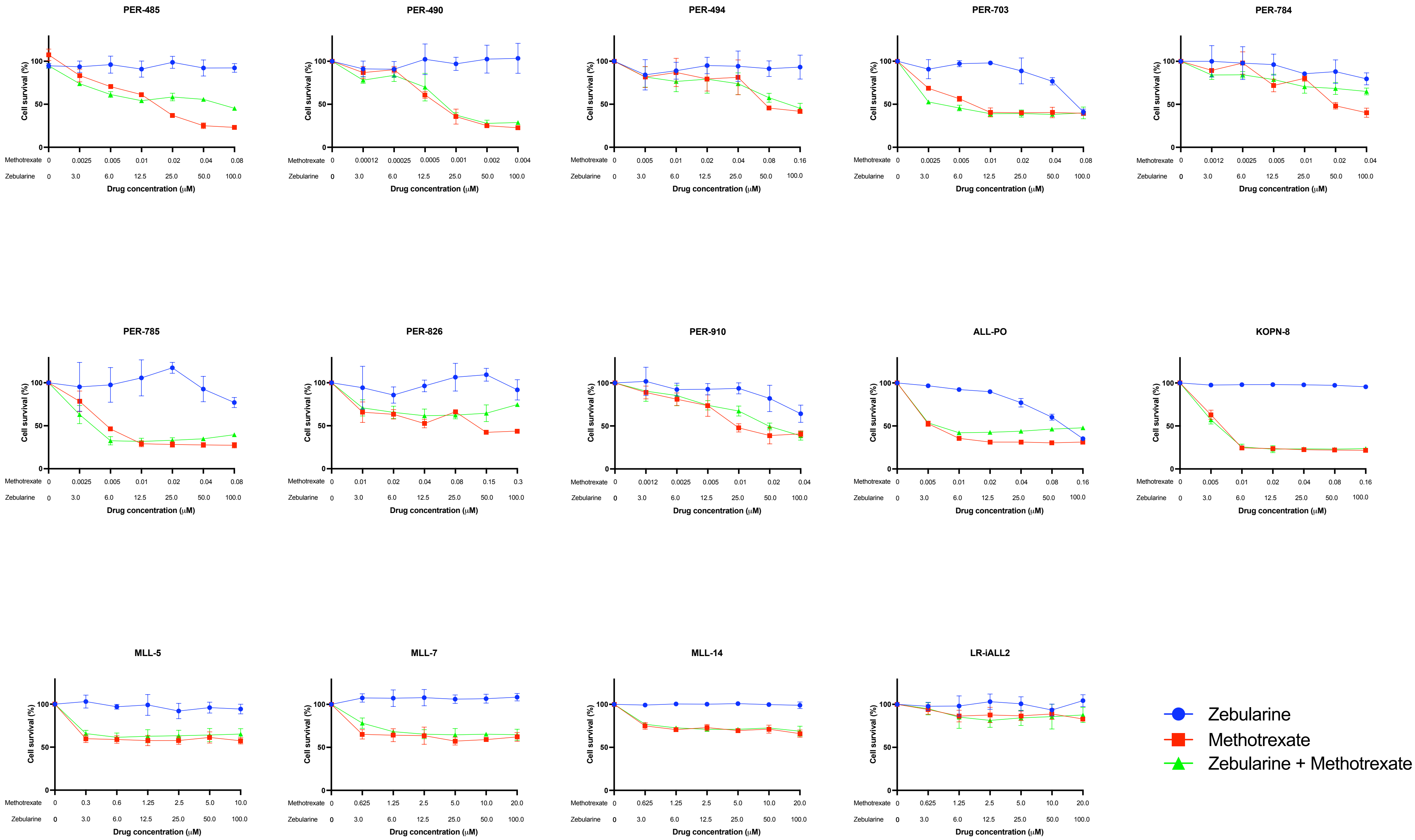
PER-485
Cell survival (%)
0 50 100
Methotrexate 0 0.0025 0.005 0.01 0.02 0.04 0.08
Zebularine 0 3.0 6.0 12.5 25.0 50.0 100.0
Drug concentration (μM)
PER-490
Methotrexate 0 0.00012 0.00025 0.0005 0.001 0.002 0.004
Zebularine 0 3.0 6.0 12.5 25.0 50.0 100.0
PER-494
Methotrexate 0 0.005 0.01 0.02 0.04 0.08 0.16
Zebularine 0 3.0 6.0 12.5 25.0 50.0 100.0
PER-703
Methotrexate 0 0.0025 0.005 0.01 0.02 0.04 0.08
Zebularine 0 3.0 6.0 12.5 25.0 50.0 100.0
PER-784
Methotrexate 0 0.0012 0.0025 0.005 0.01 0.02 0.04
Zebularine 0 3.0 6.0 12.5 25.0 50.0 100.0
PER-785
Methotrexate 0 0.0025 0.005 0.01 0.02 0.04 0.08
Zebularine 0 3.0 6.0 12.5 25.0 50.0 100.0
PER-826
Methotrexate 0 0.01 0.02 0.04 0.08 0.15 0.3
Zebularine 0 3.0 6.0 12.5 25.0 50.0 100.0
PER-910
Methotrexate 0 0.0012 0.0025 0.005 0.01 0.02 0.04
Zebularine 0 3.0 6.0 12.5 25.0 50.0 100.0
ALL-PO
Methotrexate 0 0.005 0.01 0.02 0.04 0.08 0.16
Zebularine 0 3.0 6.0 12.5 25.0 50.0 100.0
KOPN-8
Methotrexate 0 0.005 0.01 0.02 0.04 0.08 0.16
Zebularine 0 3.0 6.0 12.5 25.0 50.0 100.0
MLL-5
Methotrexate 0 0.3 0.6 1.25 2.5 5.0 10.0
Zebularine 0 3.0 6.0 12.5 25.0 50.0 100.0
MLL-7
Methotrexate 0 0.625 1.25 2.5 5.0 10.0 20.0
Zebularine 0 3.0 6.0 12.5 25.0 50.0 100.0
MLL-14
Methotrexate 0 0.625 1.25 2.5 5.0 10.0 20.0
Zebularine 0 3.0 6.0 12.5 25.0 50.0 100.0
LR-iALL2
Methotrexate 0 0.625 1.25 2.5 5.0 10.0 20.0
Zebularine 0 3.0 6.0 12.5 25.0 50.0 100.0
Zebularine
Methotrexate
Zebularine + Methotrexate

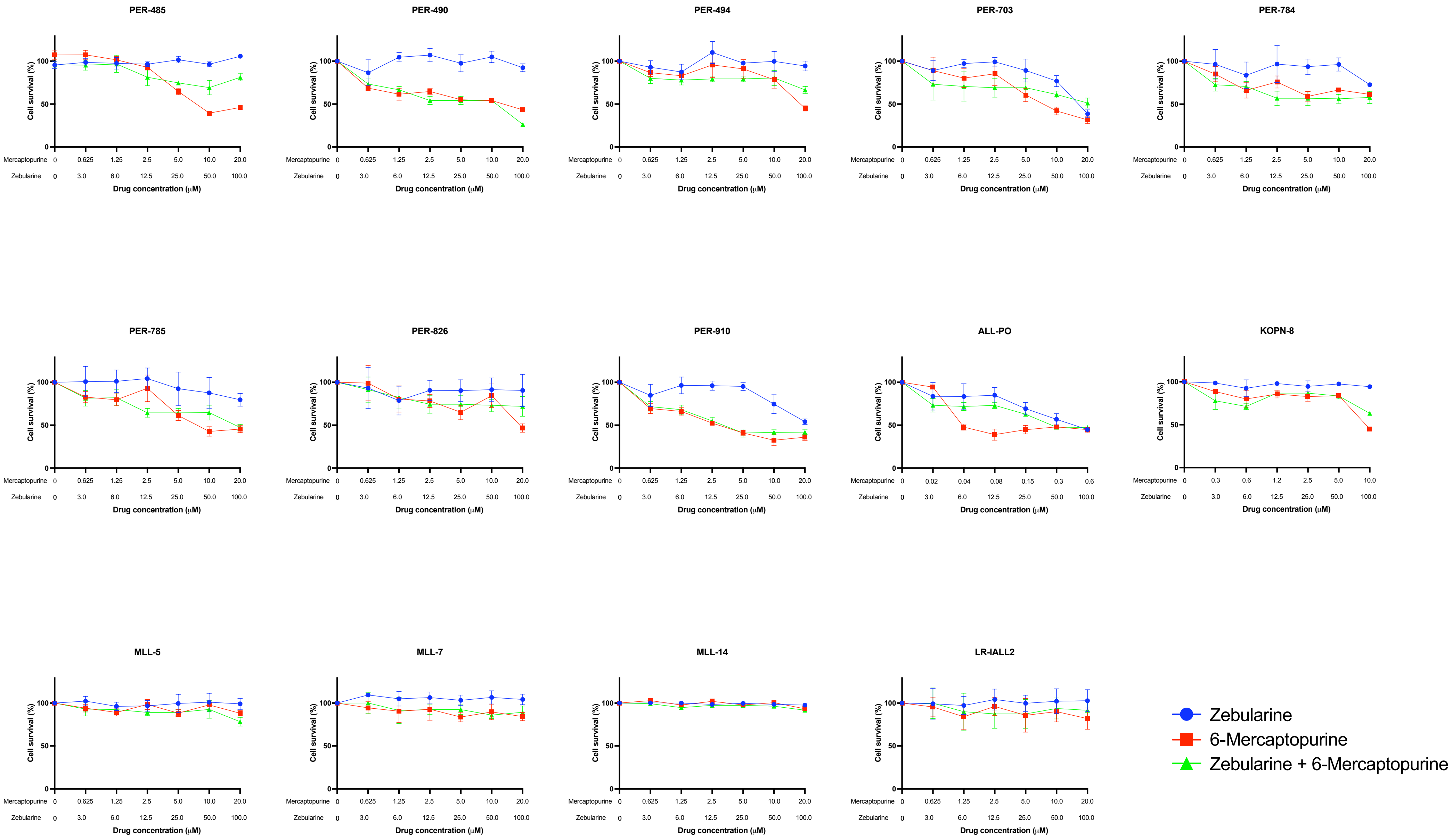

PER-485
PER-490
PER-494
PER-703
PER-784
PER-785
PER-826
PER-910
ALL-PO
KOPN-8
MLL-5
MLL-7
MLL-14
LR-iALL2
Cell survival (%)
0
50
100
Mercaptopurine 0 0.625 1.25 2.5 5.0 10.0 20.0
Zebularine 0 3.0 6.0 12.5 25.0 50.0 100.0
Mercaptopurine 0 0.02 0.04 0.08 0.15 0.3 0.6
Mercaptopurine 0 0.3 0.6 1.2 2.5 5.0 10.0
Drug concentration (μM)
Zebularine
6-Mercaptopurine
Zebularine + 6-Mercaptopurine

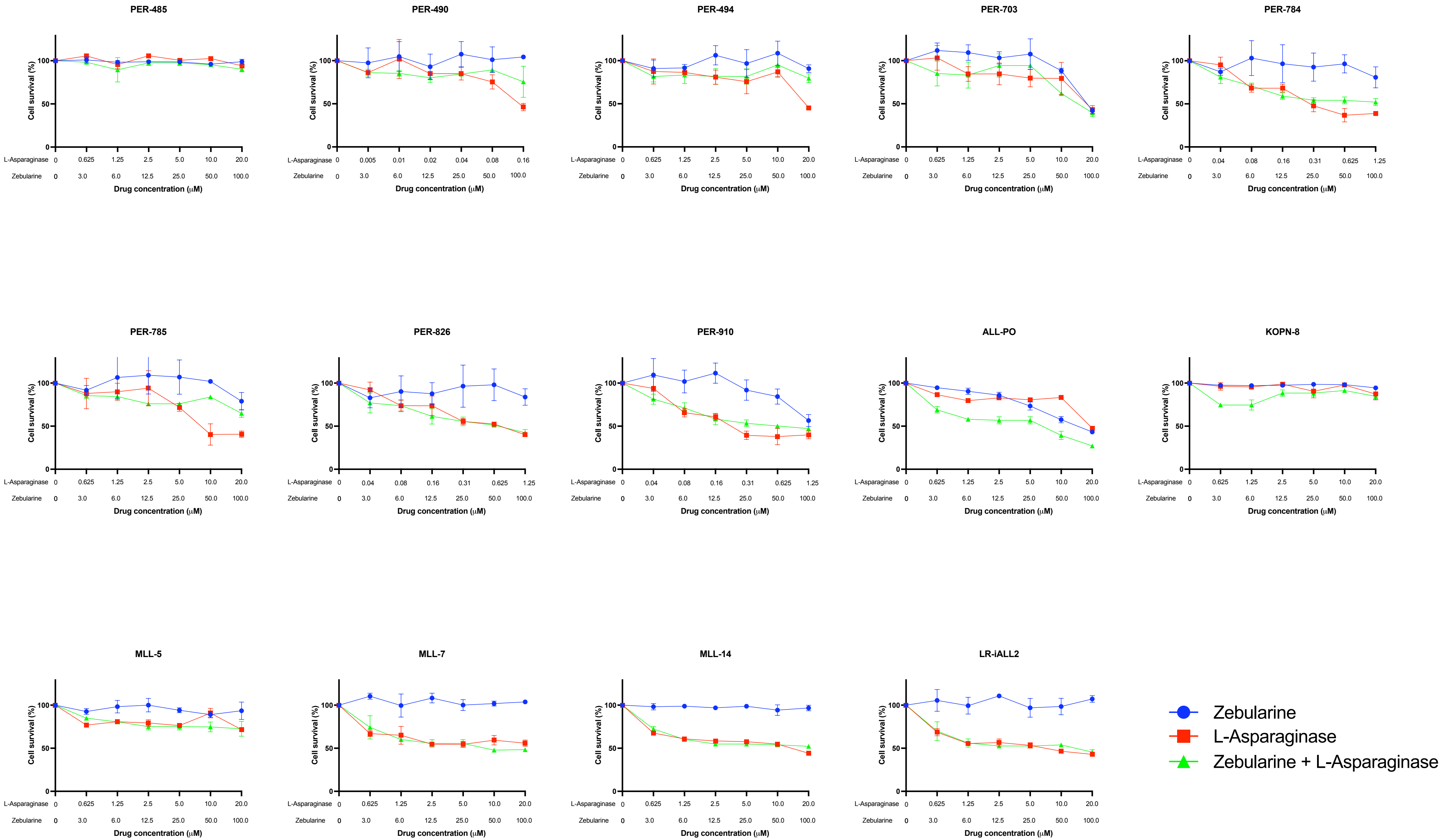
PER-485
PER-490
PER-494
PER-703
PER-784
PER-785
PER-826
PER-910
ALL-PO
KOPN-8
MLL-5
MLL-7
MLL-14
LR-iALL2
Cell survival (%)
L-Asparaginase
Zebularine
Drug concentration (μM)
Zebularine
L-Asparaginase
Zebularine + L-Asparaginase

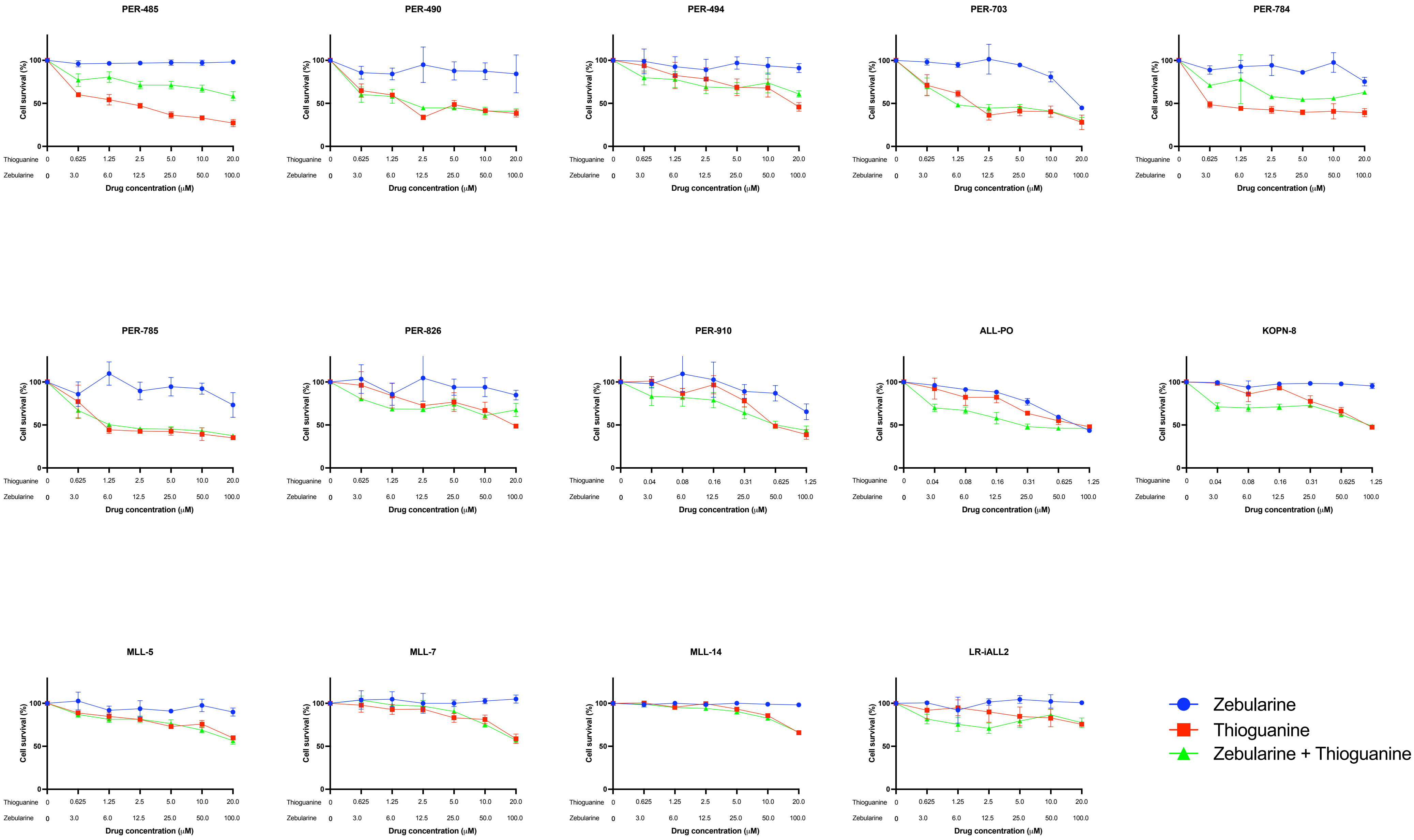
PER-485
PER-490
PER-494
PER-703
PER-784
PER-785
PER-826
PER-910
ALL-PO
KOPN-8
MLL-5
MLL-7
MLL-14
LR-iALL2
Cell survival (%)
0
50
100
Thioguanine 0 0.625 1.25 2.5 5.0 10.0 20.0
Zebularine 0 3.0 6.0 12.5 25.0 50.0 100.0
Thioguanine 0 0.04 0.08 0.16 0.31 0.625 1.25
Zebularine 0 3.0 6.0 12.5 25.0 50.0 100.0
Drug concentration (μM)
Zebularine
Thioguanine
Zebularine + Thioguanine

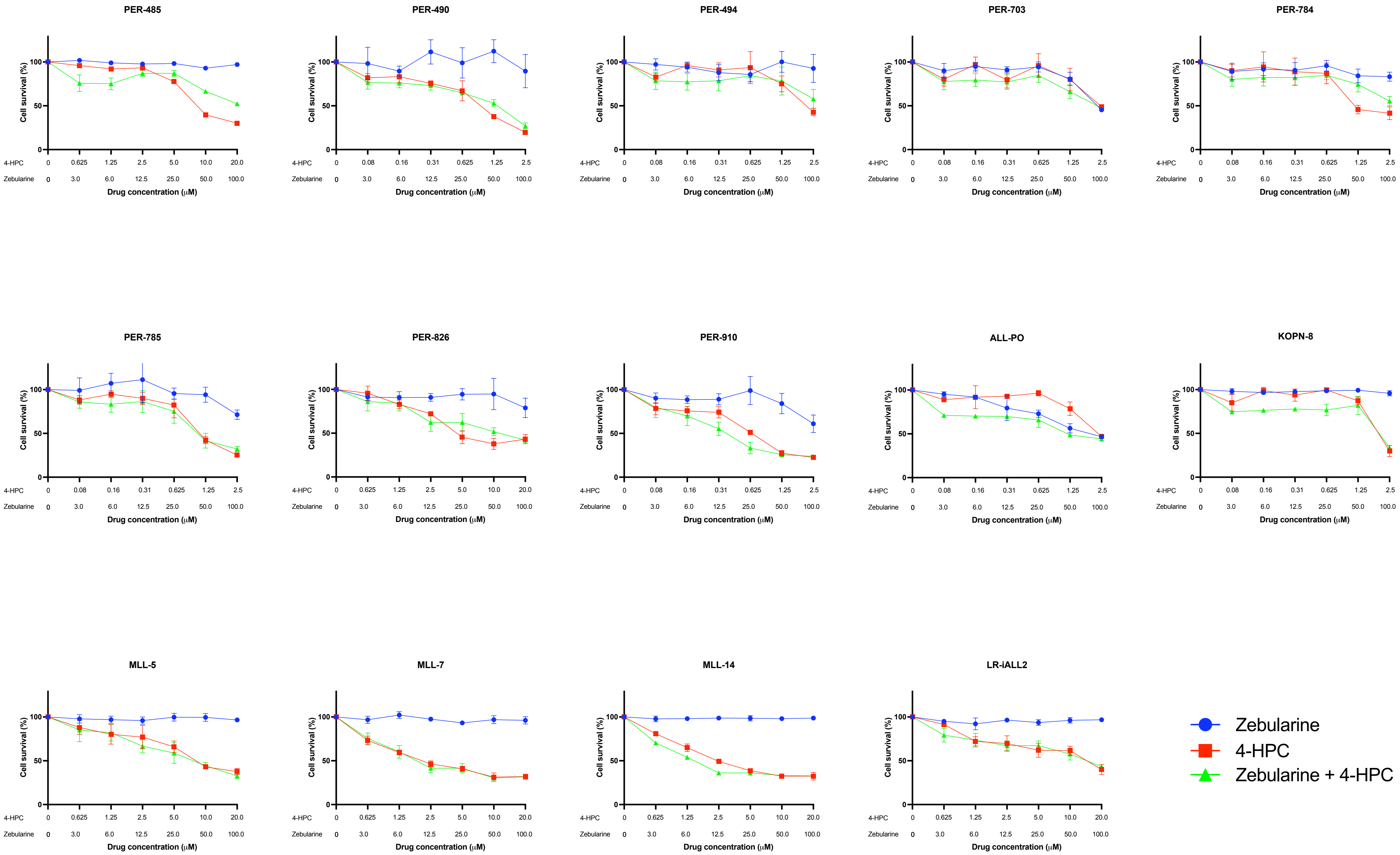
PER-485
PER-490
PER-494
PER-703
PER-784
PER-785
PER-826
PER-910
ALL-PO
KOPN-8
MLL-5
MLL-7
MLL-14
LR-iALL2
Cell survival (%)
0
50
100
4-HPC 0 0.625 1.25 2.5 5.0 10.0 20.0
4-HPC 0 0.08 0.16 0.31 0.625 1.25 2.5
Zebularine 0 3.0 6.0 12.5 25.0 50.0 100.0
Drug concentration (µM)
Zebularine
4-HPC
Zebularine + 4-HPC

Supplemental Figure S5. Antagonistic landscape of methotrexate in combination with (A) azacitidine or (B) decitabine in three *KMT2A*-rearranged infant acute lymphoblastic leukemia cell lines. Positive Bliss synergy scores indicate synergy and negative Bliss synergy scores indicate antagonism. Representative plots from three biological replicates shown.

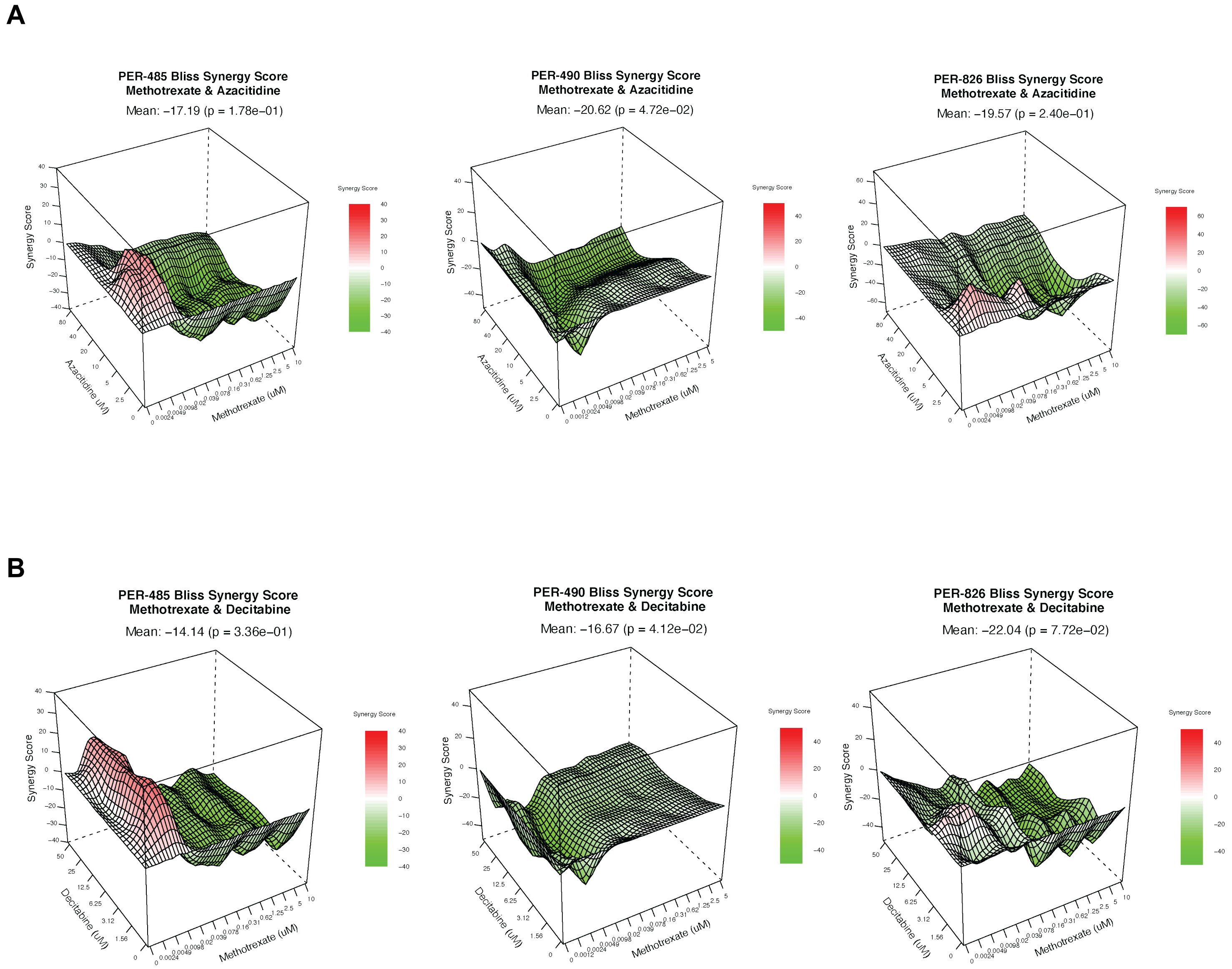

Supplemental Figure S6. (A) Manhattan plot showing differential methylation profile across genome-wide CpG sites of six *KMT2A*-rearranged infant acute lymphoblastic leukemia (ALL) cell lines treated with low dose (6.0µM) zebularine in comparison to untreated controls. There were no differentially methylated sites that reached the genome-wide significance threshold of 5×10^{-8}. The blue line indicates suggestive significance, valued at 1×10^{-5}. (B) Heatmaps featuring the expression levels (average log counts per million) of the most significant differentially expressed genes following treatment with azacitidine and decitabine across all six cell lines in contrast to untreated controls. (C) Volcano plots displaying the top differentially expressed genes from the transcriptome of the six cell lines following treatment with azacitidine and decitabine. Horizontal and vertical dashed lines indicate fold change and significance thresholds. (D) Heatmap displaying the normalized enrichment scores (NES) from gene set enrichment analyses performed on ranked differentially expressed genes following treatment with decitabine. NES are shown for each of the six cell lines and after merging the transcriptome of all six cell lines. Positive (red) and negative (blue) NES scores indicate enrichment of a specific gene set following treatment and in untreated controls, respectively. Gene sets with a false discovery rate <0.1 were considered significant. (E) Scatter plot comparing the mean beta fold change values, indicating hypomethylation (x axis), and $\log_2$ fold change of the expression levels (y axis) for genes within the 617 hypomethylated regions following treatment with azacitidine and for genes within the 471 hypomethylated regions following treatment with decitabine. Fold changes for each gene were extracted from the merged comparison of all cell lines treated with azacitidine or decitabine against untreated controls. The mean beta fold change values indicate hypomethylation level (<-0.1). Genes with $\log_2$ fold change higher than 1 (dashed line) and false discovery rate <0.05 (red dots) are labelled. (F) Expression levels ($\log_2$ fold change) of *MMP15* , *BAIAP3* and *CD82* measured by real-time quantitative PCR, in an extended cohort of 13 *KMT2A*-rearranged infant ALL *in vitro* models after treatment with azacitidine and decitabine. Mean expression values of three biological replicates with standard error of the mean are displayed for each model. * p<0.05, ** p<0.01, *** p<0.001.

A

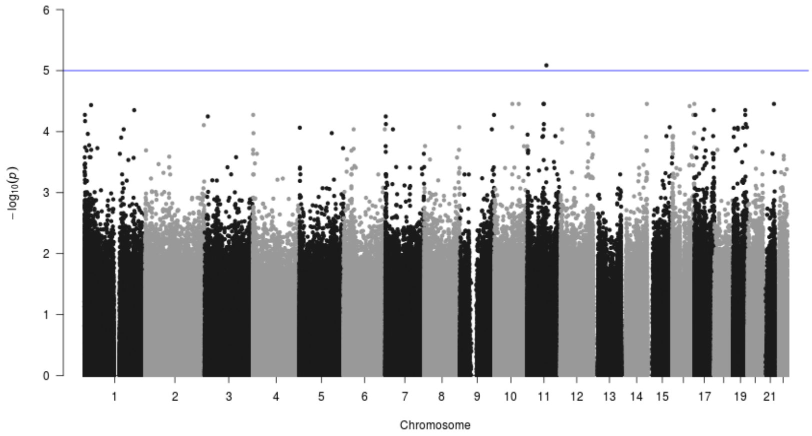

B

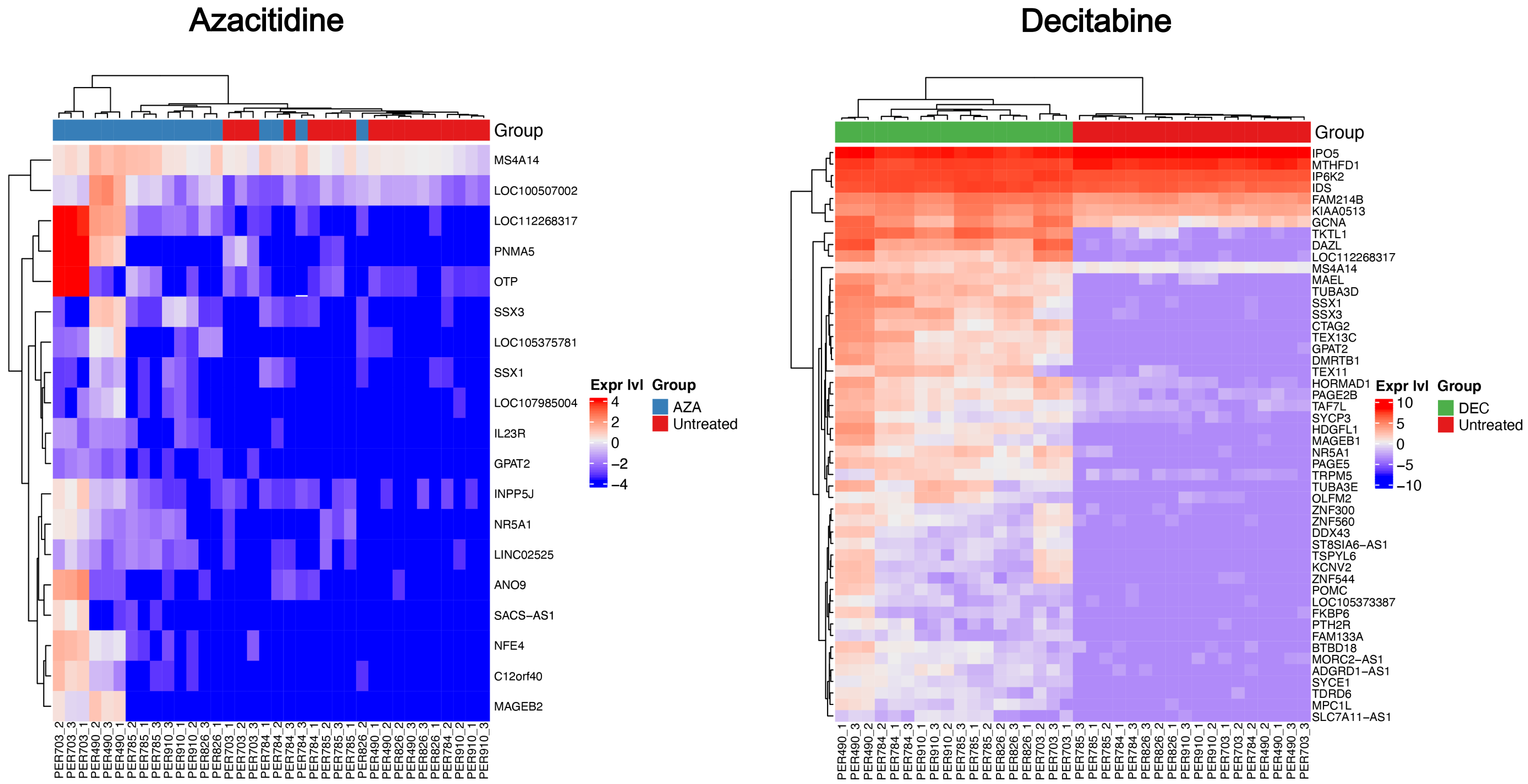

C

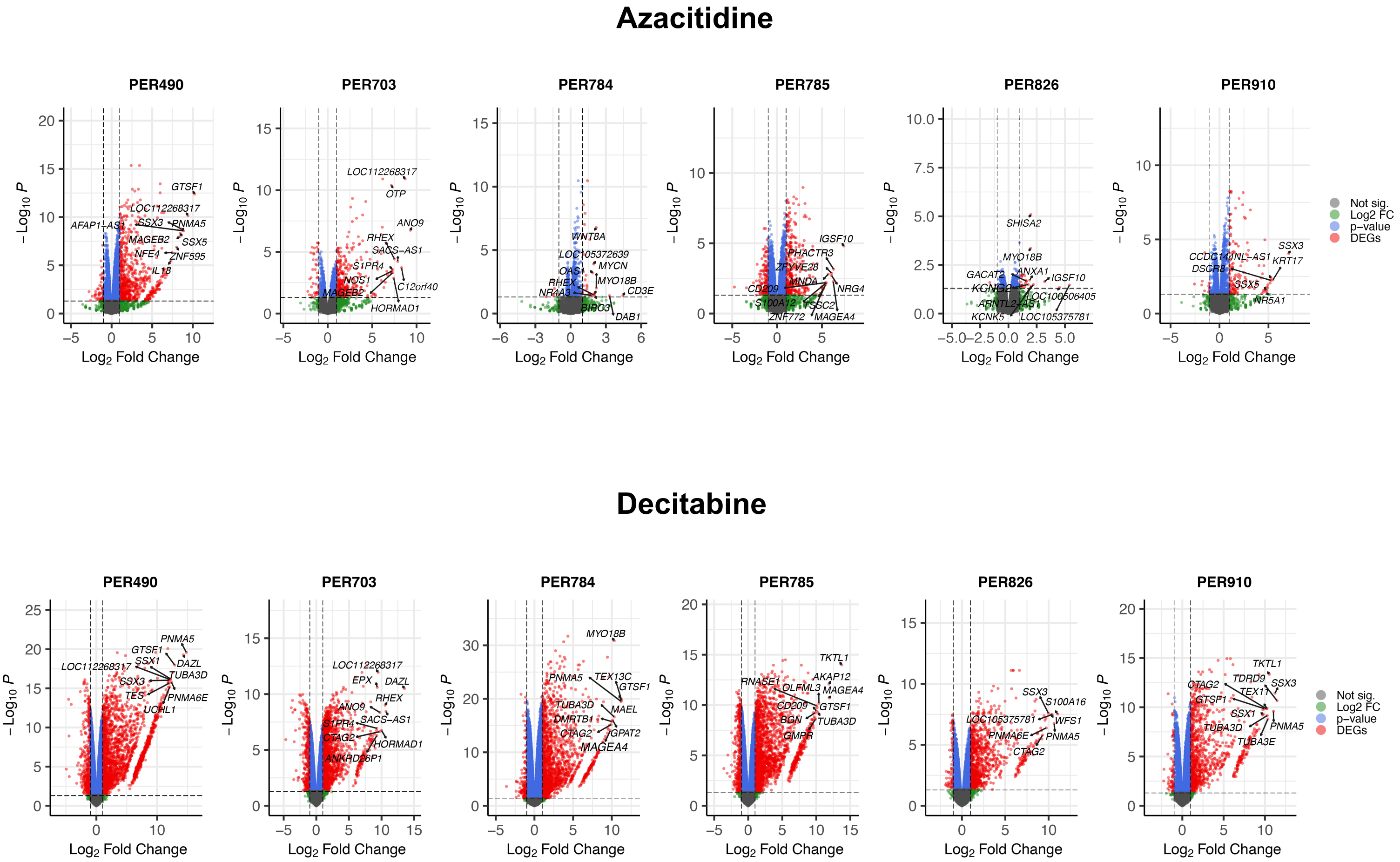
Azacitidine
PER490
PER703
PER784
PER785
PER826
PER910
$-\log_{10} P$
$\log_2$ Fold Change
Not sig.
Log2 FC
p-value
DEGs
Decitabine
PER490
PER703
PER784
PER785
PER826
PER910
$-\log_{10} P$
$\log_2$ Fold Change
Not sig.
Log2 FC
p-value
DEGs

D

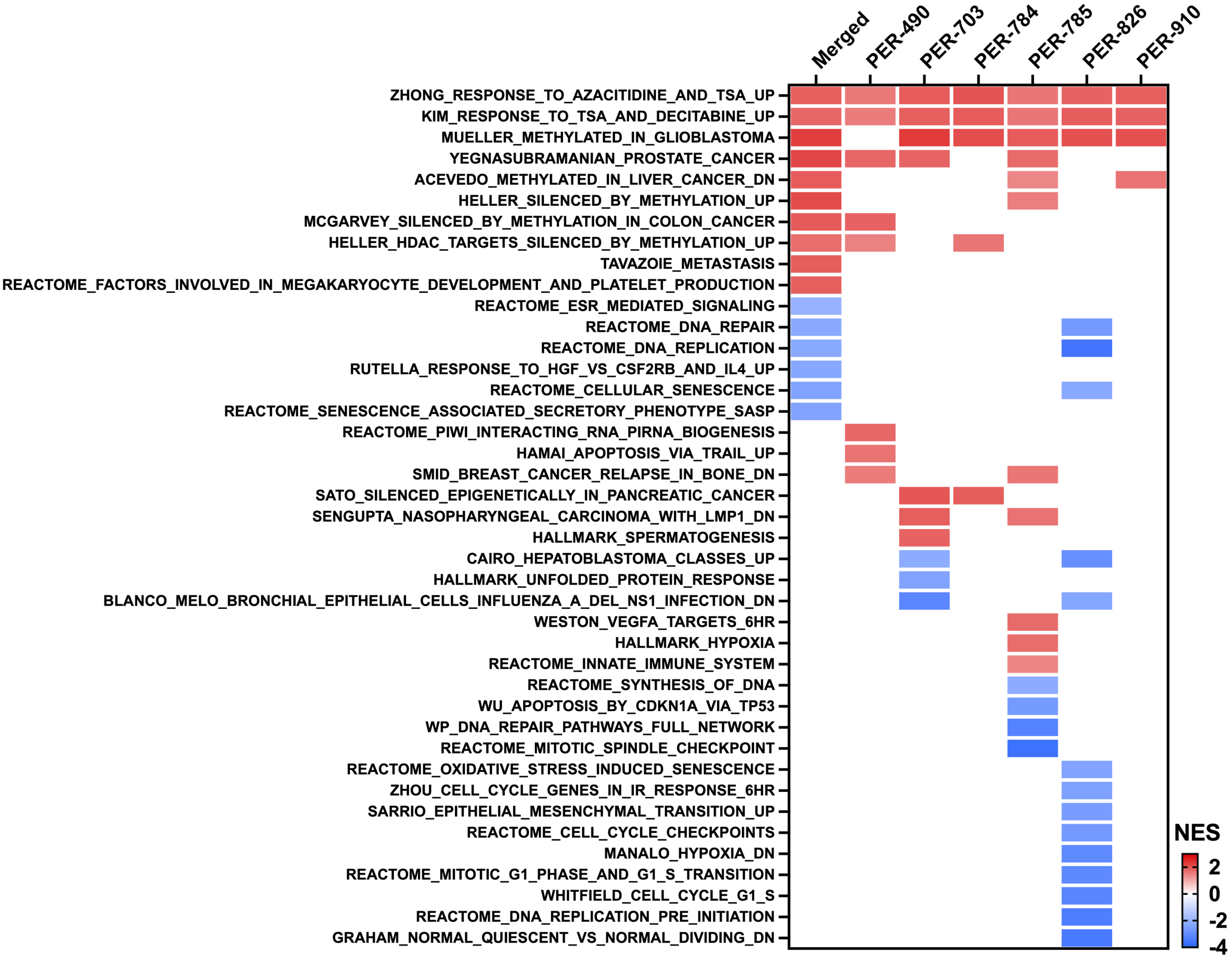
Merged
PER-490
PER-703
PER-784
PER-785
PER-826
PER-910
ZHONG_RESPONSE_TO_AZACITIDINE_AND_TSA_UP
KIM_RESPONSE_TO_TSA_AND_DECITABINE_UP
MUELLER_METHYLATED_IN_GLIOBLASTOMA
YEGNASUBRAMANIAN_PROSTATE_CANCER
ACEVEDO_METHYLATED_IN_LIVER_CANCER_DN
HELLER_SILENCED_BY_METHYLATION_UP
MCGARVEY_SILENCED_BY_METHYLATION_IN_COLON_CANCER
HELLER_HDAC_TARGETS_SILENCED_BY_METHYLATION_UP
TAVAZOIE_METASTASIS
REACTOME_FACTORS_INVOLVED_IN_MEGAKARYOCYTE_DEVELOPMENT_AND_PLATELET_PRODUCTION
REACTOME_ESR_MEDIATED_SIGNALING
REACTOME_DNA_REPAIR
REACTOME_DNA_REPLICATION
RUTELLA_RESPONSE_TO_HGF_VS_CSF2RB_AND_IL4_UP
REACTOME_CELLULAR_SENESCENCE
REACTOME_SENESCENCE_ASSOCIATED_SECRETORY_PHENOTYPE_SASP
REACTOME_PIWI_INTERACTING_RNA_PIRNA_BIOGENESIS
HAMAI_APOPTOSIS_VIA_TRAIL_UP
SMID_BREAST_CANCER_RELAPSE_IN_BONE_DN
SATO_SILENCED_EPIGENETICALLY_IN_PANCREATIC_CANCER
SENGUPTA_NASOPHARYNGEAL_CARCINOMA_WITH_LMP1_DN
HALLMARK_SPERMATOGENESIS
CAIRO_HEPATOBLASTOMA_CLASSES_UP
HALLMARK_UNFOLDED_PROTEIN_RESPONSE
BLANCO_MELO_BRONCHIAL_EPITHELIAL_CELLS_INFLUENZA_A_DEL_NS1_INFECTION_DN
WESTON_VEGFA_TARGETS_6HR
HALLMARK_HYPOXIA
REACTOME_INNATE_IMMUNE_SYSTEM
REACTOME_SYNTHESIS_OF_DNA
WU_APOPTOSIS_BY_CDKN1A_VIA_TP53
WP_DNA_REPAIR_PATHWAYS_FULL_NETWORK
REACTOME_MITOTIC_SPINDLE_CHECKPOINT
REACTOME_OXIDATIVE_STRESS_INDUCED_SENESCENCE
ZHOU_CELL_CYCLE_GENES_IN_IR_RESPONSE_6HR
SARRIO_EPITHELIAL_MESENCHYMAL_TRANSITION_UP
REACTOME_CELL_CYCLE_CHECKPOINTS
MANALO_HYPOXIA_DN
REACTOME_MITOTIC_G1_PHASE_AND_G1_S_TRANSITION
WHITFIELD_CELL_CYCLE_G1_S
REACTOME_DNA_REPLICATION_PRE_INITIATION
GRAHAM_NORMAL_QUIESCENT_VS_NORMAL_DIVIDING_DN
NES
2
0
-2
-4

E

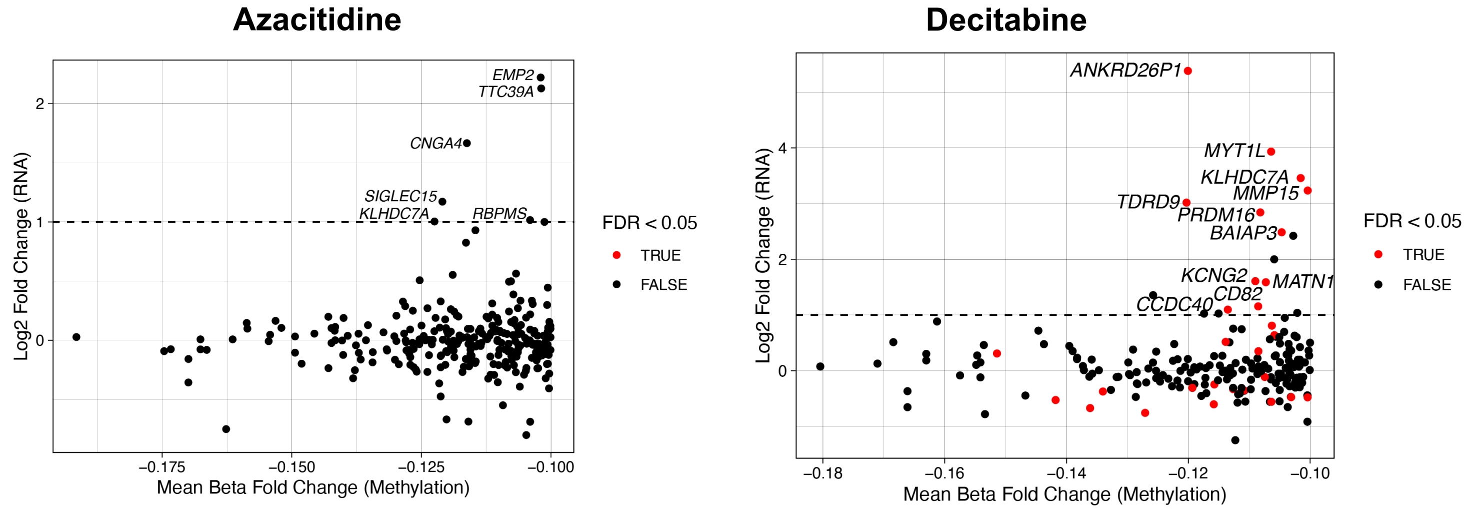

Azacitidine
Log2 Fold Change (RNA)
Mean Beta Fold Change (Methylation)
−0.175
−0.150
−0.125
−0.100
EMP2
TTC39A
CNGA4
SIGLEC15
KLHDC7A
RBPMS
Decitabine
ANKRD26P1
MYT1L
KLHDC7A
MMP15
TDRD9
PRDM16
BAIAP3
KCNG2
MATN1
CD82
CCDC40
−0.18
−0.16
−0.14
−0.12
−0.10
FDR < 0.05
TRUE
FALSE

F

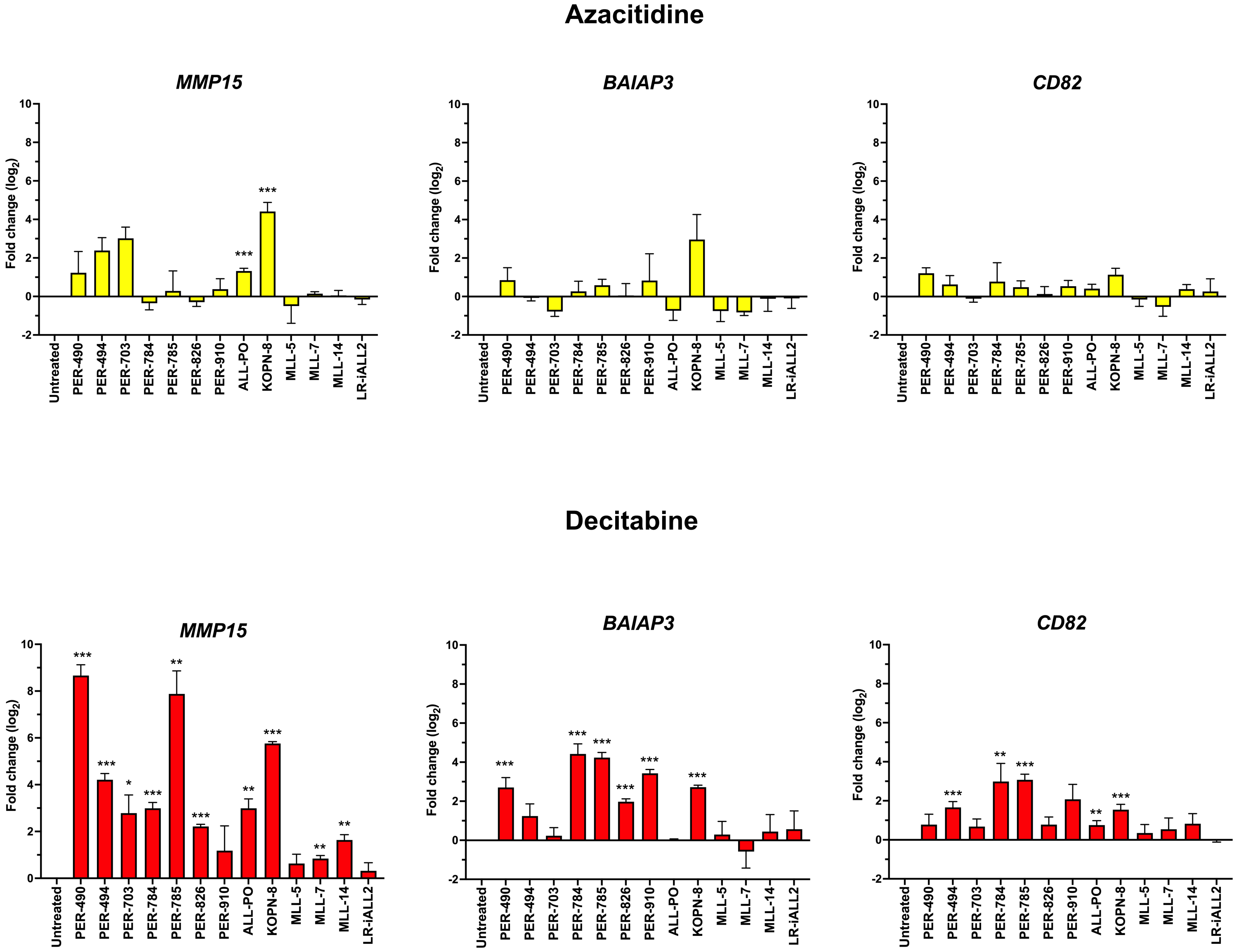

Azacitidine
MMP15
BAIAP3
CD82
Decitabine
MMP15
BAIAP3
CD82
Fold change (log2)
Untreated
PER-490
PER-494
PER-703
PER-784
PER-785
PER-826
PER-910
ALL-PO
KOPN-8
MLL-5
MLL-7
MLL-14
LR-iALL2

Supplemental Figure S7. Thermal proteome profiling of azacitidine and decitabine reveals target protein engagement. Melt curves showing changes in aggregation temperature for selected proteins in (A) azacitidine and (B) decitabine treated ALL-PO cells. (C) Melt curves showing changes in aggregation temperature for selected overlapping proteins in both azacitidine and decitabine treated ALL-PO cells.

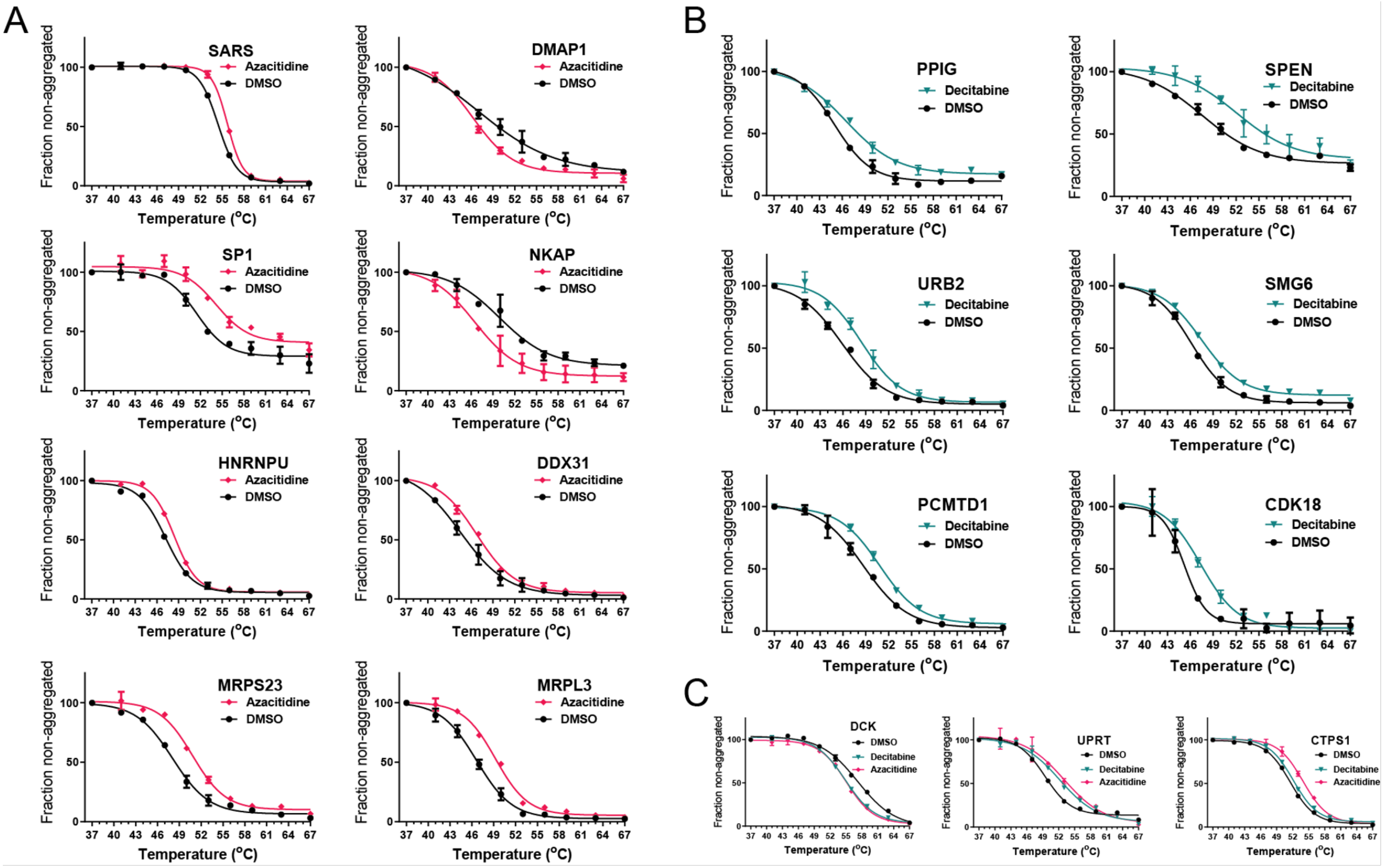

Supplemental Figure S8. Azacitidine stabilizes NSUN2 and TYMS in the ALL-PO *KMT2A*-rearranged infant acute lymphoblastic leukemia cell line. (A) Cellular thermal shift assay (CETSA) and western blot of NSUN2 and TYMS in ALL-PO cells with (+) and without (-) 100μM azacitidine treatment showing thermal stabilization of NSUN2 and TYMS in azacitidine treated samples. (B) ALL-PO cell lysates with (+) and without (-) 100μM azacitidine showing no thermal stabilization of NSUN2 or TYMS. SOD1 as loading control. (C) Western blot analysis and validation of thermal stabilization of NSUN2 in ALL-PO cells in a dose response time-course CETSA at 48°C using a range of 100–1.56μM of azacitidine in eight steps during a time-course of 10–180-minute incubations showing increasing thermal stabilization over time at different concentrations.

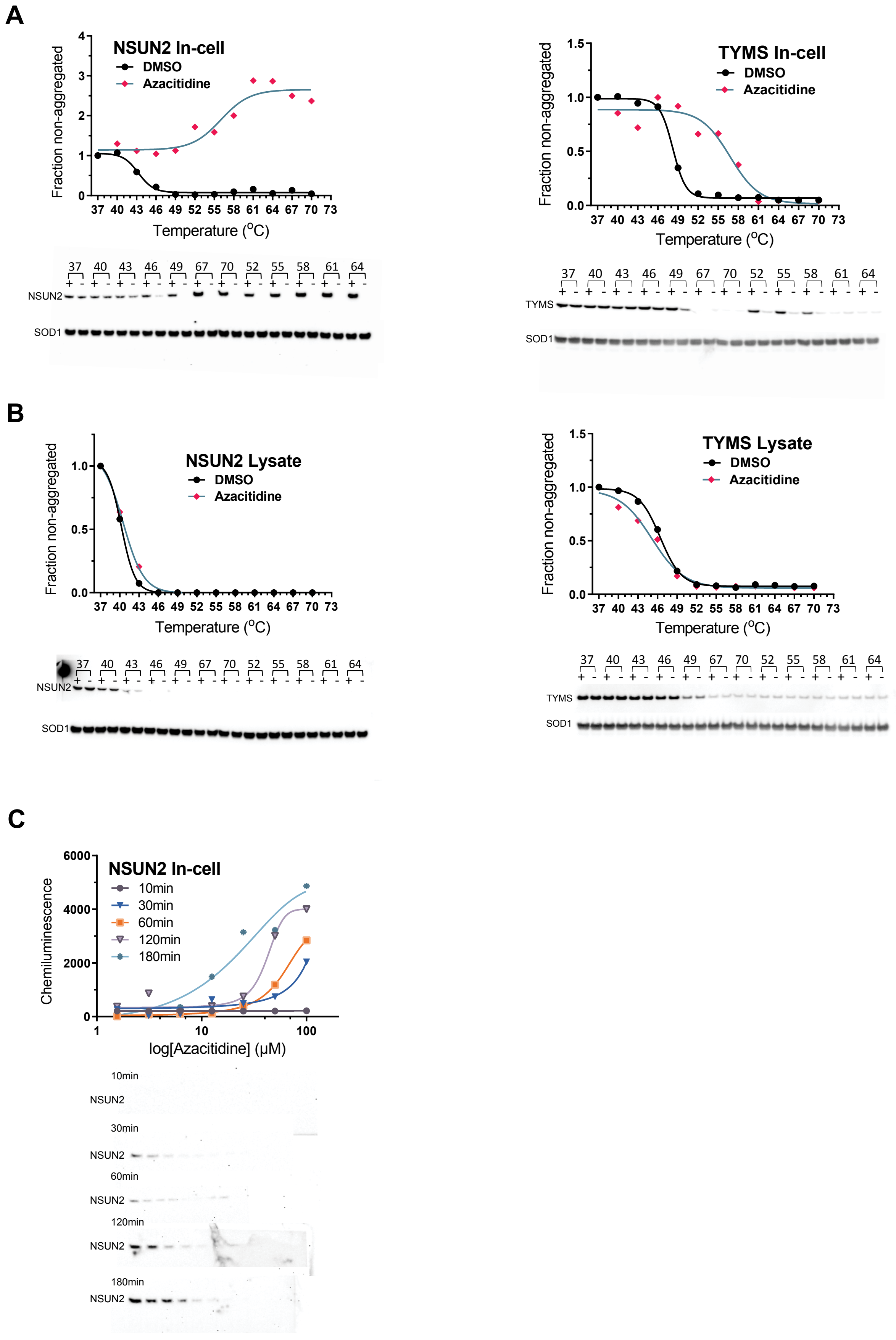

Supplemental Figure S9. Treatment of ALL-PO cells with venetoclax results in dose dependent thermal stabilization of BCL-2. Western blot following dose response cellular thermal shift assay of two independent experiments at 60°C for BCL-2. Venetoclax dose ranged from 1 to 0.001μM.

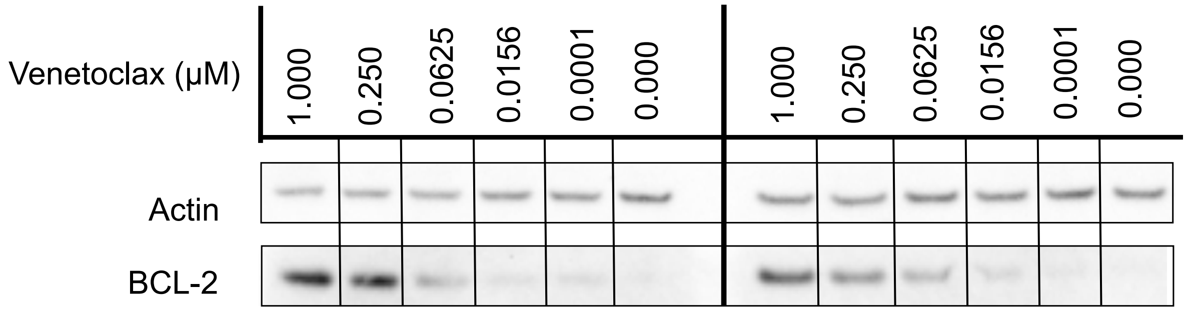

Supplemental Figure S10. Venetoclax induces apoptosis and necrosis in *KMT2A*-rearranged infant acute lymphoblastic leukemia cell lines. Cells were incubated with IC_{50} concentrations of ventoclax for 72 hours. Measurements of luminescence (apoptosis) and fluorescence (necrosis) were performed every 30 minutes.

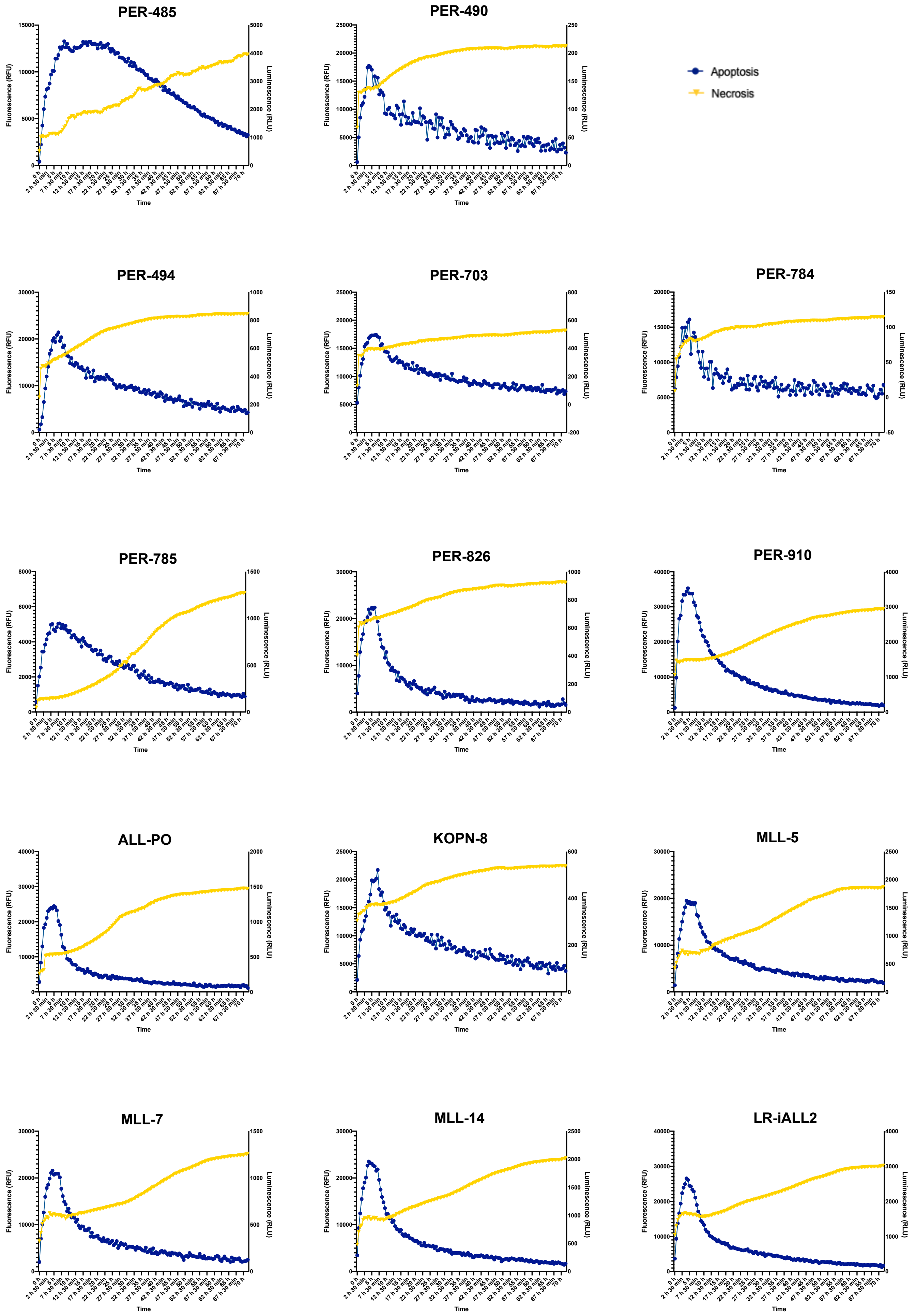

Supplemental Figure S11. The combinatorial dose response of venetoclax with (A) conventional chemotherapeutic agents or (B) hypomethylating agents compared to single agent on cell survival. Conventional chemotherapeutic agents include vincristine, daunorubicin, dexamethasone, cytarabine, methotrexate, 6-mercaptopurine, L-asparaginase, thioguanine and 4-hydroperoxycyclophosphamide (4-HPC). Hypomethylating agents include azacitidine, decitabine and zebularine.

A

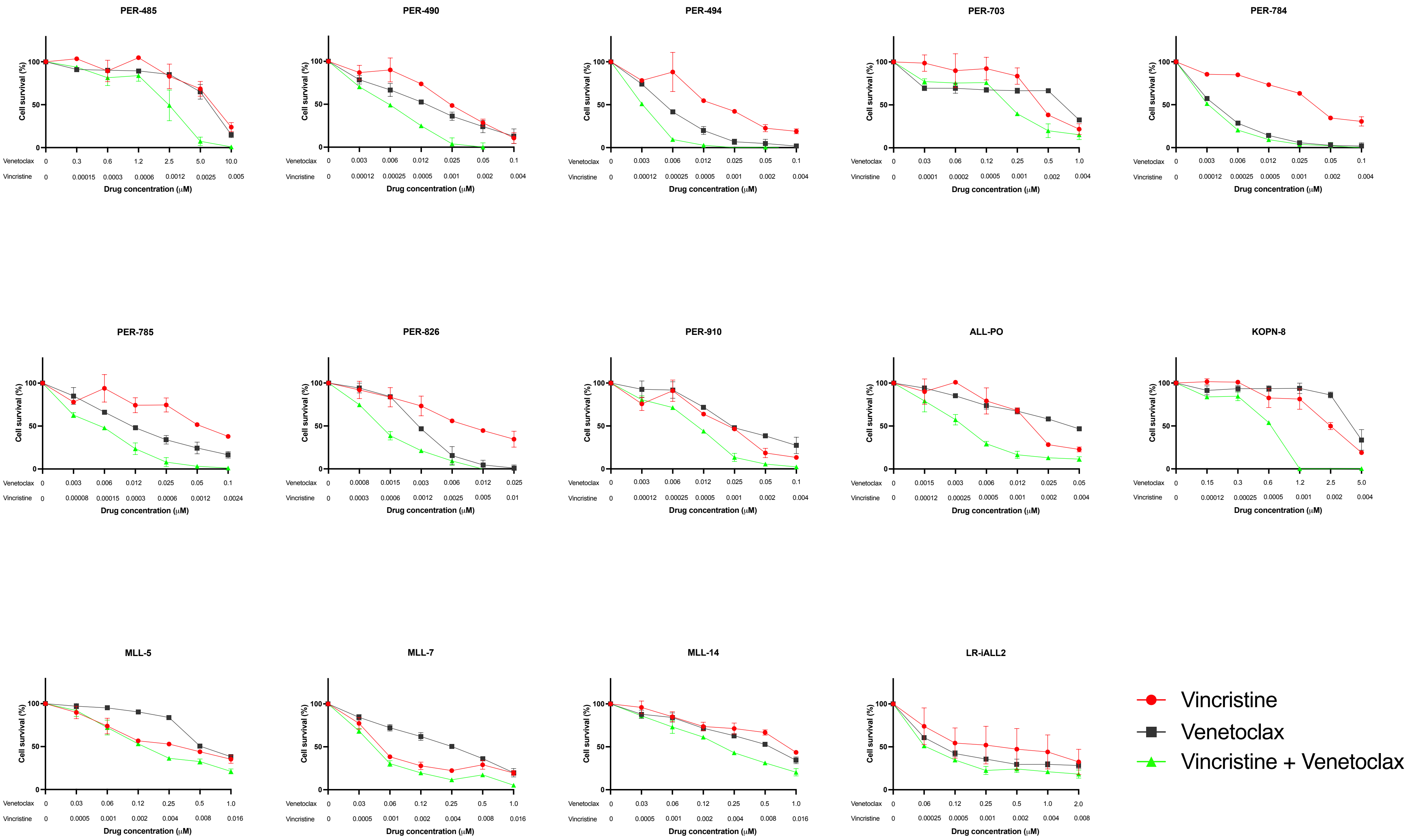

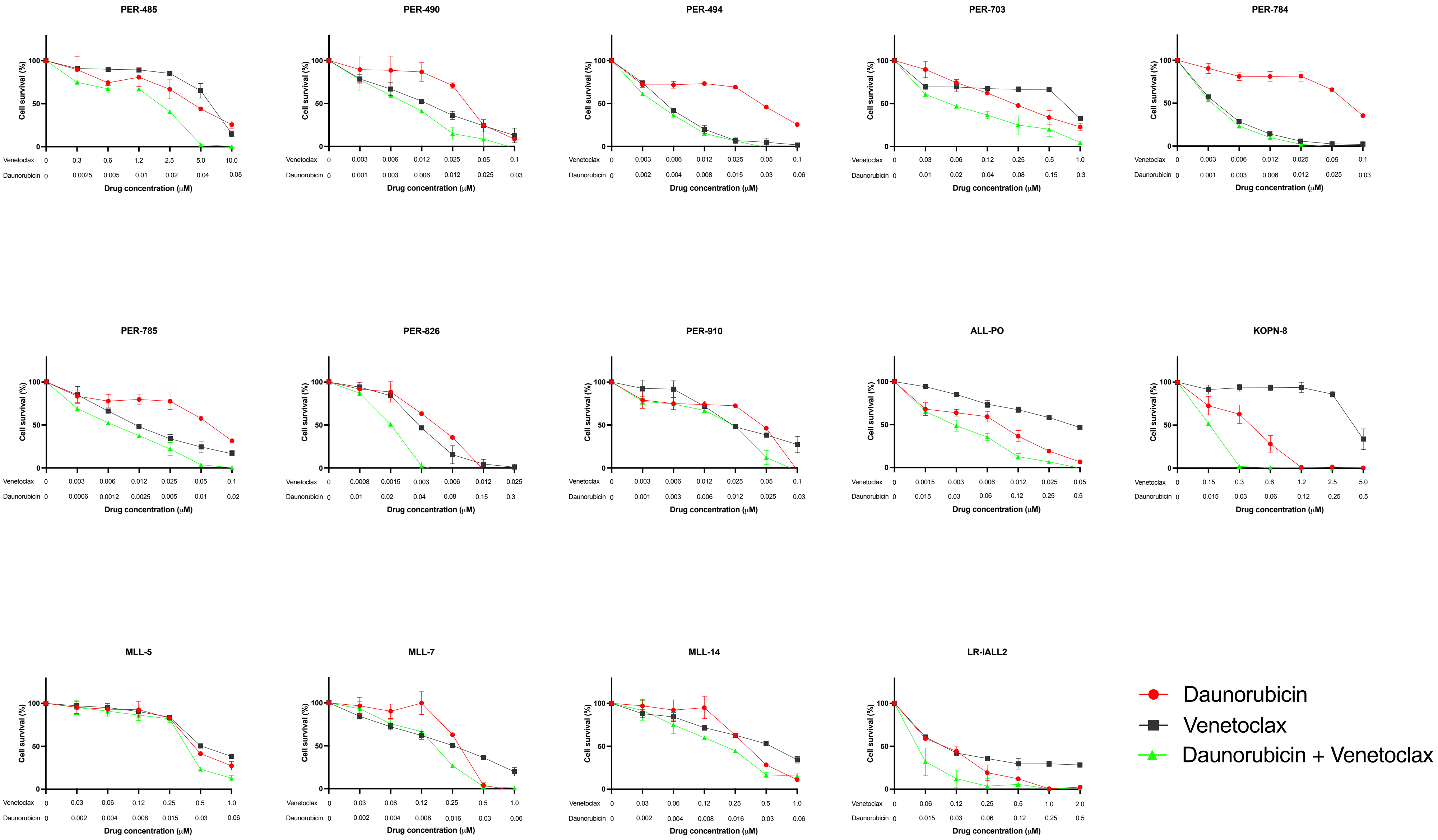
PER-485
Cell survival (%)
100
50
0
Venetoclax 0 0.3 0.6 1.2 2.5 5.0 10.0
Daunorubicin 0 0.0025 0.005 0.01 0.02 0.04 0.08
Drug concentration (μM)
PER-490
Cell survival (%)
Venetoclax 0 0.003 0.006 0.012 0.025 0.05 0.1
Daunorubicin 0 0.001 0.003 0.006 0.012 0.025 0.03
Drug concentration (μM)
PER-494
Cell survival (%)
Venetoclax 0 0.003 0.006 0.012 0.025 0.05 0.1
Daunorubicin 0 0.002 0.004 0.008 0.015 0.03 0.06
Drug concentration (μM)
PER-703
Cell survival (%)
Venetoclax 0 0.03 0.06 0.12 0.25 0.5 1.0
Daunorubicin 0 0.01 0.02 0.04 0.08 0.15 0.3
Drug concentration (μM)
PER-784
Cell survival (%)
Venetoclax 0 0.003 0.006 0.012 0.025 0.05 0.1
Daunorubicin 0 0.001 0.003 0.006 0.012 0.025 0.03
Drug concentration (μM)
PER-785
Cell survival (%)
Venetoclax 0 0.003 0.006 0.012 0.025 0.05 0.1
Daunorubicin 0 0.0006 0.0012 0.0025 0.005 0.01 0.02
Drug concentration (μM)
PER-826
Cell survival (%)
Venetoclax 0 0.0008 0.0015 0.003 0.006 0.012 0.025
Daunorubicin 0 0.01 0.02 0.04 0.08 0.15 0.3
Drug concentration (μM)
PER-910
Cell survival (%)
Venetoclax 0 0.003 0.006 0.012 0.025 0.05 0.1
Daunorubicin 0 0.001 0.003 0.006 0.012 0.025 0.03
Drug concentration (μM)
ALL-PO
Cell survival (%)
Venetoclax 0 0.0015 0.003 0.006 0.012 0.025 0.05
Daunorubicin 0 0.015 0.03 0.06 0.12 0.25 0.5
Drug concentration (μM)
KOPN-8
Cell survival (%)
Venetoclax 0 0.15 0.3 0.6 1.2 2.5 5.0
Daunorubicin 0 0.015 0.03 0.06 0.12 0.25 0.5
Drug concentration (μM)
MLL-5
Cell survival (%)
Venetoclax 0 0.03 0.06 0.12 0.25 0.5 1.0
Daunorubicin 0 0.002 0.004 0.008 0.015 0.03 0.06
Drug concentration (μM)
MLL-7
Cell survival (%)
Venetoclax 0 0.03 0.06 0.12 0.25 0.5 1.0
Daunorubicin 0 0.002 0.004 0.008 0.016 0.03 0.06
Drug concentration (μM)
MLL-14
Cell survival (%)
Venetoclax 0 0.03 0.06 0.12 0.25 0.5 1.0
Daunorubicin 0 0.002 0.004 0.008 0.016 0.03 0.06
Drug concentration (μM)
LR-iALL2
Cell survival (%)
Venetoclax 0 0.06 0.12 0.25 0.5 1.0 2.0
Daunorubicin 0 0.015 0.03 0.06 0.12 0.25 0.5
Drug concentration (μM)
Daunorubicin
Venetoclax
Daunorubicin + Venetoclax

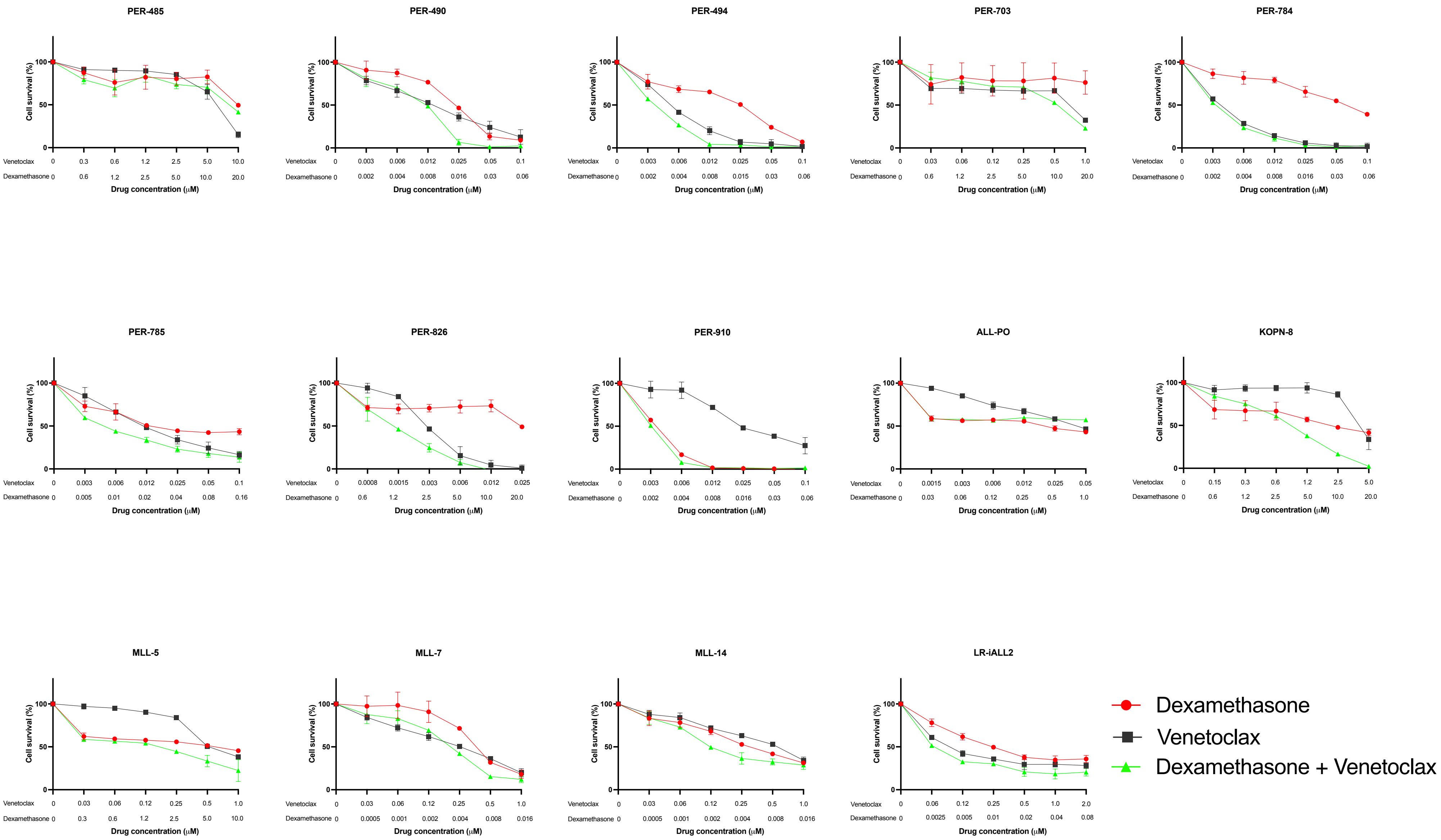
PER-485
PER-490
PER-494
PER-703
PER-784
PER-785
PER-826
PER-910
ALL-PO
KOPN-8
MLL-5
MLL-7
MLL-14
LR-iALL2
Cell survival (%)
Venetoclax
Dexamethasone
Drug concentration (μM)
Dexamethasone
Venetoclax
Dexamethasone + Venetoclax

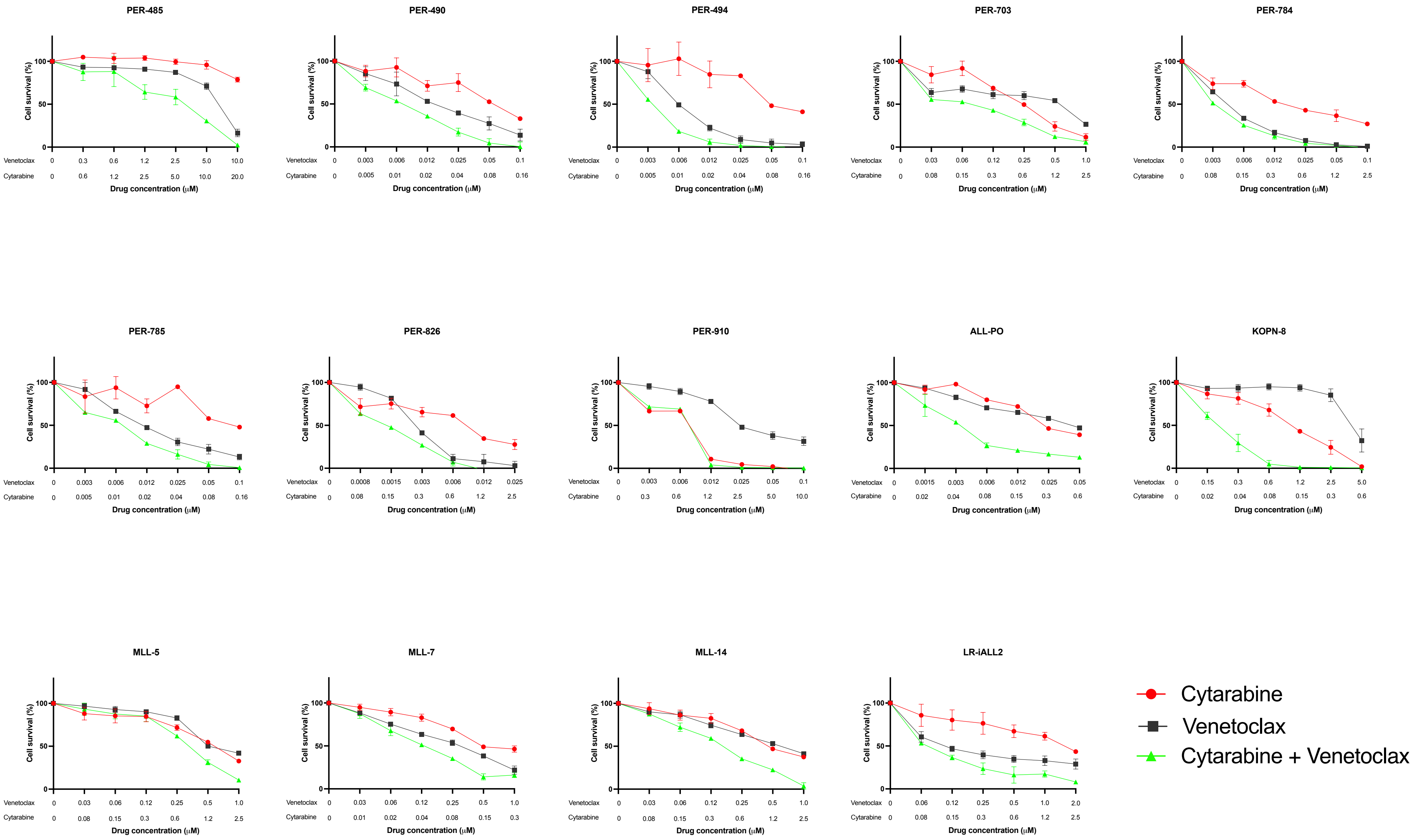
PER-485
Cell survival (%)
100
50
0
Venetoclax 0 0.3 0.6 1.2 2.5 5.0 10.0
Cytarabine 0 0.6 1.2 2.5 5.0 10.0 20.0
Drug concentration (μM)
PER-490
Cell survival (%)
Venetoclax 0 0.003 0.006 0.012 0.025 0.05 0.1
Cytarabine 0 0.005 0.01 0.02 0.04 0.08 0.16
Drug concentration (μM)
PER-494
Cell survival (%)
Venetoclax 0 0.003 0.006 0.012 0.025 0.05 0.1
Cytarabine 0 0.005 0.01 0.02 0.04 0.08 0.16
Drug concentration (μM)
PER-703
Cell survival (%)
Venetoclax 0 0.03 0.06 0.12 0.25 0.5 1.0
Cytarabine 0 0.08 0.15 0.3 0.6 1.2 2.5
Drug concentration (μM)
PER-784
Cell survival (%)
Venetoclax 0 0.003 0.006 0.012 0.025 0.05 0.1
Cytarabine 0 0.08 0.15 0.3 0.6 1.2 2.5
Drug concentration (μM)
PER-785
Cell survival (%)
Venetoclax 0 0.003 0.006 0.012 0.025 0.05 0.1
Cytarabine 0 0.005 0.01 0.02 0.04 0.08 0.16
Drug concentration (μM)
PER-826
Cell survival (%)
Venetoclax 0 0.0008 0.0015 0.003 0.006 0.012 0.025
Cytarabine 0 0.08 0.15 0.3 0.6 1.2 2.5
Drug concentration (μM)
PER-910
Cell survival (%)
Venetoclax 0 0.003 0.006 0.012 0.025 0.05 0.1
Cytarabine 0 0.3 0.6 1.2 2.5 5.0 10.0
Drug concentration (μM)
ALL-PO
Cell survival (%)
Venetoclax 0 0.0015 0.003 0.006 0.012 0.025 0.05
Cytarabine 0 0.02 0.04 0.08 0.15 0.3 0.6
Drug concentration (μM)
KOPN-8
Cell survival (%)
Venetoclax 0 0.15 0.3 0.6 1.2 2.5 5.0
Cytarabine 0 0.02 0.04 0.08 0.15 0.3 0.6
Drug concentration (μM)
MLL-5
Cell survival (%)
Venetoclax 0 0.03 0.06 0.12 0.25 0.5 1.0
Cytarabine 0 0.08 0.15 0.3 0.6 1.2 2.5
Drug concentration (μM)
MLL-7
Cell survival (%)
Venetoclax 0 0.03 0.06 0.12 0.25 0.5 1.0
Cytarabine 0 0.01 0.02 0.04 0.08 0.15 0.3
Drug concentration (μM)
MLL-14
Cell survival (%)
Venetoclax 0 0.03 0.06 0.12 0.25 0.5 1.0
Cytarabine 0 0.08 0.15 0.3 0.6 1.2 2.5
Drug concentration (μM)
LR-iALL2
Cell survival (%)
Venetoclax 0 0.06 0.12 0.25 0.5 1.0 2.0
Cytarabine 0 0.08 0.15 0.3 0.6 1.2 2.5
Drug concentration (μM)
Cytarabine
Venetoclax
Cytarabine + Venetoclax

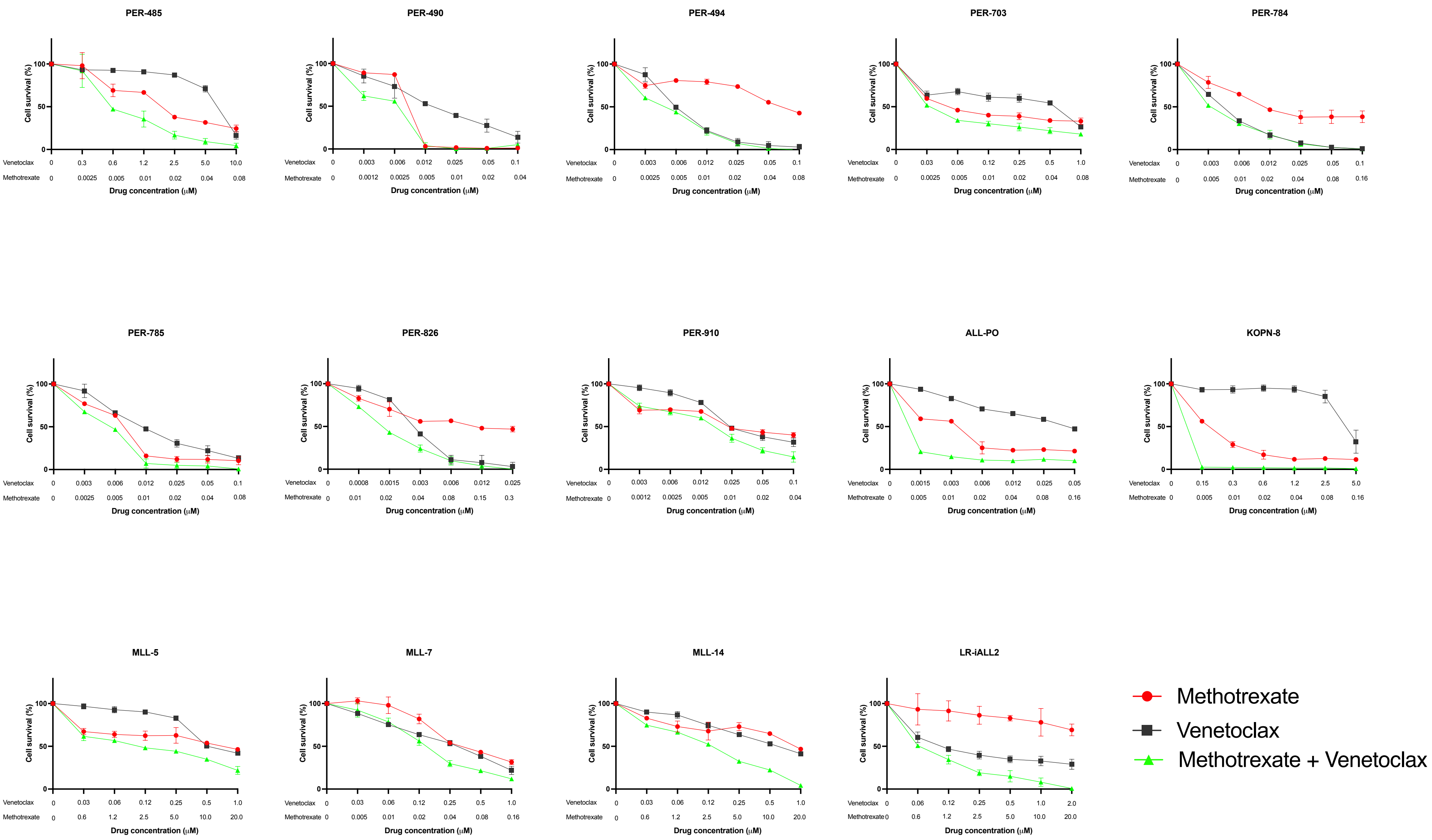
PER-485
Cell survival (%)
100
50
0
Venetoclax 0 0.3 0.6 1.2 2.5 5.0 10.0
Methotrexate 0 0.0025 0.005 0.01 0.02 0.04 0.08
Drug concentration (µM)
PER-490
Venetoclax 0 0.003 0.006 0.012 0.025 0.05 0.1
Methotrexate 0 0.0012 0.0025 0.005 0.01 0.02 0.04
PER-494
Venetoclax 0 0.003 0.006 0.012 0.025 0.05 0.1
Methotrexate 0 0.0025 0.005 0.01 0.02 0.04 0.08
PER-703
Venetoclax 0 0.03 0.06 0.12 0.25 0.5 1.0
Methotrexate 0 0.0025 0.005 0.01 0.02 0.04 0.08
PER-784
Venetoclax 0 0.003 0.006 0.012 0.025 0.05 0.1
Methotrexate 0 0.005 0.01 0.02 0.04 0.08 0.16
PER-785
Venetoclax 0 0.003 0.006 0.012 0.025 0.05 0.1
Methotrexate 0 0.0025 0.005 0.01 0.02 0.04 0.08
PER-826
Venetoclax 0 0.0008 0.0015 0.003 0.006 0.012 0.025
Methotrexate 0 0.01 0.02 0.04 0.08 0.15 0.3
PER-910
Venetoclax 0 0.003 0.006 0.012 0.025 0.05 0.1
Methotrexate 0 0.0012 0.0025 0.005 0.01 0.02 0.04
ALL-PO
Venetoclax 0 0.0015 0.003 0.006 0.012 0.025 0.05
Methotrexate 0 0.005 0.01 0.02 0.04 0.08 0.16
KOPN-8
Venetoclax 0 0.15 0.3 0.6 1.2 2.5 5.0
Methotrexate 0 0.005 0.01 0.02 0.04 0.08 0.16
MLL-5
Venetoclax 0 0.03 0.06 0.12 0.25 0.5 1.0
Methotrexate 0 0.6 1.2 2.5 5.0 10.0 20.0
MLL-7
Venetoclax 0 0.03 0.06 0.12 0.25 0.5 1.0
Methotrexate 0 0.005 0.01 0.02 0.04 0.08 0.16
MLL-14
Venetoclax 0 0.03 0.06 0.12 0.25 0.5 1.0
Methotrexate 0 0.6 1.2 2.5 5.0 10.0 20.0
LR-iALL2
Venetoclax 0 0.06 0.12 0.25 0.5 1.0 2.0
Methotrexate 0 0.6 1.2 2.5 5.0 10.0 20.0
Methotrexate
Venetoclax
Methotrexate + Venetoclax

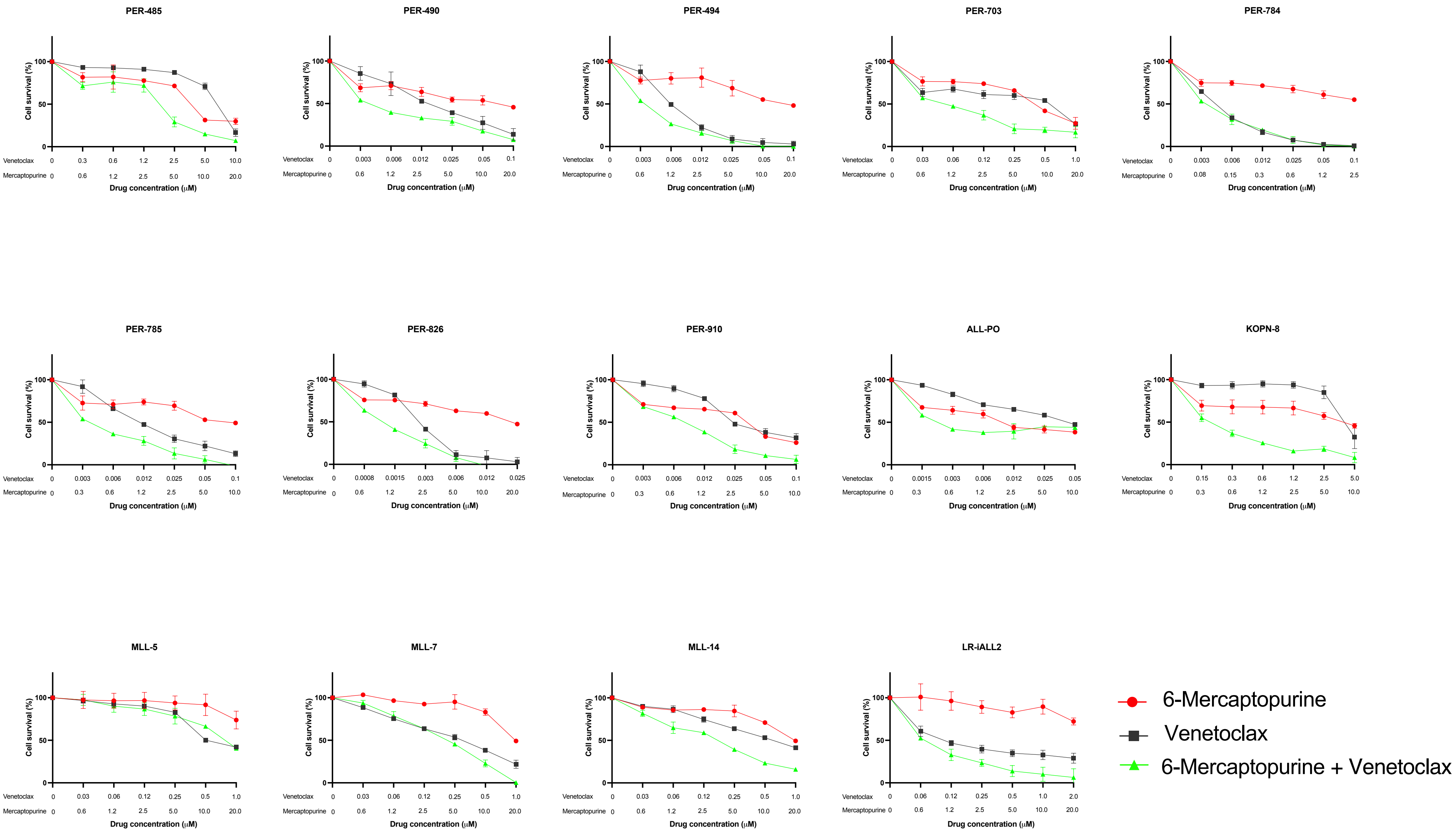
PER-485
Cell survival (%)
Venetoclax 0 0.3 0.6 1.2 2.5 5.0 10.0
Mercaptopurine 0 0.6 1.2 2.5 5.0 10.0 20.0
Drug concentration (μM)
PER-490
Venetoclax 0 0.003 0.006 0.012 0.025 0.05 0.1
Mercaptopurine 0 0.6 1.2 2.5 5.0 10.0 20.0
PER-494
Venetoclax 0 0.003 0.006 0.012 0.025 0.05 0.1
Mercaptopurine 0 0.6 1.2 2.5 5.0 10.0 20.0
PER-703
Venetoclax 0 0.03 0.06 0.12 0.25 0.5 1.0
Mercaptopurine 0 0.6 1.2 2.5 5.0 10.0 20.0
PER-784
Venetoclax 0 0.003 0.006 0.012 0.025 0.05 0.1
Mercaptopurine 0 0.08 0.15 0.3 0.6 1.2 2.5
PER-785
Venetoclax 0 0.003 0.006 0.012 0.025 0.05 0.1
Mercaptopurine 0 0.3 0.6 1.2 2.5 5.0 10.0
PER-826
Venetoclax 0 0.0008 0.0015 0.003 0.006 0.012 0.025
Mercaptopurine 0 0.6 1.2 2.5 5.0 10.0 20.0
PER-910
Venetoclax 0 0.003 0.006 0.012 0.025 0.05 0.1
Mercaptopurine 0 0.3 0.6 1.2 2.5 5.0 10.0
ALL-PO
Venetoclax 0 0.0015 0.003 0.006 0.012 0.025 0.05
Mercaptopurine 0 0.3 0.6 1.2 2.5 5.0 10.0
KOPN-8
Venetoclax 0 0.15 0.3 0.6 1.2 2.5 5.0
Mercaptopurine 0 0.3 0.6 1.2 2.5 5.0 10.0
MLL-5
Venetoclax 0 0.03 0.06 0.12 0.25 0.5 1.0
Mercaptopurine 0 0.6 1.2 2.5 5.0 10.0 20.0
MLL-7
Venetoclax 0 0.03 0.06 0.12 0.25 0.5 1.0
Mercaptopurine 0 0.6 1.2 2.5 5.0 10.0 20.0
MLL-14
Venetoclax 0 0.03 0.06 0.12 0.25 0.5 1.0
Mercaptopurine 0 0.6 1.2 2.5 5.0 10.0 20.0
LR-iALL2
Venetoclax 0 0.06 0.12 0.25 0.5 1.0 2.0
Mercaptopurine 0 0.6 1.2 2.5 5.0 10.0 20.0
6-Mercaptopurine
Venetoclax
6-Mercaptopurine + Venetoclax

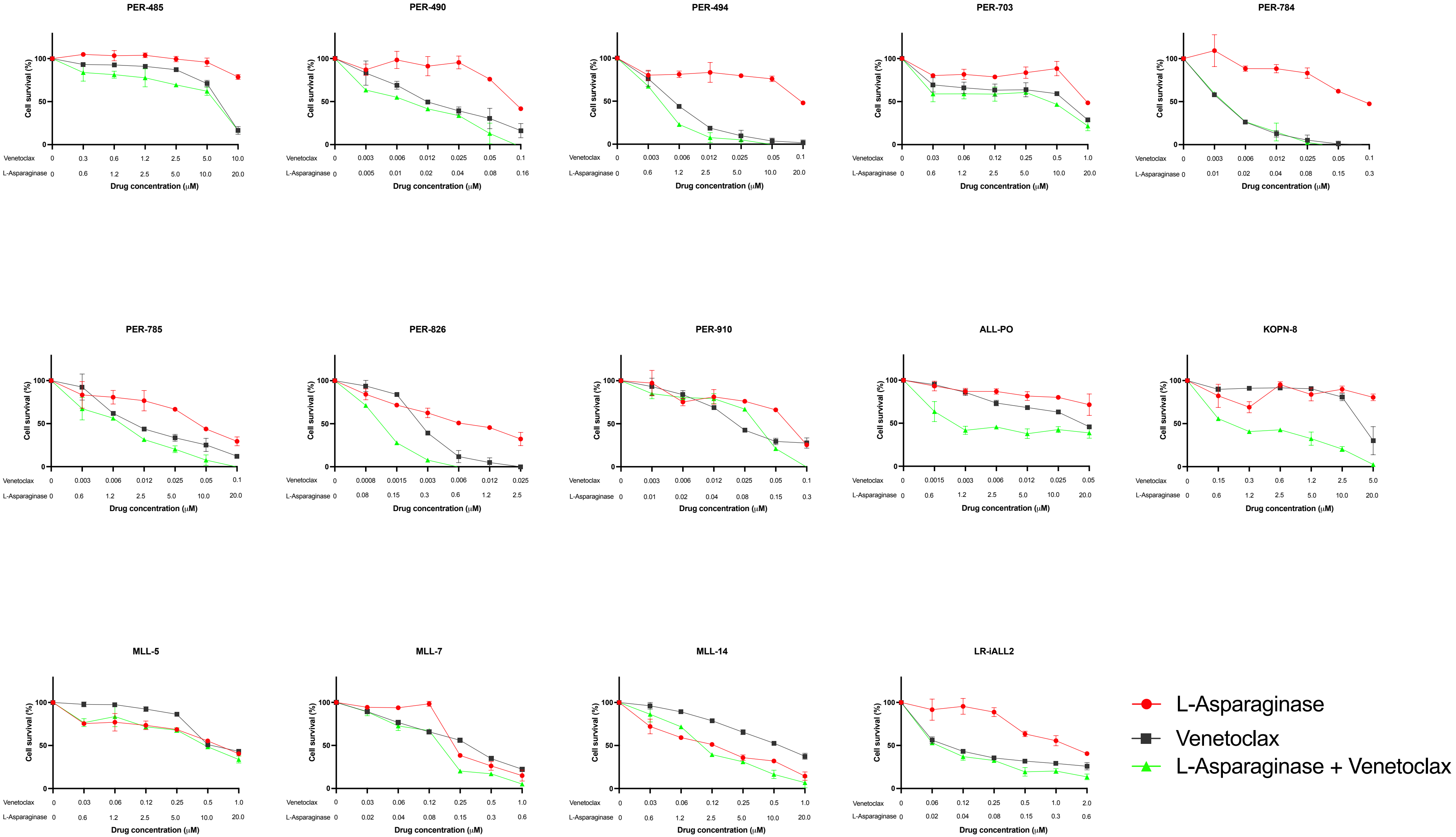
PER-485
PER-490
PER-494
PER-703
PER-784
PER-785
PER-826
PER-910
ALL-PO
KOPN-8
MLL-5
MLL-7
MLL-14
LR-iALL2
Cell survival (%)
Venetoclax
L-Asparaginase
Drug concentration (μM)
L-Asparaginase
Venetoclax
L-Asparaginase + Venetoclax

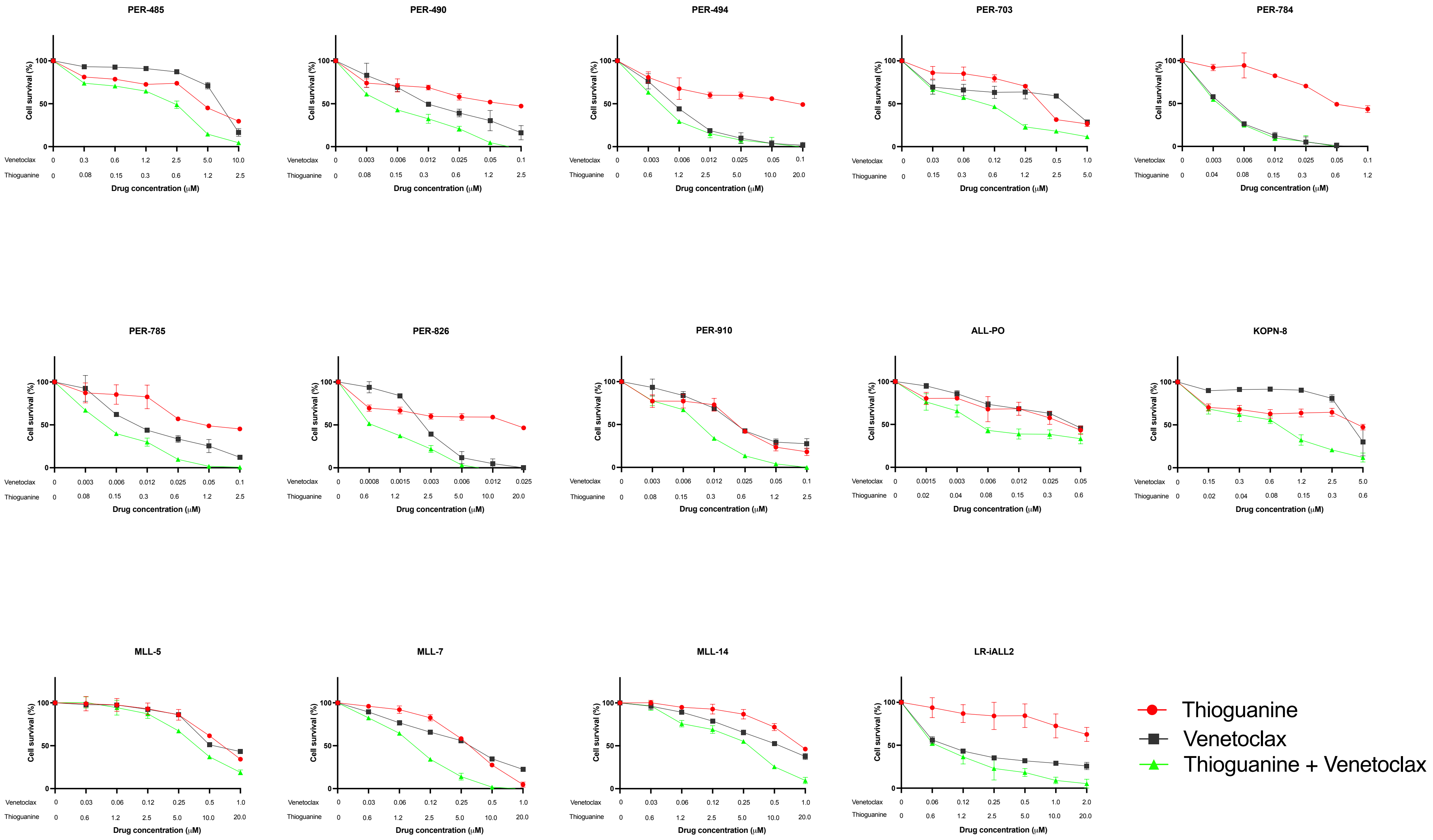

PER-485
Cell survival (%)
Venetoclax 0 0.3 0.6 1.2 2.5 5.0 10.0
Thioguanine 0 0.08 0.15 0.3 0.6 1.2 2.5
Drug concentration (μM)
PER-490
Venetoclax 0 0.003 0.006 0.012 0.025 0.05 0.1
Thioguanine 0 0.08 0.15 0.3 0.6 1.2 2.5
PER-494
Venetoclax 0 0.003 0.006 0.012 0.025 0.05 0.1
Thioguanine 0 0.6 1.2 2.5 5.0 10.0 20.0
PER-703
Venetoclax 0 0.03 0.06 0.12 0.25 0.5 1.0
Thioguanine 0 0.15 0.3 0.6 1.2 2.5 5.0
PER-784
Venetoclax 0 0.003 0.006 0.012 0.025 0.05 0.1
Thioguanine 0 0.04 0.08 0.15 0.3 0.6 1.2
PER-785
Venetoclax 0 0.003 0.006 0.012 0.025 0.05 0.1
Thioguanine 0 0.08 0.15 0.3 0.6 1.2 2.5
PER-826
Venetoclax 0 0.0008 0.0015 0.003 0.006 0.012 0.025
Thioguanine 0 0.6 1.2 2.5 5.0 10.0 20.0
PER-910
Venetoclax 0 0.003 0.006 0.012 0.025 0.05 0.1
Thioguanine 0 0.08 0.15 0.3 0.6 1.2 2.5
ALL-PO
Venetoclax 0 0.0015 0.003 0.006 0.012 0.025 0.05
Thioguanine 0 0.02 0.04 0.08 0.15 0.3 0.6
KOPN-8
Venetoclax 0 0.15 0.3 0.6 1.2 2.5 5.0
Thioguanine 0 0.02 0.04 0.08 0.15 0.3 0.6
MLL-5
Venetoclax 0 0.03 0.06 0.12 0.25 0.5 1.0
Thioguanine 0 0.6 1.2 2.5 5.0 10.0 20.0
MLL-7
Venetoclax 0 0.03 0.06 0.12 0.25 0.5 1.0
Thioguanine 0 0.6 1.2 2.5 5.0 10.0 20.0
MLL-14
Venetoclax 0 0.03 0.06 0.12 0.25 0.5 1.0
Thioguanine 0 0.6 1.2 2.5 5.0 10.0 20.0
LR-iALL2
Venetoclax 0 0.06 0.12 0.25 0.5 1.0 2.0
Thioguanine 0 0.6 1.2 2.5 5.0 10.0 20.0
Thioguanine
Venetoclax
Thioguanine + Venetoclax

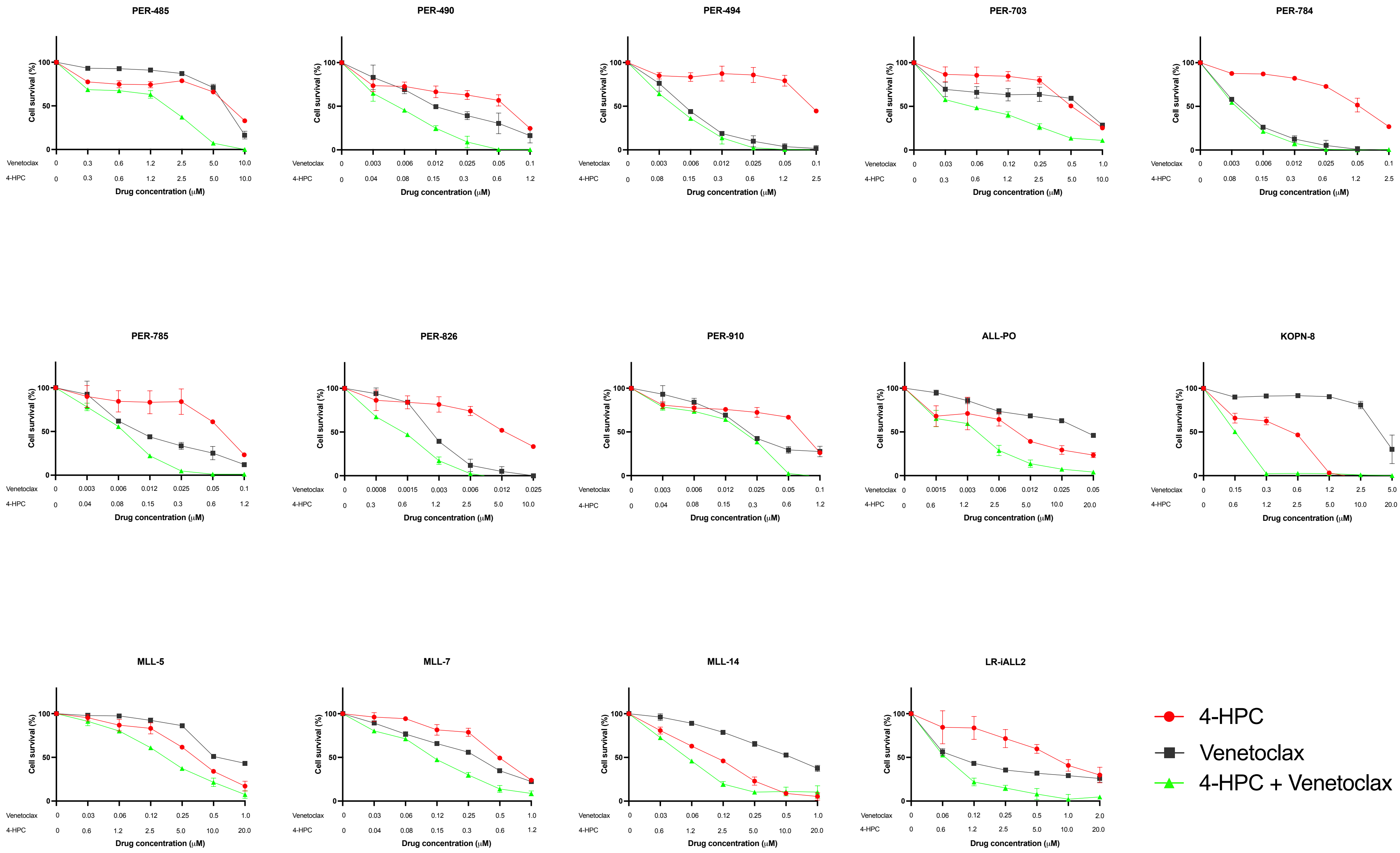
PER-485
PER-490
PER-494
PER-703
PER-784
PER-785
PER-826
PER-910
ALL-PO
KOPN-8
MLL-5
MLL-7
MLL-14
LR-iALL2
Cell survival (%)
100
50
0
Venetoclax 0 0.3 0.6 1.2 2.5 5.0 10.0
4-HPC 0 0.3 0.6 1.2 2.5 5.0 10.0
Venetoclax 0 0.003 0.006 0.012 0.025 0.05 0.1
4-HPC 0 0.04 0.08 0.15 0.3 0.6 1.2
4-HPC 0 0.08 0.15 0.3 0.6 1.2 2.5
Venetoclax 0 0.03 0.06 0.12 0.25 0.5 1.0
4-HPC 0 0.3 0.6 1.2 2.5 5.0 10.0
Venetoclax 0 0.0008 0.0015 0.003 0.006 0.012 0.025
4-HPC 0 0.3 0.6 1.2 2.5 5.0 10.0
Venetoclax 0 0.0015 0.003 0.006 0.012 0.025 0.05
4-HPC 0 0.6 1.2 2.5 5.0 10.0 20.0
Venetoclax 0 0.15 0.3 0.6 1.2 2.5 5.0
4-HPC 0 0.6 1.2 2.5 5.0 10.0 20.0
Venetoclax 0 0.06 0.12 0.25 0.5 1.0 2.0
Drug concentration (μM)
4-HPC
Venetoclax
4-HPC + Venetoclax

B

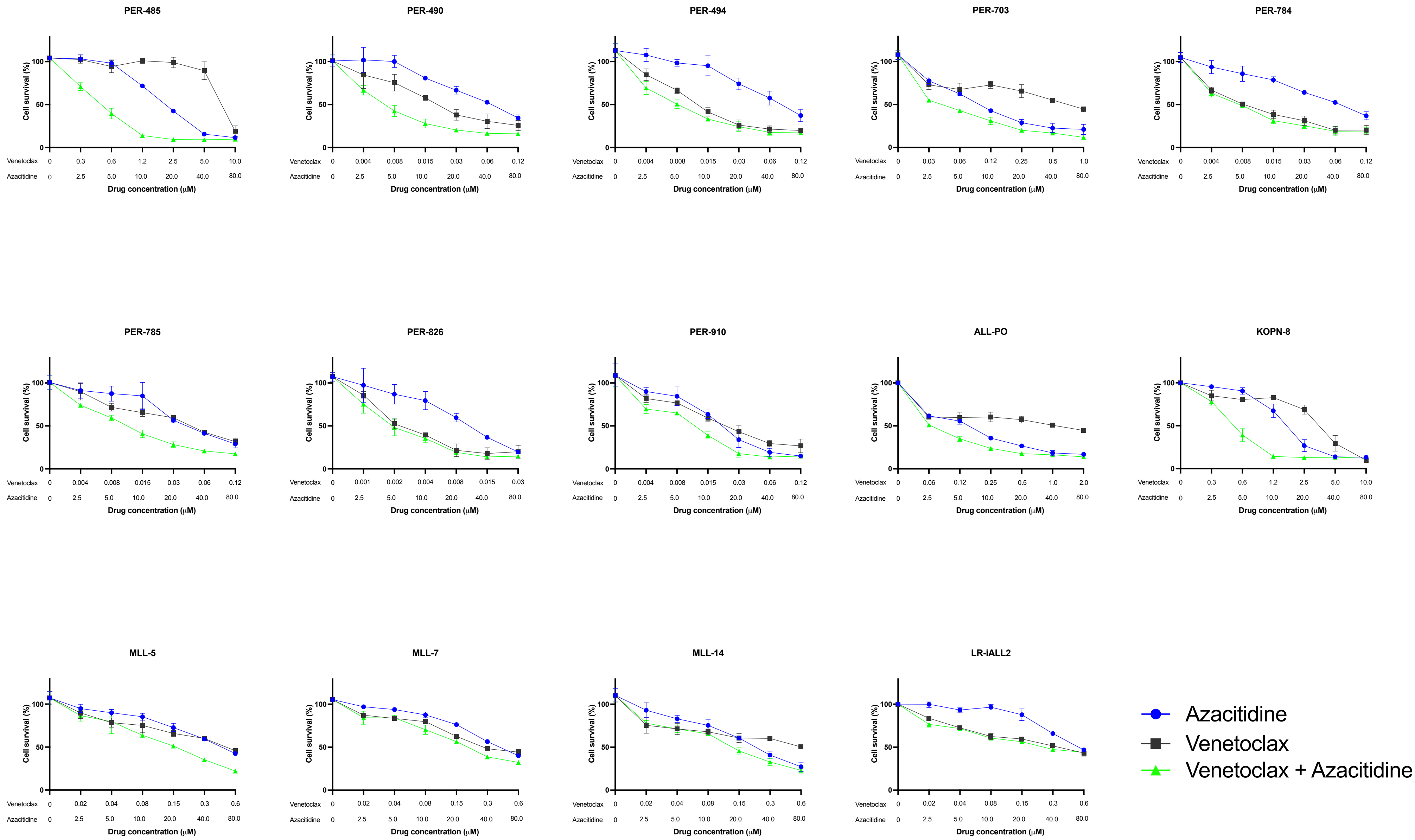
PER-485
Cell survival (%)
100
50
0
Venetoclax 0 0.3 0.6 1.2 2.5 5.0 10.0
Azacitidine 0 2.5 5.0 10.0 20.0 40.0 80.0
Drug concentration (μM)
PER-490
Cell survival (%)
100
50
0
Venetoclax 0 0.004 0.008 0.015 0.03 0.06 0.12
Azacitidine 0 2.5 5.0 10.0 20.0 40.0 80.0
Drug concentration (μM)
PER-494
Cell survival (%)
100
50
0
Venetoclax 0 0.004 0.008 0.015 0.03 0.06 0.12
Azacitidine 0 2.5 5.0 10.0 20.0 40.0 80.0
Drug concentration (μM)
PER-703
Cell survival (%)
100
50
0
Venetoclax 0 0.03 0.06 0.12 0.25 0.5 1.0
Azacitidine 0 2.5 5.0 10.0 20.0 40.0 80.0
Drug concentration (μM)
PER-784
Cell survival (%)
100
50
0
Venetoclax 0 0.004 0.008 0.015 0.03 0.06 0.12
Azacitidine 0 2.5 5.0 10.0 20.0 40.0 80.0
Drug concentration (μM)
PER-785
Cell survival (%)
100
50
0
Venetoclax 0 0.004 0.008 0.015 0.03 0.06 0.12
Azacitidine 0 2.5 5.0 10.0 20.0 40.0 80.0
Drug concentration (μM)
PER-826
Cell survival (%)
100
50
0
Venetoclax 0 0.001 0.002 0.004 0.008 0.015 0.03
Azacitidine 0 2.5 5.0 10.0 20.0 40.0 80.0
Drug concentration (μM)
PER-910
Cell survival (%)
100
50
0
Venetoclax 0 0.004 0.008 0.015 0.03 0.06 0.12
Azacitidine 0 2.5 5.0 10.0 20.0 40.0 80.0
Drug concentration (μM)
ALL-PO
Cell survival (%)
100
50
0
Venetoclax 0 0.06 0.12 0.25 0.5 1.0 2.0
Azacitidine 0 2.5 5.0 10.0 20.0 40.0 80.0
Drug concentration (μM)
KOPN-8
Cell survival (%)
100
50
0
Venetoclax 0 0.3 0.6 1.2 2.5 5.0 10.0
Azacitidine 0 2.5 5.0 10.0 20.0 40.0 80.0
Drug concentration (μM)
MLL-5
Cell survival (%)
100
50
0
Venetoclax 0 0.02 0.04 0.08 0.15 0.3 0.6
Azacitidine 0 2.5 5.0 10.0 20.0 40.0 80.0
Drug concentration (μM)
MLL-7
Cell survival (%)
100
50
0
Venetoclax 0 0.02 0.04 0.08 0.15 0.3 0.6
Azacitidine 0 2.5 5.0 10.0 20.0 40.0 80.0
Drug concentration (μM)
MLL-14
Cell survival (%)
100
50
0
Venetoclax 0 0.02 0.04 0.08 0.15 0.3 0.6
Azacitidine 0 2.5 5.0 10.0 20.0 40.0 80.0
Drug concentration (μM)
LR-iALL2
Cell survival (%)
100
50
0
Venetoclax 0 0.02 0.04 0.08 0.15 0.3 0.6
Azacitidine 0 2.5 5.0 10.0 20.0 40.0 80.0
Drug concentration (μM)
Azacitidine
Venetoclax
Venetoclax + Azacitidine

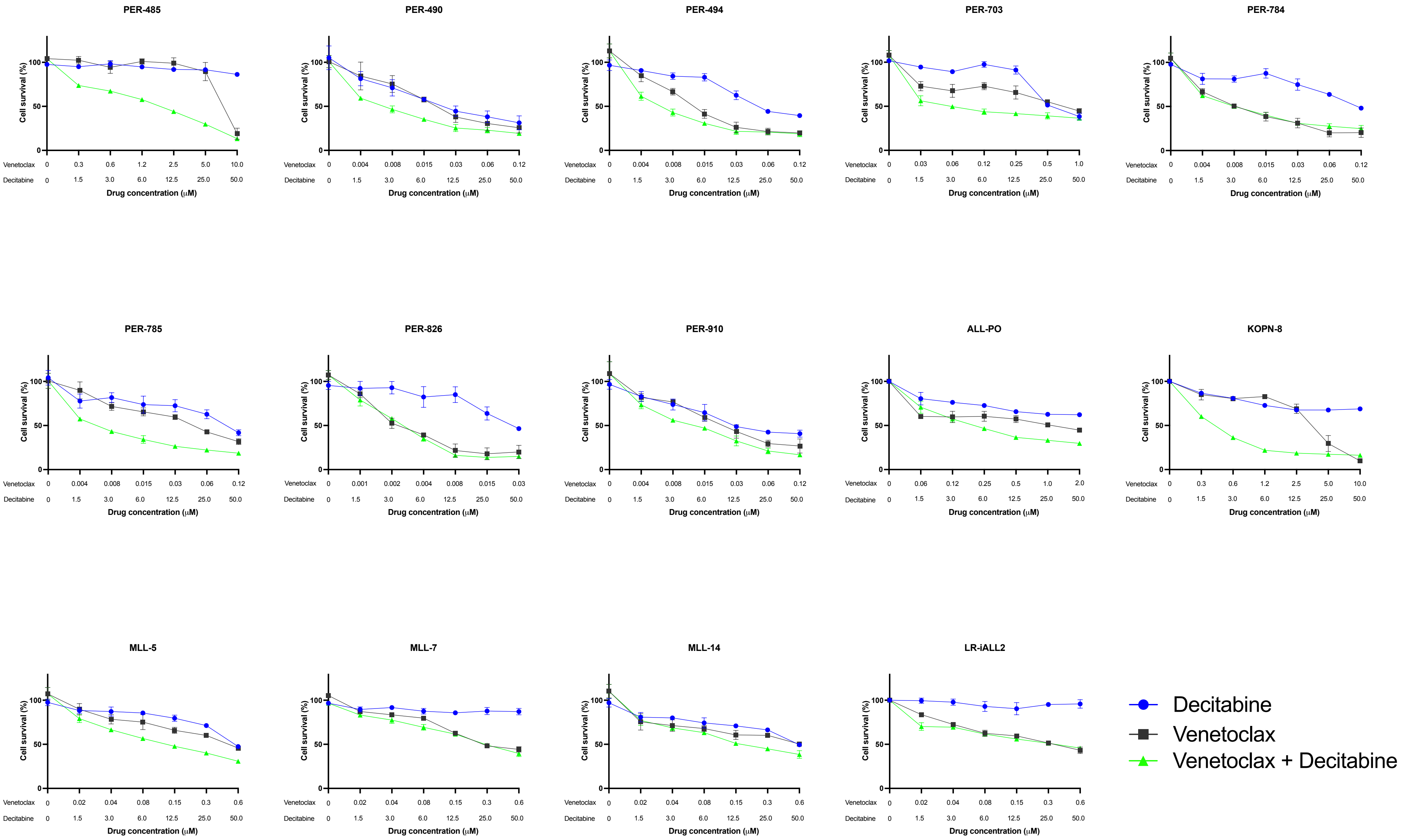
PER-485
Cell survival (%)
Venetoclax 0 0.3 0.6 1.2 2.5 5.0 10.0
Decitabine 0 1.5 3.0 6.0 12.5 25.0 50.0
Drug concentration (μM)
PER-490
Venetoclax 0 0.004 0.008 0.015 0.03 0.06 0.12
Decitabine 0 1.5 3.0 6.0 12.5 25.0 50.0
PER-494
Venetoclax 0 0.004 0.008 0.015 0.03 0.06 0.12
Decitabine 0 1.5 3.0 6.0 12.5 25.0 50.0
PER-703
Venetoclax 0 0.03 0.06 0.12 0.25 0.5 1.0
Decitabine 0 1.5 3.0 6.0 12.5 25.0 50.0
PER-784
Venetoclax 0 0.004 0.008 0.015 0.03 0.06 0.12
Decitabine 0 1.5 3.0 6.0 12.5 25.0 50.0
PER-785
Venetoclax 0 0.004 0.008 0.015 0.03 0.06 0.12
Decitabine 0 1.5 3.0 6.0 12.5 25.0 50.0
PER-826
Venetoclax 0 0.001 0.002 0.004 0.008 0.015 0.03
Decitabine 0 1.5 3.0 6.0 12.5 25.0 50.0
PER-910
Venetoclax 0 0.004 0.008 0.015 0.03 0.06 0.12
Decitabine 0 1.5 3.0 6.0 12.5 25.0 50.0
ALL-PO
Venetoclax 0 0.06 0.12 0.25 0.5 1.0 2.0
Decitabine 0 1.5 3.0 6.0 12.5 25.0 50.0
KOPN-8
Venetoclax 0 0.3 0.6 1.2 2.5 5.0 10.0
Decitabine 0 1.5 3.0 6.0 12.5 25.0 50.0
MLL-5
Venetoclax 0 0.02 0.04 0.08 0.15 0.3 0.6
Decitabine 0 1.5 3.0 6.0 12.5 25.0 50.0
MLL-7
Venetoclax 0 0.02 0.04 0.08 0.15 0.3 0.6
Decitabine 0 1.5 3.0 6.0 12.5 25.0 50.0
MLL-14
Venetoclax 0 0.02 0.04 0.08 0.15 0.3 0.6
Decitabine 0 1.5 3.0 6.0 12.5 25.0 50.0
LR-iALL2
Venetoclax 0 0.02 0.04 0.08 0.15 0.3 0.6
Decitabine 0 1.5 3.0 6.0 12.5 25.0 50.0
Decitabine
Venetoclax
Venetoclax + Decitabine

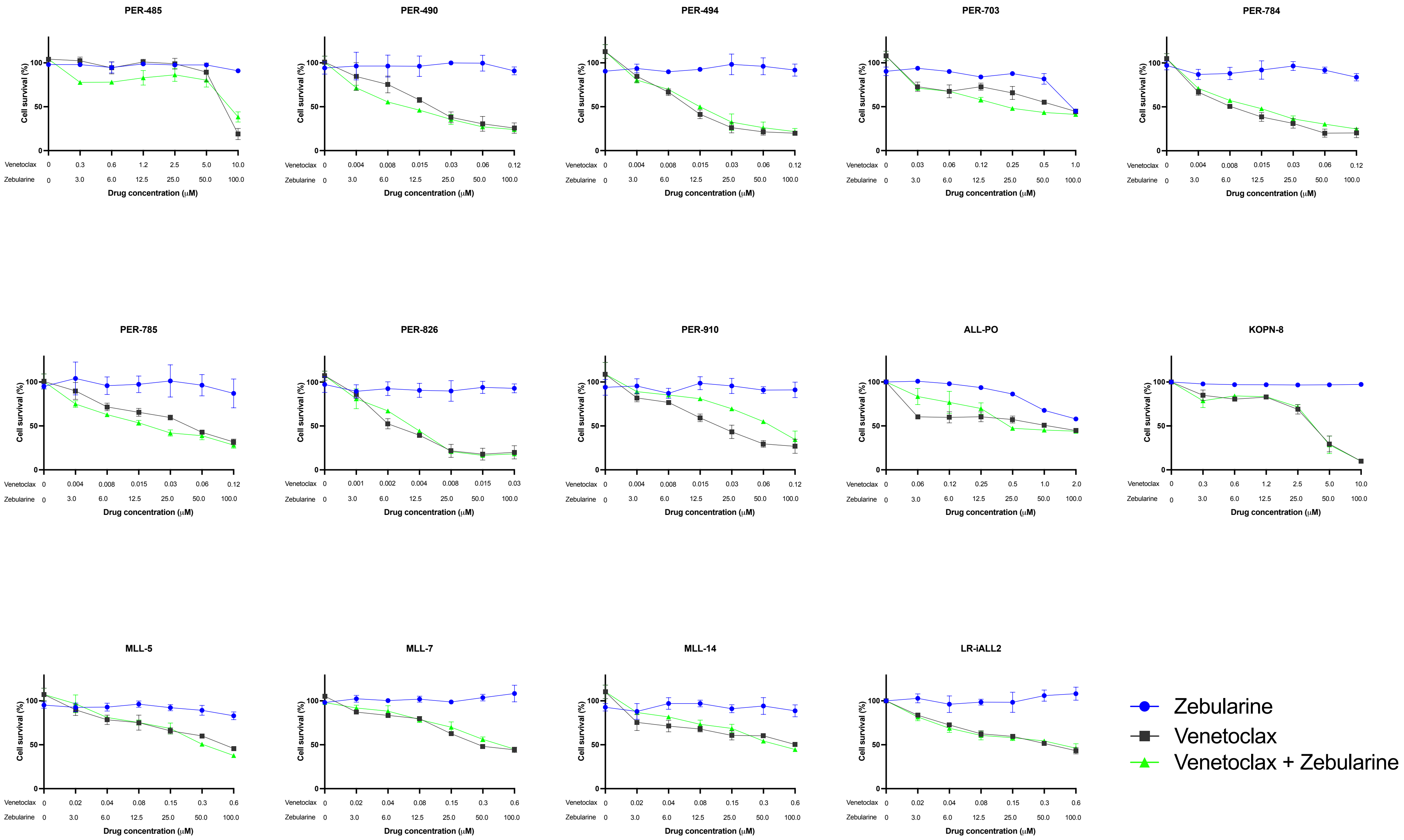
PER-485
Cell survival (%)
Venetoclax 0 0.3 0.6 1.2 2.5 5.0 10.0
Zebularine 0 3.0 6.0 12.5 25.0 50.0 100.0
Drug concentration (μM)
PER-490
Venetoclax 0 0.004 0.008 0.015 0.03 0.06 0.12
Zebularine 0 3.0 6.0 12.5 25.0 50.0 100.0
PER-494
Venetoclax 0 0.004 0.008 0.015 0.03 0.06 0.12
Zebularine 0 3.0 6.0 12.5 25.0 50.0 100.0
PER-703
Venetoclax 0 0.03 0.06 0.12 0.25 0.5 1.0
Zebularine 0 3.0 6.0 12.5 25.0 50.0 100.0
PER-784
Venetoclax 0 0.004 0.008 0.015 0.03 0.06 0.12
Zebularine 0 3.0 6.0 12.5 25.0 50.0 100.0
PER-785
Venetoclax 0 0.004 0.008 0.015 0.03 0.06 0.12
Zebularine 0 3.0 6.0 12.5 25.0 50.0 100.0
PER-826
Venetoclax 0 0.001 0.002 0.004 0.008 0.015 0.03
Zebularine 0 3.0 6.0 12.5 25.0 50.0 100.0
PER-910
Venetoclax 0 0.004 0.008 0.015 0.03 0.06 0.12
Zebularine 0 3.0 6.0 12.5 25.0 50.0 100.0
ALL-PO
Venetoclax 0 0.06 0.12 0.25 0.5 1.0 2.0
Zebularine 0 3.0 6.0 12.5 25.0 50.0 100.0
KOPN-8
Venetoclax 0 0.3 0.6 1.2 2.5 5.0 10.0
Zebularine 0 3.0 6.0 12.5 25.0 50.0 100.0
MLL-5
Venetoclax 0 0.02 0.04 0.08 0.15 0.3 0.6
Zebularine 0 3.0 6.0 12.5 25.0 50.0 100.0
MLL-7
Venetoclax 0 0.02 0.04 0.08 0.15 0.3 0.6
Zebularine 0 3.0 6.0 12.5 25.0 50.0 100.0
MLL-14
Venetoclax 0 0.02 0.04 0.08 0.15 0.3 0.6
Zebularine 0 3.0 6.0 12.5 25.0 50.0 100.0
LR-iALL2
Venetoclax 0 0.02 0.04 0.08 0.15 0.3 0.6
Zebularine 0 3.0 6.0 12.5 25.0 50.0 100.0
Zebularine
Venetoclax
Venetoclax + Zebularine
